# Homogeneous diet of contemporary Japanese inferred from stable isotope ratios of hair

**DOI:** 10.1038/srep33122

**Published:** 2016-09-12

**Authors:** Soichiro Kusaka, Eriko Ishimaru, Fujio Hyodo, Takashi Gakuhari, Minoru Yoneda, Takakazu Yumoto, Ichiro Tayasu

**Affiliations:** 1 Research Institute for Humanity and Nature, Kyoto 603-8047, Japan; 2 Museum of Natural and Environmental History, Shizuoka, Shizuoka 422-8017, Japan; 3 Hiroshima University Museum, Hiroshima University, Hiroshima 739-8524, Japan; 4 Research Core for Interdisciplinary Sciences, Okayama University, Okayama 700-8530, Japan; 5 School of Medicine, Kitazato University, Kanagawa 252-0374, Japan; 6 The University Museum, The University of Tokyo, Tokyo 113-0033, Japan; 7 Primate Research Institute, Kyoto University, Aichi 484-8506, Japan; 8 Center for Ecological Research, Kyoto University, Otsu 520-2113, Japan

## Abstract

The globalization of food production and distribution has homogenized human dietary patterns irrespective of geography, but it is uncertain how far this homogenization has progressed. This study investigated the carbon and nitrogen isotope ratios in the scalp hair of 1305 contemporary Japanese and found values of −19.4 ± 0.6‰ and 9.4 ± 0.6‰ (mean ± SD), respectively. Within Japan, the inter-regional differences for both isotope ratios was less than 1‰, which indicates low dietary heterogeneity among prefectural divisions. The carbon and nitrogen isotope ratios of the hair showed a significant correlation with the results of questionnaires on self-reported dietary habits. The carbon isotope ratios from Japan were lower than those in samples from the USA but higher than those in samples from Europe. These differences stem from the varying dietary proportions of food products originally derived from C_3_ and C_4_ plants. The dietary variation of Japan is as small as those of Europe and USA and smaller than those of some Asian countries. These results indicate that dietary homogeneity has progressed in Japan, which may indicate the influence from the spread of the Western-style diet and food globalization, although dietary heterogeneity among countries is still preserved.

During the widespread dispersion of humanity across the world, dietary patterns developed that were adapted to local environments. However, the recent globalization of food production has served to homogenize these divergent dietary patterns^1^,^2^. In accordance with the worldwide urbanization and westernization of lifestyles, the dietary patterns in developing countries have tended to change to a Western style^3^. This is known as nutrition transition and is defined by the increased consumption of dietary products high in fats and sweeteners, a lower intake of cereals, and the insufficient consumption of vegetables and fruit^1^,^4^. The nutrition transition due to globalization is expected to lead to dietary convergence across the world. However, it is recognized that the local food production and food consumption practices within a particular regional culture can be retained despite the progression of food globalization^5^. This is known as “glocalization,” which means that local food products are used to produce globally distributed food items by global companies^6^. The Western-style diet is spreading throughout the world, but people in each country can modify the imported dietary style while retaining their traditional diet.

In Japan, food consumption is influenced by westernization and the globalization of food production and distribution^3^,^7^,^8^. *Washoku* (i.e., Japanese cuisine) uses traditional Japanese foods, rice, and fresh vegetables with a low rate of meat consumption and attractive, seasonally based presentations^9^. In 1980, *washoku* showed an ideal nutritional balance of protein, fat, and carbohydrates for the dietary ecology of humans^9^. In 2013, “Washoku, traditional dietary cultures of the Japanese, notably for the celebration of New Year” was registered on the Representative List of the Intangible Cultural Heritage of Humanity of UNESCO^9^. The drastic change to a Western-style diet started after World War II^10^. The traditional Japanese diet changed between 1950 and 1975 with the increased intake of milk, meat, poultry, eggs, and fat by several fold and decreased intake of barley, potatoes, and rice^7^. The increased intake of animal meat and fat corresponded to the high economic growth period of 1960–1972 in Japan^7^,^11^. During this time, food supply chains kept in frozen or cold storage by supermarkets (i.e., cold chains) expanded throughout Japan^10^. This rapid change in dietary intake can be considered to be the time when dietary habits were westernized. The height and weight of the Japanese people consequently increased, and the incidence of cancer increased as the life expectancy was extended^7^. After 1975, the supply of meat and fat increased, and the supply of carbohydrates decreased^8^,^11^. Although the consumption of fish also increased until 1975, the amount of imported fish started increasing around 1990^12^. The quantity of food products imported from other countries also increased, and the food self-sufficiency rate decreased^11^. Thus, the globalization of food production and distribution served to homogenize the regional differences in dietary consumption. However, it is difficult to investigate the extent to which the Japanese diet has changed in response to the expanded availability of domestic and imported foods.

Stable carbon and nitrogen isotope analysis of living organisms is able to estimate the dietary sources and trophic level of an organism^13^,^14^,^15^,^16^. Analyzing the carbon and nitrogen isotopes in hair is useful for clarifying the dietary sources of humans and animals^17^,^18^,^19^,^20^,^21^. Carbon isotope ratios of hair suggest the type of plants being consumed directly by humans and indirectly by animals. C_3_ terrestrial plants, which include rice and vegetables, show carbon isotope ratios of −26.5 ± 2‰, whereas the levels in C_4_ plants, which include corn and millet, are −12.5 ± 1‰^22^,^23^,^24^. The carbon isotope ratios in animals increase 0–2‰ per trophic level^25^,^26^. Nitrogen isotope ratios of hair suggest the trophic level of food sources consumed by humans^21^. The nitrogen isotope ratios in animals increase 3.4 ± 1.1‰ per trophic level^27^. Based on the longer food chain in marine ecosystems, the nitrogen isotope ratios of marine fish are higher than those of terrestrial plants and animals. Keratin, which is a main protein in hair, is synthesized from amino acids in the hair follicles^21^,^28^. A change in diet is reflected in human beard hair in as little as 6–12 days^20^. Studies have indicated that the isotopic values of keratin extensively reflect the isotopic values of proteins, although isotope enrichment occurs between dietary sources and human hair^19^. The isotope ratios in hair are also influenced by the physiology and health of human population^29^. Because human hair is suitable for obtaining tissue samples of modern humans, many studies have used hair as a typical sample for all body tissue^19^,^20^,^30^. Carbon and nitrogen isotope ratios have been reported for hair samples from the USA^17^,^18^,^31^, Brazil^22^, UK^32^,^33^, Germany^34^, other European countries^35^,^36^, and Asian countries^37^. The carbon and nitrogen isotope ratios of hair from Japan in the 1930s–1950s^38^ and in 1984–1985^19^ have also been reported, and recent samples have been evaluated with respect to mercury concentrations for the purpose of assessing public health^39^,^40^. Recently, a global dataset of the isotope ratios in human hair samples was analyzed^41^ to provide an isotopic perspective of dietary homogeneity around the world.

The purpose of this study was to characterize the diet of contemporary Japanese by using the carbon and nitrogen isotope ratios in hair. This study tested whether the globalization of food production and distribution has homogenized the diet of the populations in the various regions of Japan (Fig. 1). We also considered the variation in diet in relation to the sex or age of an individual. We compared the isotope ratios in hair from Japan with those from other countries to characterize the dietary composition of contemporary Japanese on a global scale.

## Results

### Variation in δ^13^C and δ^15^N of contemporary Japanese hair samples

The mean δ^13^C value of the Japanese hair samples was −19.4 ± 0.6 ‰ (mean ± 1 standard deviation), and the range was −22.9‰ to −16.3‰. The mean δ^15^N value of the Japanese hair samples was 9.4 ± 0.6, and the range was 6.7‰ to 12.7‰ (Table 1). The results of the isotope analyses of food samples are listed in Table 2. Figure 2 shows the carbon and nitrogen isotope analysis results for Japanese hair and food samples. The isotope enrichment values, or the enrichment between food and human hair (2.5‰ for δ^13^C, and 4.1‰ for δ^15^N), were added to the isotopic values of food samples^19^. The δ^15^N values of marine and freshwater fish are higher than those of C_3_ terrestrial plants, dairy products, and other animals. The δ^13^C values of beef, pork, and egg are higher than those of C_3_ terrestrial plants. Tofu and natto made from legumes exhibit lower carbon and nitrogen isotope ratios than animal products. Beef from Japan shows intermediate values between beef from Australia, which is fed a high proportion of C_3_ terrestrial plants, and beef from USA, which is fed a high proportion of C_4_ plants^42^. Consequently, beef in Japan shows carbon isotope ratios similar to those of marine fish with lower nitrogen isotope ratios by about 3‰ (Fig. 2).

### Variation in δ^13^C and δ^15^N of hair samples in Japan

The carbon and nitrogen isotope ratio results of Eastern and Western Japan were compared (Table 1). The Eastern samples had lower mean δ^13^C and mean δ^15^N values than the Western samples (*t*-tests: *t* = 3.03, *P* = 0.0025; and *t* = 5.82, *P* < 0.0001, respectively). In addition, there were significant differences between subsamples divided by the eight regions of Japan (Table 1; ANOVA, *F* = 8.07, *P* < 0.0001 for δ^13^C; *F* = 7.13, *P* < 0.0001 for δ^15^N). The samples from Hokkaido showed the lowest mean δ^13^C value (−19.7 ± 0.6‰) and highest mean δ^15^N value (9.7 ± 0.6‰). The samples from the Chugoku and Shikoku regions exhibited a relatively high mean δ^13^C value (−19.2 ± 0.6‰) and higher mean δ^15^N value (9.6 ± 0.6‰). The samples from the Tohoku and Kanto regions showed the lowest δ^15^N values (9.3‰). The mean carbon and nitrogen isotope ratios divided by the administrative divisions of Japan were compared (Supplementary Table S1). The carbon isotope ratios of samples from Kochi (−18.9 ± 0.8‰), Mie (−19.1 ± 0.5‰), and Yamaguchi (−19.1 ± 0.6‰) Prefectures were high, and the samples from Hokkaido (−19.7 ± 0.6‰), Fukushima (−19.6 ± 0.5‰), and Saitama (−19.6 ± 0.5‰) Prefectures are low. The nitrogen isotope ratios of the Hyogo (9.9 ± 0.6‰), Nagasaki (9.9 ± 1.0‰), and Miyazaki (9.8 ± 0.4‰) Prefectures are high, and the Fukushima (9.0 ± 0.5‰), Fukui (9.1 ± 0.5‰), and Saitama (9.2 ± 0.5‰) Prefectures are low.

### Effect of age and sex on δ^13^C and δ^15^N of contemporary Japanese hair samples

The mean δ^13^C value among adult males (≥20 years old) was −19.2 ± 0.6‰, and the mean δ^15^N was 9.5 ± 0.6‰ (Table 1). The mean δ^13^C value for adult females was −19.5 ± 0.6‰, and the mean δ^15^N was 9.3 ± 0.5‰. Cumulative frequency plots for the carbon and nitrogen isotope ratios showed the difference in isotopic distributions of males and females (Fig. 3A,B). The δ^13^C and δ^15^N values of the adult males were significantly higher than those of the adult females (*t*-test, *t* = 8.65, *P* < 0.001 for δ^13^C; *t* = 5.19, *P* < 0.001 for δ^15^N).

The mean δ^13^C value for minor males (0–19 years old) was −19.2 ± 0.5‰, and the mean δ^15^N was 9.6 ± 0.7‰ (Table 2). The mean δ^13^C and δ^15^N values for minor females were −19.3 ± 0.6‰ and 9.7 ± 0.7‰, respectively. The δ^13^C and δ^15^N values for minor males were not significantly different from those of minor females (*t*-test, *t* = 1.38, *P* = 0.17 for δ^13^C; *t* = −0.60, *P* = 0.55 for δ^15^N).

The age of the males did not show a significant correlation with the δ^13^C values, but the age of the females did show a significant correlation (Fig. 4; *R*^*2*^ = 0.045, P < 0.0001). Meanwhile, the age for males showed a significant correlation with the δ^15^N values (*R*^2^ = 0.016, P < 0.001), but the age for females was not significantly correlated. Infants (0–1 years old, N = 8) showed a δ^13^C value of −18.8 ± 0.4‰ and δ^15^N value of 11.9 ± 0.6. These values are higher than the mean values for adult females by 0.7‰ for δ^13^C and 2.6‰ for δ^15^N, which indicates the dietary incorporation of their mothers’ milk.

The analysis of covariance showed that the isotopic variation can be explained by two regressions with different slopes for males and females, excluding eight infants and two adults of unknown age (ANCOVA: *F* = 36.7, *P* < 0.0001 for δ^13^C; *F* = 14.3, *P* < 0.0001 for δ^15^N). The effects of sex, age, and the interaction between sex and age showed significant influences on the δ^13^C values (sex: *F* = 74.6, *P* < 0.0001, age: *F* = 19.4, *P* < 0.0001, interaction between sex and age: *F* = 8.8, *P* = 0.0031) and δ^15^N values (sex: *F* = 25.6, *P* = 0.0002, age: *F* = 13.6, *P* < 0.0001, interaction between sex and age: *F* = 8.0, *P* = 0.0047). The post hoc *t*-test on the difference of least squares means (LSMean) showed a significant difference between males and females (LSMean δ^13^C for males = −19.2, LSMean δ^13^C for females = −19.5, *t* = 8.63, *P* < 0.05; LSMean δ^15^N for males = 9.5, LSMean δ^13^C for females = 9.4, *t* = 5.05, *P* < 0.05). This suggests that sex affects the isotopic variations in contemporary Japanese hair, although the effect of age on the isotopic variations differs according to sex.

### Correspondence of δ^13^C and δ^15^N in Japanese hair samples to the questionnaire on dietary habits

To investigate if the stable isotope ratios are reflective of diet, we asked hair donors to complete a questionnaire on their dietary habits. They were asked to record how often on average they consumed ten foods [beef, pork, chicken, egg, marine fish, freshwater fish, tofu (legume product), natto (fermented legume), milk, and cheese] each week (0–7 times). The carbon and nitrogen isotope ratios of the Japanese hair samples and the results of the questionnaire on dietary habits were assessed by multiple regression analysis. The δ^15^N values were positively correlated with the incorporation of marine fish and beef and negatively correlated with tofu and natto (*R*^2^ = 0.12, *F* = 18.1, *P* < 0.01 for δ^15^N; Supplementary Table S2). The δ^13^C values of the hair samples were positively correlated with beef, marine fish, and eggs and negatively correlated with natto and tofu (*R*^*2*^ = 0.06, *F* = 8.23, *P* < 0.01 for δ^13^C). These results indicate that the individuals who frequently ate marine fish and beef showed higher carbon and nitrogen isotope ratios in hair, and the individuals who frequently ate tofu and natto showed lower carbon and nitrogen isotope ratios.

### Comparison of δ^13^C and δ^15^N levels in Japanese hair samples with other countries

For comparison, we measured the carbon and nitrogen isotope ratios of samples from South Korea, India, and Mongolia. The mean δ^13^C value of hair samples from South Korea was −19.1 ± 0.6‰, and the mean δ^15^N value was 9.7 ± 0.5‰. The mean δ^13^C value of hair samples from India was −20.6 ± 0.9‰, and the mean δ^15^N value was 7.4 ± 0.6‰. The mean δ^13^C value of hair samples from Mongolia was −20.7 ± 0.5‰, and the mean δ^15^N value was 10.2 ± 0.6‰.

The hair samples from contemporary Japanese were compared with other previously published data (Supplementary Table S3 and Fig. 5). The mean nitrogen isotope ratio was lower than that of the Japanese in 1984–1985^19^, which suggests that the incorporation of marine fish in the diet has decreased or consumption of terrestrial resources has increased. The mean carbon and nitrogen isotope ratios of the Ainu during the 1930s–1950s are almost the same as those of contemporary Japanese^38^. This implies that their diet incorporated similar levels of marine fish. Compared with the data from the USA^31^, Europe^36^, and Asian countries^37^, the nitrogen isotope ratios of the Japanese samples were slightly higher than those from the USA and Europe. The carbon isotope ratios showed conflicting results: lower than that of the USA and higher than that of the Europe. This indicates that the incorporation of C_4_ plants and animals fed with C_4_ plants is lower than that of the USA and higher than that of Europe.

## Discussion

The carbon and nitrogen stable isotope ratios in the human hair of contemporary Japanese are scattered and fall within the range of surveyed food sources (Fig. 2). Therefore, the diet of contemporary Japanese results from the isotope mixing of these food sources. The carbon isotope ratios of the hair samples are midway between those of C_3_ terrestrial plants, C_4_ plants, and animals raised with varying proportions of C_4_ silage. The nitrogen isotope ratios of the hair samples are lower than those of marine fish and higher than those of plants. Minagawa^19^ proposed that the Japanese diet is derived from five sources: C_3_ terrestrial plants, including rice and vegetables; legumes that fix nitrogen; C_4_ plants, including corn and millet; the meat and milk of livestock; and marine and freshwater fish. The carbon and nitrogen isotope ratios of contemporary Japanese hair is characterized by the mixing of these sources in the diet.

The carbon and nitrogen isotope ratios of Eastern and Western Japan were compared. The Eastern samples showed significantly lower mean δ^13^C and δ^15^N values (Table 1), which suggests that the people in Eastern Japan consume more terrestrial plants. This result is consistent with a 2009 study on expenditure in Japan, which revealed that more natto is consumed in Eastern Japan^43^. The diet in Eastern Japan is also characterized by the higher consumption of marine foods and lower consumption of meat^43^ (Supplementary Table S6). However, this difference would not be detected in the results of this study because the carbon and nitrogen isotopic differences between marine fish and beef in Japan show similar values (Fig. 2). In addition, there were significant differences between the subsamples divided by the eight regions of Japan. The samples from the Chugoku and Shikoku regions of Western Japan exhibited relatively high mean δ^13^C and δ^15^N values. This may be caused by the incorporation of marine foods from the Seto Inland Sea, where marine animals show relatively high levels of δ^15^N^44^. The samples from the Tohoku and Kanto regions showed the lowest δ^15^N values, which suggests that these populations consume less marine food or more terrestrial plants, particularly leguminous plants (tofu and natto). The latter interpretation is supported by the results of the study on expenditure, which showed high natto consumption in Tohoku and Kanto^43^. The carbon and nitrogen isotope ratios were divided into the administrative divisions of Japan, and the results were compared (Supplementary Table S1). The range of mean δ^13^C values was 0.7‰, and the range of mean δ^15^N values was 0.9‰. Although slight isotopic differences were found among the administrative divisions, the differences in the isotopic signatures were small.

We found isotopic variations in the hair samples based on age and sex. Adult males had higher carbon and nitrogen isotope ratios than adult females. This suggests that adult males consume more marine fish, C_4_ plants, and animal meat, whereas adult females consume more protein originating from C_3_ terrestrial plants. Another possibility is that the physiological differences between males and females are the reason for the differing isotopic enrichment values between diet and hair. The isotopic enrichment of carbon and nitrogen isotope ratios occurs in the metabolic process of an organism^27^,^45^. Differential nutritional stress^46^,^47^, positive nitrogen balance during growth^29^, or a difference in urea excretion rates^27^,^48^ would cause a difference in the isotopic enrichment factors at the individual level of different life stages. Some physiological demands on dietary proteins due to sex-related phenomena may cause the isotopic difference between males and females. This needs further testing in future research. Infants (0–1 year old) exhibited high nitrogen isotope ratios, which suggests that nitrogen may be incorporated through their mothers’ milk^49^. A report by the Ministry of Health, Labour and Welfare stated that 1-month-old Japanese infants are fed by breastfeeding (42.4%), infant formula (5.1%), or a mix of the two previous choices (52.5%)^50^. The guide on breastfeeding and weaning in Japan recommends that infants should start consuming infant formula at 5–6 months and complete weaning at 12–18 months^50^. Although the infant samples may have started incorporating infant formula, their nitrogen isotope ratios exhibited signals of isotope enrichment. The carbon isotope ratios of females decreased with age, which may be attributed to the decreasing consumption of C_4_ plants and meat. The nitrogen isotope ratios of males increased with age, which suggests the increasing dietary incorporation of marine fish. These results may reflect the greater dependence of younger people on the Western-style diet, whereas older people still consume the traditional Japanese diet^7^.

The carbon and nitrogen isotope ratios in the Japanese hair samples and the results of the questionnaires on dietary habits showed a significant correlation after multiple regression analysis. The δ^15^N values were positively correlated with the incorporation of marine fish and beef and negatively correlated with tofu and natto. This is a reasonable result because marine fish and beef contain higher nitrogen isotope ratios, whereas tofu and natto, which are made from legumes, show lower nitrogen isotope ratios. The δ^13^C values of the hair samples were positively correlated with beef, marine fish, and eggs and negatively correlated with natto and tofu. This is also reasonable because beef, marine fish, and eggs show relatively high carbon isotope ratios and natto and tofu have lower carbon isotope ratios. These results indicate that the carbon and nitrogen isotope ratios of human hair can be explained by the dietary habits of each individual. When assessing dietary intake, the accuracy of self-reporting of dietary habits yields significant uncertainty^51^. The isotopic values in human hair are independent and reliable evidence of dietary composition directly consumed by each individual and represent suitable samples for studying human dietary ecology.

The standard deviation of carbon and nitrogen isotope ratios of hair samples among contemporary Japanese was small (±0.6‰). This means that the dietary variation from an isotopic point of view is very small. We found a small difference (<1‰) in the mean values for hair among the administrative divisions in Japan. This suggests that regional differences in diet are extremely small, and the diet of contemporary Japanese may have become homogenized with the globalization of dietary products and developments in food distribution. Small isotopic variations in the hair samples were also found in the analyses from other industrialized countries. The variation in carbon isotope ratios among European countries is less than 0.7‰, and the variation in nitrogen isotope ratios is 0.6‰^36^. The variation in carbon isotope ratios in samples from the USA is 0.8‰, and that of nitrogen is 0.4‰^36^. These results indicate that industrialized countries, which include Japan, Europe, and the USA, show small isotopic variations as a result of the globalization of both food products and their distribution. On the other hand, large isotopic variations (>1.0‰) were reported for the samples from China, India, and Mongolia^37^. These countries may retain local dietary habits without a fully developed food distribution system. For example, peoples in the northern region of China tend to eat millet (a C_4_ plant), but peoples in the southern region tend to eat rice (a C_3_ terrestrial plant)^52^. The comparison of the isotopic variation between Japan and other countries indicated that Japan has a smaller dietary variation.

The carbon and nitrogen isotope ratios of contemporary Japanese are lower than those of Japanese samples from 1984–1985^19^ (Fig. 5). This difference can be rationalized by considering Japanese food statistics. The supply of proteins from marine foods relative to livestock decreased from 0.84 in 1985 to 0.60 in 2007^53^. This may indicate that the change in the relative proportion of marine-based food consumption is the cause for the decreased isotope ratios from 1984 to 2007. Another possibility is the shift in the supply of cereals and meat from the USA (a C_4_-based ecosystem) to China (a C_3_-based ecosystem), which resulted in the lower carbon isotope ratios among contemporary Japanese. In 1988, the USA and China supplied 32.2% and 6.5%, respectively, of cereals and meat to Japan; in 2002, the USA and China supplied 26.0% and 14.3%, respectively^54^. Because Japan has a low food self-sufficiency rate (39% in 2010)^55^, the isotopic dietary composition is sensitive to any changes in the suppliers of imported foods.

The carbon isotope ratios of Japan are lower than those of the USA and higher than those of Europe. Since Japan incorporates foods from C_3_ and C_4_ ecosystems, the carbon and nitrogen isotope ratios of contemporary Japanese display intermediate values between those of the USA and Europe. The nitrogen isotope ratios of hair samples from contemporary Japanese are slightly higher than those from the USA and Europe, which implies the greater incorporation of marine fish relative to meat. The carbon isotope ratios of hair samples from contemporary Japanese are higher than those from China, India, and Mongolia because most of the food in those countries comes from their C_3_ ecosystems^37^, whereas food products from C_4_ ecosystems are imported into Japan. The samples from India, which include vegans, showed particularly low nitrogen isotope ratios. The samples from people in Mongolia, who eat the meat and milk of sheep almost daily, exhibited high nitrogen isotope ratios. The isotopic variation in the hair from Asian countries was due to the diversity of the ecology and food customs.

The diet of the prehistoric population of the Japanese archipelago during the Jomon period (ca. 16,500–2300 years BP) has been investigated based on human and faunal bone collagen^56^,^57^. A wide range of dietary customs that were adapted to the various types of environments in Japan was revealed. The Jomon people in Hokkaido had higher nitrogen isotope ratios than Jomon people in other areas, which indicates a diet high in marine mammals and fish^56^. The Jomon people who lived in the coastal areas of the mainland consumed mixed diets with C_3_ terrestrial plants, terrestrial mammals, and marine fish^56^,^58^. The Jomon people who lived in inland areas of the mainland consumed terrestrial resources with little consumption of marine-based foods^57^. The range of carbon isotope ratios from Hokkaido to the mainland was 8.2‰ (from −22.3‰ to −14.1‰), and the range of nitrogen isotope ratios was 13.6‰ (from 5.4‰ to 19.0‰^19^). Meanwhile, the range of carbon isotope ratios in contemporary Japanese hair is 6.6‰, and the range of nitrogen isotope ratios is 6.0‰. Thus, ranges among contemporary Japanese are smaller than those among Jomon-era populations. The variations in the carbon and nitrogen isotope ratios of the populations in the Jomon period were ±0.1–1.9‰ (one standard deviation) and ±0.1–2.3‰, respectively^56^. Compared with the Jomon data, the isotopic variation of contemporary Japanese is very small. These results indicate that the Jomon people had adapted to incorporate locally collected foods, whereas contemporary Japanese have a more homogeneous diet owing to the development of food distribution and imports from other countries.

As globalization has progressed in developed countries, food products have been increasingly distributed and consumed independent of geography. The Japanese diet was progressively influenced by the Western-style diet during 1950–1975^7^. This diet high in meat and fat resulted in physical changes and a high incidence of cancer in Japan^8^,^11^. Fish consumption also increased in 1950–1975, and the import of fish increased from 1990^12^. The Japanese cuisine in 1980 exhibited an ideal nutritional balance for human dietary ecology^9^. The food self-sufficiency rate has decreased, and a great deal of imported food and domestic food are distributed via cold chains throughout Japan^11^. The correlation between stable isotope ratios of contemporary Japanese hair and the results of questionnaires on dietary habits suggests that the isotopic composition of hair is highly reflective of individual dietary habits. The standard deviations for the carbon and nitrogen isotope ratios of contemporary Japanese hair were as small as those of the samples from Europe and USA and smaller than that of samples from some Asian countries. These results suggest a homogenous dietary variation in Japan and may indicate a need to reconsider our diet from the viewpoints of human dietary ecology and dietary history.

## Methods

### Ethics statement

All experimental methods were carried out in accordance with the guidelines of the ethics committee of the Research Institute for Humanity and Nature. All experimental protocols were approved by said ethics committee. All donors were anonymous volunteers who gave informed consent prior to study enrollment. Every precaution was taken to protect the privacy of their personal information.

### Hair sample collection

**Figure 1:**
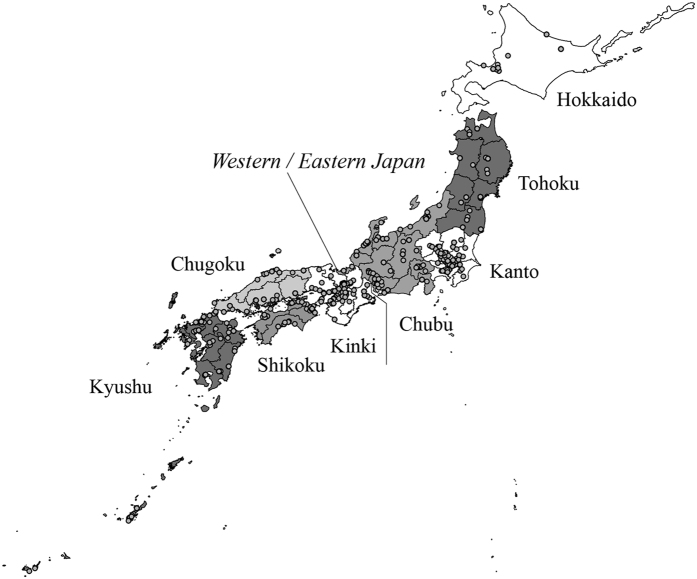
Geographic locales of hair sample donors using the ArcGIS software (ESRI Inc., ver. 10.0: http://www.esri.com). The longitude and latitude of the donors were acquired from the addresses in questionnaires (city and prefecture) by using the CSV geocoding service of the Center for Spatial Information Science of the University of Tokyo (http://newspat.csis.u-tokyo.ac.jp/geocode/). The map of Japan was developed by modifying a digital map from the Geospatial Information Authority of Japan and ESRI Japan. Prefectures are grouped into eight regions in Japan; the boundary between eastern and western Japan lies between the Kinki and Chubu regions.

**Figure 2:**
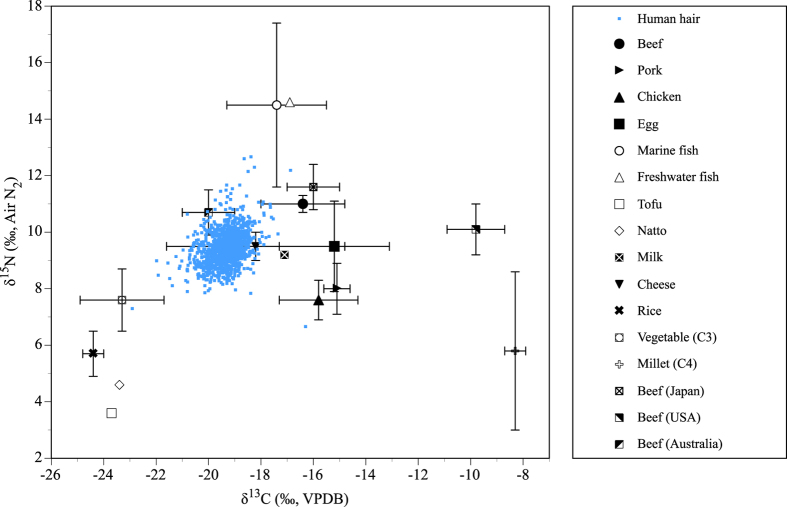
Carbon and nitrogen isotope ratios of hair samples from contemporary Japanese donors and food samples. The isotope enrichment values, that is, the difference in values between food and human hair (2.5‰ for δ^13^C, and 4.1‰ for δ^15^N), were added to the isotopic values of food samples^19^.

**Figure 3:**
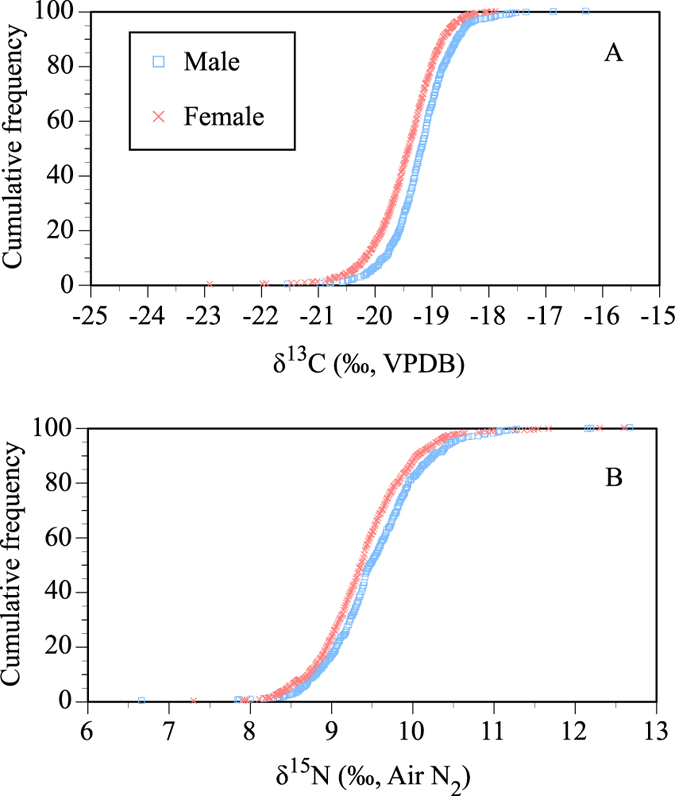
Cumulative frequencies of isotope ratios in hair samples divided by gender: (**A**) carbon and (**B**) nitrogen.

**Figure 4:**
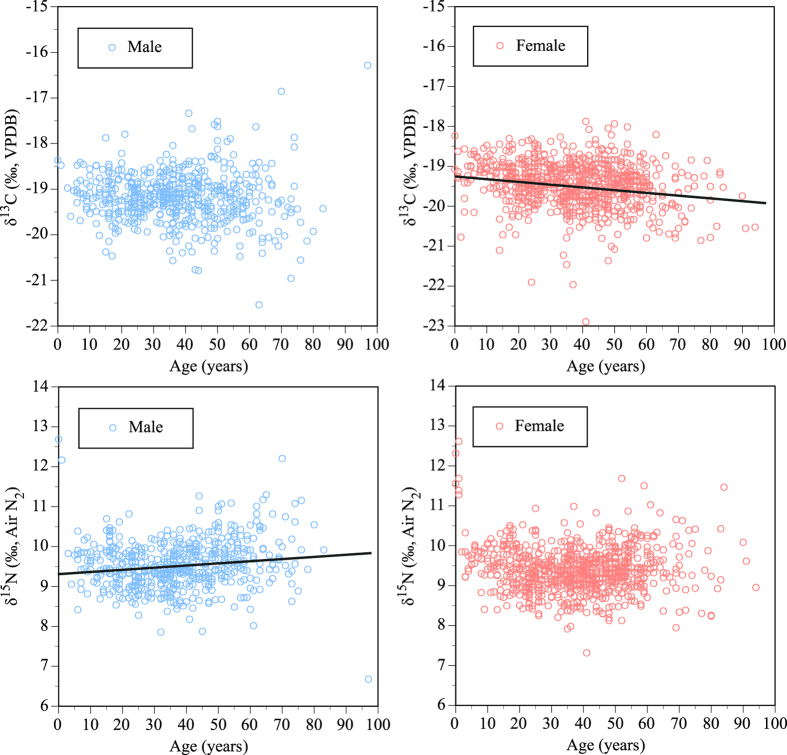
Relationship between carbon and nitrogen isotope ratios of hair samples and ages of individuals divided by sex.

**Figure 5:**
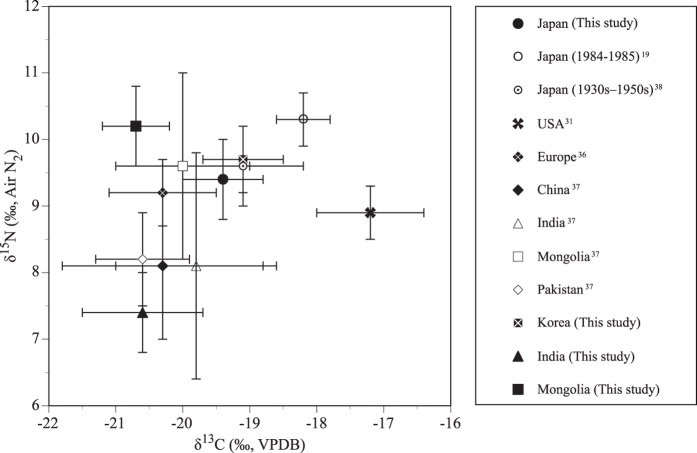
Comparison of carbon and nitrogen isotope ratios between the Japanese and people of other countries.

We collected 1305 hair samples from Japanese during the period of 2007–2010 (Fig. 1, Table 1, Supplementary Table S4). Friends and acquaintances of the authors who lived in each administrative division of Japan assisted with hair sample collection. Because we did not set any restrictions on selecting donors, they include city and rural dwellers. Typically, several scalp hair samples (about 5 cm in length) were collected into a vinyl pack. All donors answered questionnaires about their sex, age, and address (city and administrative division of Japan, not the full address). We also asked persons from whom hair samples were collected how many days they consumed the following ten food items in a week on average: beef, pork, chicken, eggs, marine fish, freshwater fish, tofu (legume product), natto (fermented legume), milk, and cheese. We then recorded the scores (0–7) for the consumption frequency of each food item. For example, a score of 7 for beef means that a donor eats it every day, and a score of 3 for eggs means that the donor eats eggs three days a week on average. For comparative purposes, we collected 32 hair samples from South Korea, 21 samples from India, and 78 samples from Mongolia (Supplementary Table S5).

### Sample preparation and isotope analysis

Hair samples were washed twice in acetone to remove surface contamination and lipids and dried at room temperature. Then, each sample was trimmed with scissors and weighed (~0.5 mg) into a tin capsule.

Food samples bought at a supermarket in Otsu City in 2007–2008 were also analyzed (Table 2). All food samples were dried and then washed in chloroform/methanol (2:1, v/v) to remove lipids. Additional isotopic data on food samples were derived from previous studies^42^,^59^.

The carbon and nitrogen isotope ratios of the hair and food samples were measured with an isotope ratio mass spectrometer (DELTAplus XP, Thermo Fisher Scientific, Inc.) connected to an elemental analyzer (Flash EA, Thermo Fisher Scientific, Inc.). The carbon and nitrogen isotope ratios are expressed as follows:


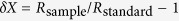


where *X* is ^13^C or ^15^N and *R* is the relative abundance of carbon or nitrogen (*R* = ^13^C/^12^C or ^15^N/^14^N). The isotope ratios were corrected by using multiple secondary standards that were carefully calibrated to international standards^60^. Carbon isotope ratios were reported against the Vienna Pee Dee Belemnite (VPDB) scale, and nitrogen isotope ratios were reported against atmospheric N_2_. The standard errors of the measurements were less than ±0.2‰ based on simultaneous measurements of the working standards.

### Statistical Analyses

The statistical software JMP (SAS Institute) was used to analyze the isotopic data. The *t*-test was used to compare the mean isotopic data between two groups. The statistical significance was evaluated with a *P* value of 0.05. Analysis of variance (ANOVA) was used to compare the mean isotopic data for more than two groups. The relationship between the isotopic values and the ages of individuals was assessed by regression analysis. Analysis of covariance (ANCOVA; model: δ^13^C (or δ^15^N) **~ **sex + age + sex:age) was used to evaluate the relationship between the isotopic values and the effects of sex, age, and the covariance of sex and age. The sex effect is a categorical variable, and the age effect is a continuous variable. Sex:age is the interaction of sex and age effects. Multiple regression analysis was used to detect the significant effects of 10 foods on isotopic values (model: δ^13^C (or δ^15^N) **~ **Food1 + Food2 + … + Food10). The δ^15^N and δ^13^C values are continuous dependent variables. The frequencies of the dietary incorporation per week (0–7 per week) of the 10 foods are continuous explanatory variables.

## Additional Information

**How to cite this article**: Kusaka, S. *et al*. Homogeneous diet of contemporary Japanese inferred from stable isotope ratios of hair. *Sci. Rep.* **6**, 33122; doi: 10.1038/srep33122 (2016).

## Supplementary Material

Supplementary Information

## Figures and Tables

**Table 1:** Isotopic measurement results for the hair samples.

Country		δ^13^C (‰)		δ^15^N (‰)	
	N	Mean	SD	Mean	SD
Japan	1305	−19.4	0.6	9.4	0.6
Male	539	−19.2	0.6	9.5	0.6
Male (0–19)	94	−19.2	0.5	9.6	0.7
Male (20–)	445	−19.2	0.6	9.5	0.6
Female	766	−19.5	0.6	9.4	0.6
Female (0–19)	117	−19.3	0.6	9.7	0.7
Female (20–)	649	−19.5	0.6	9.3	0.5
Eastern Japan	590	−19.4	0.6	9.3	0.6
Western Japan	715	−19.3	0.6	9.5	0.6
Hokkaido	32	−19.7	0.6^a^	9.7	0.6^ab^
Tohoku	135	−19.5	0.6^ab^	9.3	0.6^c^
Kanto	213	−19.5	0.5^ab^	9.3	0.5^bc^
Chubu	210	−19.3	0.6^cd^	9.3	0.6^bc^
Kinki	255	−19.3	0.6^bc^	9.6	0.6^a^
Chugoku	121	−19.2	0.6^d^	9.6	0.6^a^
Shikoku	98	−19.2	0.6^cd^	9.6	0.7^a^
Kyushu	241	−19.4	0.5^abc^	9.5	0.6^abc^
Korea	32	−19.1	0.6	9.7	0.5
India	21	−20.6	0.9	7.4	0.6
Mongolia	78	−20.7	0.5	10.2	0.6

The groups that are not with the same characters are significantly different at the *P* = 0.05 level of Tukey’s HSD test.

**Table 2:** Isotopic measurement results for food samples.

Category	N	δ^15^N	SD	δ^13^C	SD	Country	Ref.
Beef	4	6.9	0.3	−18.9	1.6	Japan	This study
Pork	3	3.9	0.9	−17.6	0.5	Japan	This study
Chicken	4	3.5	0.7	−18.3	1.5	Japan	This study
Egg	3	5.4	1.6	−17.7	2.1	Japan	This study
Marine fish	3	10.4	2.9	−19.9	1.9	Japan	This study
Freshwater fish	1	10.5		−19.4		Japan	This study
Tofu	1	−0.5		−26.2		Japan	This study
Natto	1	0.5		−25.9		North America	This study
Milk	1	5.1		−19.6		Japan	This study
Cheese	3	5.4	0.5	−20.7	3.4	Japan	This study
Rice	38	1.6	0.8	−26.9	0.4	Japan	Suzuki *et al*.^59^
Vegetable (C_3_)	4	3.5	1.1	−25.8	1.6	Japan	Minagawa^19^
Millet (C_4_)	2	1.7	2.8	−10.8	0.4	Japan	Minagawa^19^
Beef (Japan)	66	7.5	0.8	−18.5	1.0	Japan	Nakashita *et al*.^42^
Beef (USA)	20	6.0	0.9	−12.3	1.1	USA	Nakashita *et al*.^42^
Beef (Australia)	53	6.6	0.8	−22.5	1.0	Australia	Nakashita *et al*.^42^
