# Supplementary material for: Homogeneous diet of contemporary Japanese inferred from stable isotope ratios of hair
Source: Sci Rep. 2016 Sep 12;6:33122. doi: 10.1038/srep33122 (PMC5018884; doi:10.1038/srep33122)
Supplement: Supplementary Information [file srep33122-s1.pdf]

Title: Homogeneous diet of contemporary Japanese inferred from stable isotope ratios of hair

Authors: Soichiro Kusaka, Eriko Ishimaru, Fujio Hyodo, Gakuhari Takashi, Minoru Yoneda, Takakazu Yumoto, Ichiro Tayasu

Supplementary Table S1. Isotopic measurement results for Japanese hair divided by prefectural divisions

Prefecture	N	$\delta^{13}C$		$\delta^{15}N$	
		Mean	SD	Mean	SD
Hyogo	25	-19.1	0.6	9.9	0.6
Nagasaki	11	-19.2	0.4	9.9	1.0
Miyazaki	26	-19.3	0.5	9.8	0.4
Kagawa	14	-19.5	0.4	9.8	0.5
Osaka	29	-19.5	0.6	9.7	0.4
Okayama	23	-19.1	0.6	9.7	0.5
Hokkaido	32	-19.7	0.6	9.7	0.6
Tokushima	37	-19.2	0.5	9.7	0.7
Kagoshima	33	-19.3	0.6	9.7	0.8
Tottori	21	-19.2	0.5	9.7	0.8
Wakayama	10	-19.3	0.4	9.6	0.3
Kumamoto	18	-19.3	0.6	9.6	0.5
Mie	31	-19.1	0.5	9.6	0.5
Gunma	27	-19.5	0.6	9.6	0.6
Yamaguchi	23	-19.1	0.6	9.6	0.6
Tochigi	25	-19.4	0.6	9.5	0.4
Aichi	19	-19.2	0.5	9.5	0.5
Kyoto	107	-19.4	0.6	9.5	0.5
Shizuoka	17	-19.1	0.6	9.5	0.5
Hiroshima	24	-19.2	0.5	9.5	0.6
Tokyo	31	-19.4	0.6	9.5	0.6
Ehime	31	-19.2	0.6	9.5	0.7
Nara	43	-19.3	0.8	9.5	0.7
Akita	9	-19.5	0.6	9.4	0.3
Nagano	21	-19.4	0.4	9.4	0.4
Yamanashi	29	-19.2	0.4	9.4	0.4
Gifu	27	-19.3	0.6	9.4	0.5
Ibaraki	10	-19.5	0.3	9.4	0.5
Oita	29	-19.4	0.6	9.4	0.5
Shimane	30	-19.2	0.6	9.4	0.5
Aomori	39	-19.3	0.7	9.4	0.7
Ishikawa	28	-19.4	0.6	9.4	0.8
Iwate	33	-19.4	0.5	9.3	0.4
Fukuoka	56	-19.4	0.6	9.3	0.5
Miyagi	24	-19.6	0.5	9.3	0.5
Shiga	10	-19.6	0.5	9.3	0.5
Saga	39	-19.5	0.5	9.3	0.6
Yamagata	16	-19.5	0.4	9.3	0.9
Chiba	42	-19.4	0.4	9.2	0.4
Okinawa	29	-19.4	0.6	9.2	0.4
Kanagawa	18	-19.5	0.4	9.2	0.5
Saitama	60	-19.6	0.5	9.2	0.5
Niigata	30	-19.3	0.6	9.2	0.6
Toyama	21	-19.2	0.7	9.2	0.6
Kochi	16	-18.9	0.8	9.2	0.9
Fukui	18	-19.1	0.6	9.1	0.5
Fukushima	14	-19.6	0.5	9.0	0.5

Title: Homogeneous diet of contemporary Japanese inferred from stable isotope ratios of hair

Authors: Soichiro Kusaka, Eriko Ishimaru, Fujio Hyodo, Gakuhari Takashi, Minoru Yoneda, Takakazu Yumoto, Ichiro Tayasu

Supplementary Table S2. Statistics for the multiple regression analysis on the isotope ratios and the results of the questionnaire on dietary habits

	$\delta^{13}C$ (‰)				$\delta^{15}N$ (‰)			
	Estimates	SE	*t*	*P*	Estimates	SE	*t*	*P*
Intercept	-19.536	0.053	-369.8	<0.0001*	9.280	0.053	175.9	<0.0001*
Beef	0.079	0.014	5.7	<0.0001*	0.053	0.014	3.8	0.0001*
Pork	0.013	0.012	1.1	0.2779	-0.019	0.012	-1.5	0.1251
Chicken	0.024	0.014	1.7	0.086	-0.021	0.014	-1.5	0.1308
Egg	0.016	0.008	2.1	0.0364*	-0.002	0.008	-0.3	0.7549
Marine fish	0.025	0.010	2.5	0.0112*	0.121	0.010	12.2	<0.0001*
Freshwater fish	-0.022	0.021	-1.1	0.2802	0.002	0.021	0.1	0.9239
Tofu	-0.029	0.009	-3.3	0.0011*	-0.025	0.009	-2.8	0.0052*
Natto	-0.024	0.009	-2.8	0.0051*	-0.035	0.009	-4.1	<0.0001*
Milk	0.011	0.006	1.9	0.0623	0.001	0.006	0.2	0.8747
Cheese	-0.007	0.011	-0.6	0.5368	-0.009	0.011	-0.8	0.4177

* Statistically significant at the level of $P < 0.05$

Title: Homogeneous diet of contemporary Japanese inferred from stable isotope ratios of hair

Authors: Soichiro Kusaka, Eriko Ishimaru, Fujio Hyodo, Gakuhari Takashi, Minoru Yoneda, Takakazu Yumoto, Ichiro Tayasu

Supplementary Table S3. Comparison of the isotope measurement results on hair samples

Country	Description	N	$\delta^{13}C$ (‰)			$\delta^{15}N$ (‰)			Ref.
			Mean		SD	Mean		SD	
Japan (2007–2010)	Omnivore	1305	-19.4	±	0.6	9.4	±	0.6	This study
Japan (1984–1985)	Omnivore	42	-18.2	±	0.4	10.3	±	0.4	Minagawa et al.[19]
Japan (Ainu, 1930s–1950s)	unknown	-	-19.1	±	0.9	9.6	±	0.6	Bowen et al.[38]
South Korea	Omnivore	32	-19.1	±	0.6	9.7	±	0.5	This study
India	Vegan	21	-20.6	±	0.9	7.4	±	0.6	This study
Mongolia	Omnivore	78	-20.7	±	0.5	10.2	±	0.6	This study
USA(2003–2007)	Omnivore	234	-17.2	±	0.8	8.9	±	0.4	Valenzuela et al.[31]
Europe	Omnivore	126	-20.3	±	0.8	9.2	±	0.5	Valenzuela et al.[36]
China	unknown	73	-20.3	±	1.5	8.1	±	1.1	Thompson et al.[37]
India	unknown	38	-19.8	±	1.2	8.1	±	1.7	Thompson et al.[37]
Mongolia	unknown	20	-20.0	±	1.0	9.6	±	1.4	Thompson et al.[37]
Pakistan	unknown	11	-20.6	±	0.7	8.2	±	0.7	Thompson et al.[37]

Title: Homogeneous diet of contemporary Japanese inferred from stable isotope ratios of hair
Authors: Soichiro Kusaka, Eriko Ishimaru, Fujio Hyodo, Gakuhari Takashi, Minoru Yoneda, Takakazu Yumoto, Ichiro Tayasu

Supplementary Table S4. Isotopic measurement results for contemporary Japanese hair

No.	Age	Sex	$\delta^{13}C$	$\delta^{15}N$	Beef	Pork	Chicken	Egg	Marine fish	Fresh water fish	Tofu	Natto	Milk	Cheese	Prefecture	Region	East/West
1	22	Male	-19.8	9.9	0	2	3	1	4	0	1	3	6	2	Kyoto	Kinki	West
2	35	Female	-19.7	9.1	1	2	1	6	2	0	5	0	7	4	Shiga	Kinki	West
3	45	Female	-19.8	9.1	0	2	1	1	1	2	2	4	7	3	Gunma	Kanto	East
4	9	Male	-19.5	9.4	1	1	1	2	3	0	3	1	6	1	Fukuoka	Kyushu	West
5	40	Female	-19.9	9.3	2	3	2	2	3	0	3	0	4	0	Fukuoka	Kyushu	West
6	69	Male	-19.3	9.7	0	1	1	1	1	6	0	2	2	4	Kyoto	Kinki	West
7	20	Male	-19.3	8.7	3	3	3	5	2	2	3	1	3	1	Kyoto	Kinki	West
8	22	Male	-19.4	9.3	2	5	3	2	2	0	2	1	7	3	Kyoto	Kinki	West
9	41	Female	-20.2	9.1	1	2	2	4	2	0	3	1	7	3	Kyoto	Kinki	West
10	52	Female	-19.5	8.8	2	2	3	3	1	0	3	2	3	2	Shiga	Kinki	West
11	53	Female	-19.8	9.2	1	1	1	1	2	1	5	0	6	0	Kyoto	Kinki	West
12	10	Female	-19.1	10.0	1	2	3	2	0	0	0	0	1	0	Kyoto	Kinki	West
13	34	Female	-19.9	8.8	0	2	2	6	5	0	6	0	7	6	Kyoto	Kinki	West
14	73	Male	-19.2	10.2	0	3	1	0	5	0	0	3	7	0	Osaka	Kinki	West
15	33	Male	-19.1	9.3	1	2	3	7	3	0	2	5	7	5	Nara	Kinki	West
16	33	Female	-19.3	9.1	1	1	2	3	5	0	1	3	0	1	Nara	Kinki	West
17	58	Female	-20.0	8.9	1	5	5	7	4	1	5	5	3	3	Osaka	Kinki	West
18	7	Male	-19.7	8.9	1	5	2	1	7	0	3	7	7	4	Kanagawa	Kanto	East
19	44	Male	-18.9	9.2	1	2	2	7	3	0	7	3	1	4	Nara	Kinki	West
20	13	Male	-20.0	9.3	2	2	2	7	3	0	3	0	5	5	Osaka	Kinki	West
21	8	Male	-19.1	9.8	3	3	3	2	5	0	2	4	7	3	Nara	Kinki	West
22	7	Female	-18.8	9.8	3	3	3	3	3	0	3	3	7	2	Nara	Kinki	West
23	6	Male	-18.5	10.4	0	2	0	0	2	0	0	4	0	7	Nara	Kinki	West
24	41	Male	-18.5	9.9	2	2	1	3	3	0	2	0	7	2	Nara	Kinki	West
25	32	Female	-19.8	9.0	3	3	3	2	2	2	2	0	0	0	Kyoto	Kinki	West
26	29	Female	-19.4	9.7	2	2	2	4	3	0	7	2	2	3	Hiroshima	Chugoku	West
27	41	Male	-18.9	9.8	1	2	1	2	3	0	4	2	0	0	Tokyo	Kanto	East
28	20	Female	-19.6	9.7	2	2	0	7	4	0	3	0	7	0	Kyoto	Kinki	West
29	21	Female	-19.5	9.5	2	1	1	3	3	1	1	2	0	0	Kyoto	Kinki	West
30	10	Male	-19.0	9.6	1	3	1	3	3	0	7	7	7	5	Kyoto	Kinki	West
31	37	Female	-20.1	9.0	0	2	2	2	1	1	0	1	7	2	Shiga	Kinki	West
32	26	Male	-19.5	9.9	2	2	2	4	3	1	2	2	5	5	Kyoto	Kinki	West
33	47	Female	-19.0	9.5	1	2	2	3	2	0	3	1	7	1	Hyogo	Kinki	West
34	16	Male	-19.5	10.1	2	5	2	6	2	0	4	1	7	1	Osaka	Kinki	West
35	13	Female	-19.2	9.9	0	2	3	7	4	0	3	2	5	1	Fukuoka	Kyushu	West
36	49	Female	-19.2	9.3	1	2	2	1	2	0	3	0	5	0	Kyoto	Kinki	West
37	15	Male	-18.8	9.8	2	2	2	4	3	2	4	1	7	2	Aichi	Chubu	East
38	18	Male	-19.2	9.4	2	3	3	5	3	1	7	6	7	0	Aichi	Chubu	East
39	17	Male	-18.7	10.4	0	3	1	7	7	1	3	1	1	0	Aichi	Chubu	East
40	27	Female	-19.3	8.6	1	0	5	0	2	0	3	0	3	0	Mie	Kinki	West
41	24	Female	-19.4	9.3	1	1	1	2	3	1	1	3	3	0	Mie	Kinki	West
42	4	Male	-19.6	9.0	0	1	2	2	2	0	2	2	0	1	Kyoto	Kinki	West
43	17	Female	-18.3	10.2	1	2	2	5	2	0	1	0	1	0	Mie	Kinki	West
44	32	Female	-19.5	9.4	0	3	2	2	2	0	2	2	0	1	Kyoto	Kinki	West
45	17	Male	-18.9	9.7	3	3	0	7	2	0	1	2	7	0	Aichi	Chubu	East
46	16	Male	-18.9	9.9	0	7	2	7	2	2	0	1	7	3	Aichi	Chubu	East
47	14	Female	-21.1	8.7	0	0	2	4	6	0	3	1	0	5	Nara	Kinki	West
48	41	Female	-22.9	7.3	0	0	0	0	1	1	4	1	0	0	Nara	Kinki	West
49	15	Female	-18.8	10.0	3	3	3	7	6	2	4	0	3	1	Mie	Kinki	West
50	15	Female	-19.0	9.6	3	2	1	7	1	0	6	1	3	0	Mie	Kinki	West
51	16	Female	-19.6	9.4	1	1	1	3	2	1	4	7	3	3	Mie	Kinki	West
52	44	Female	-20.2	9.0	1	2	2	3	3	1	6	2	2	1	Fukuoka	Kyushu	West
53	17	Female	-19.9	10.2	1	1	2	7	3	3	1	1	3	1	Fukuoka	Kyushu	West
54	17	Female	-19.1	9.5	1	1	1	5	2	1	2	3	3	1	Fukuoka	Kyushu	West
55	18	Male	-19.3	9.9	2	1	1	4	3	1	0	0	1	0	Miyagi	Tohoku	East
56	17	Male	-19.1	10.0	3	3	3	7	1	0	0	3	7	0	Hyogo	Kinki	West
57	17	Female	-18.7	10.5	0	2	2	3	5	0	3	1	7	0	Chiba	Kanto	East
58	16	Female	-19.4	9.4	1	2	1	1	3	0	3	0	7	1	Hyogo	Kinki	West
59	22	Female	-19.9	10.0	1	3	0	7	1	0	2	0	0	0	Mie	Kinki	West
60	16	Female	-19.2	9.8	2	1	2	7	4	2	5	0	7	5	Nara	Kinki	West
61	15	Female	-19.7	10.1	1	3	3	7	2	0	1	2	2	0	Nara	Kinki	West
62	16	Male	-20.2	9.4	5	4	4	4	2	1	1	0	1	2	Osaka	Kinki	West
63	15	Female	-18.9	10.0	2	2	1	5	2	0	2	0	7	0	Osaka	Kinki	West
64	16	Female	-19.9	9.2	2	2	2	5	2	0	5	2	7	2	Osaka	Kinki	West
65	44	Female	-20.1	8.9	0	2	2	1	1	1	4	4	0	2	Tochigi	Kanto	East
66	15	Female	-20.7	9.1	0	3	3	3	2	2	3	3	1	3	Tochigi	Kanto	East
67	16	Female	-19.4	10.0	3	0	2	5	5	2	1	0	7	2	Osaka	Kinki	West
68	16	Female	-19.3	9.9	3	1	1	6	3	3	0	1	7	0	Osaka	Kinki	West
69	16	Male	-18.5	9.9	5	0	3	4	3	3	2	3	7	3	Osaka	Kinki	West

70	15	Male	-19.3	9.9	3	3	2	7	3	3	7	0	7	0	Osaka	Kinki	West
71	16	Male	-19.2	10.5	2	1	2	3	1	0	2	1	2	0	Hyogo	Kinki	West
72	17	Female	-19.1	10.2	1	1	1	4	1	1	3	1	6	1	Aichi	Chubu	East
73	17	Female	-19.3	9.6	2	1	1	3	0	0	1	1	4	0	Osaka	Kinki	West
74	17	Female	-18.5	10.0	0	3	0	4	1	0	1	0	7	0	Aichi	Chubu	East
75	22	Male	-19.4	10.8	5	3	1	4	4	0	3	0	6	3	Nara	Kinki	West
76	15	Male	-17.9	10.7	4	2	3	4	5	0	3	0	3	1	Nara	Kinki	West
77	48	Female	-19.3	10.4	4	1	1	5	5	0	3	0	1	4	Nara	Kinki	West
78	43	Female	-20.0	9.5	1	5	2	0	7	1	7	5	5	0	Tokyo	Kanto	East
79	9	Male	-19.2	9.8	1	5	2	0	7	1	7	6	7	0	Tokyo	Kanto	East
80	17	Male	-19.1	9.8	2	2	2	3	5	0	2	1	7	2	Nara	Kinki	West
81	16	Male	-19.2	9.7	4	3	2	4	1	1	1	0	7	2	Osaka	Kinki	West
82	18	Male	-19.6	9.6	5	1	0	3	0	0	0	0	0	0	Niigata	Chubu	East
83	18	Male	-19.9	9.2	3	2	2	4	4	2	1	3	4	1	Niigata	Chubu	East
84	18	Male	-19.3	9.2	0	1	5	6	3	0	7	7	2	0	Niigata	Chubu	East
85	17	Male	-19.8	10.0	1	4	2	7	2	1	5	0	7	0	Hokkaido	Hokkaido	East
86	18	Female	-19.2	9.4	3	3	3	5	3	3	6	6	7	5	Aichi	Chubu	East
87	42	Female	-19.6	9.6	2	3	3	3	3	0	3	3	4	1	Hiroshima	Chugoku	West
88	15	Male	-19.0	10.4	2	3	3	4	3	1	3	1	5	0	Hiroshima	Chugoku	West
89	16	Male	-18.5	10.4	3	3	3	6	5	1	5	0	7	2	Osaka	Kinki	West
90	51	Female	-18.9	9.1	1	3	3	5	2	0	7	4	0	0	Kyoto	Kinki	West
91	59	Male	-19.4	10.3	0	2	2	7	4	0	2	2	3	0	Nagano	Chubu	East
92	52	Male	-19.4	9.6	1	3	3	3	6	0	6	3	4	0	Kyoto	Kinki	West
93	16	Female	-19.0	10.4	2	4	3	5	6	1	7	7	7	0	Osaka	Kinki	West
94	16	Male	-18.6	10.3	2	2	2	6	3	1	6	0	7	1	Osaka	Kinki	West
95	20	Female	-19.4	8.9	1	1	1	3	0	0	3	0	3	0	Toyama	Chubu	East
96	18	Female	-19.9	10.3	0	3	3	7	3	3	0	7	3	3	Wakayama	Kinki	West
97	19	Male	-18.8	10.6	5	4	5	7	6	1	4	1	4	1	Tottori	Chugoku	West
98	57	Female	-19.3	9.7	0	3	3	3	7	3	3	7	7	0	Wakayama	Kinki	West
99	13	Female	-19.4	8.4	1	2	2	3	0	0	0	1	7	3	Kanagawa	Kanto	East
100	16	Male	-19.6	9.3	1	2	1	3	2	2	1	4	2	0	Kanagawa	Kanto	East
101	56	Female	-19.4	10.4	2	1	2	7	3	1	3	3	3	7	Kyoto	Kinki	West
102	13	Female	-19.0	9.6	5	2	2	5	2	1	1	1	1	0	Shiga	Kinki	West
103	55	Female	-19.5	9.8	2	2	2	5	3	0	6	1	7	2	Kyoto	Kinki	West
104	40	Female	-20.4	9.0	1	2	1	7	3	0	3	5	7	1	Osaka	Kinki	West
105	13	Female	-19.7	9.5	4	2	1	7	3	0	3	3	3	6	Osaka	Kinki	West
106	47	Female	-19.6	9.2	3	3	3	1	1	1	3	0	0	1	Kyoto	Kinki	West
107	46	Female	-20.2	9.4	1	3	3	1	2	0	4	0	0	2	Kyoto	Kinki	West
108	58	Female	-19.3	8.5	1	2	2	2	2	1	3	3	7	1	Kyoto	Kinki	West
109	56	Female	-19.5	10.1	1	3	2	0	2	2	7	4	7	7	Kyoto	Kinki	West
110	48	Female	-19.0	9.7	1	5	2	5	5	1	6	1	2	3	Tokyo	Kanto	East
111	53	Male	-19.7	9.4	2	2	1	4	2	0	4	2	7	5	Tokyo	Kanto	East
112	9	Female	-18.9	9.3	2	1	1	3	1	1	2	1	5	0	Kyoto	Kinki	West
113	39	Female	-19.9	8.4	1	1	1	1	3	1	3	3	4	1	Kyoto	Kinki	West
114	42	Female	-19.3	9.7	5	0	0	0	1	0	2	0	7	0	Shiga	Kinki	West
115	30	Female	-19.8	9.1	0	3	0	2	3	3	2	0	0	1	Kyoto	Kinki	West
116	15	Male	-19.1	9.4	2	4	1	5	1	0	3	5	7	0	Aichi	Chubu	East
117	10	Male	-18.7	9.9	7	4	0	3	4	0	4	6	7	3	Fukuoka	Kyushu	West
118	52	Female	-20.2	9.6	0	1	1	1	4	0	4	0	7	1	Tokyo	Kanto	East
119	12	Male	-18.5	9.7	1	2	1	0	5	0	1	0	7	1	Niigata	Chubu	East
120	14	Male	-19.4	8.9	0	1	0	1	1	0	1	3	6	0	Saitama	Kanto	East
121	38	Female	-19.1	10.2	2	2	2	3	3	3	3	3	7	3	Osaka	Kinki	West
122	72	Female	-20.4	8.4	3	3	3	3	3	0	3	0	3	0	Shiga	Kinki	West
123	16	Female	-19.6	8.5	1	1	2	4	2	0	7	7	2	1	Kanagawa	Kanto	East
124	48	Female	-19.4	10.4	1	1	1	3	3	0	3	7	7	0	Kyoto	Kinki	West
125	9	Male	-19.4	10.2	2	1	0	3	4	0	4	6	1	1	Hyogo	Kinki	West
126	47	Male	-18.8	10.4	2	2	1	3	2	0	2	4	5	1	Hyogo	Kinki	West
127	20	Female	-19.6	9.9	4	0	0	2	4	0	3	4	3	0	Osaka	Kinki	West
128	20	Female	-18.9	9.7	2	1	1	2	2	0	1	2	7	4	Kyoto	Kinki	West
129	82	Female	-20.5	8.9	0	1	1	1	4	0	4	6	7	0	Kyoto	Kinki	West
130	13	Male	-19.2	9.6	3	2	4	5	6	5	7	7	4	6	Tokyo	Kanto	East
131	54	Female	-19.7	9.7	3	3	0	0	3	0	4	0	0	1	Tokyo	Kanto	East
132	20	Male	-18.4	9.3	2	2	0	1	0	0	2	0	7	5	Kyoto	Kinki	West
133	42	Female	-18.5	9.6	3	3	0	3	3	0	0	3	7	3	Saitama	Kanto	East
134	45	Male	-19.0	9.9	3	0	0	3	3	0	0	3	7	0	Saitama	Kanto	East
135	39	Female	-19.3	9.0	1	1	1	3	2	0	2	1	7	5	Hyogo	Kinki	West
136	42	Male	-18.7	10.2	1	2	2	5	2	0	2	1	0	2	Hyogo	Kinki	West
137	24	Female	-19.3	9.2	0	5	1	4	1	0	1	1	5	1	Kyoto	Kinki	West
138	42	Female	-19.0	9.9	1	3	1	5	4	0	3	0	7	3	Kyoto	Kinki	West
139	44	Male	-19.8	10.4	1	3	1	5	4	0	3	0	7	3	Kyoto	Kinki	West
140	9	Female	-18.6	9.8	1	3	1	5	4	0	3	1	7	3	Kyoto	Kinki	West
141	16	Male	-19.3	10.4	3	3	3	2	3	3	0	0	7	0	Kyoto	Kinki	West
142	72	Female	-20.4	8.9	1	2	4	4	3	0	3	0	7	1	Kyoto	Kinki	West
143	43	Female	-19.0	9.2	1	2	4	4	2	0	3	2	7	2	Kyoto	Kinki	West
144	20	Female	-19.8	9.4	0	0	1	0	1	0	3	3	2	1	Kyoto	Kinki	West
145	31	Male	-19.7	9.7	4	3	5	5	7	0	5	2	0	0	Kyoto	Kinki	West

146	39	Female	-20.3	9.6	0	3	2	4	7	0	4	5	2	0	Tokyo	Kanto	East
147	44	Male	-19.3	11.3	2	0	1	3	5	0	2	3	1	0	Kyoto	Kinki	West
148	55	Male	-19.4	9.6	1	0	0	1	1	0	6	2	7	0	Osaka	Kinki	West
149	34	Female	-19.2	9.4	1	3	1	3	2	0	3	2	7	2	Kyoto	Kinki	West
150	60	Female	-19.4	10.2	0	2	2	1	4	0	5	1	7	0	Kyoto	Kinki	West
151	57	Female	-19.4	9.1	0	3	0	3	3	0	2	2	1	0	Kyoto	Kinki	West
152	23	Male	-18.9	10.1	2	2	2	6	1	0	5	3	7	1	Shiga	Kinki	West
153	50	Male	-18.1	9.4	1	1	1	7	2	0	1	0	7	4	Kyoto	Kinki	West
154	55	Female	-19.2	8.7	0	1	0	3	7	0	6	5	0	0	Osaka	Kinki	West
155	46	Female	-20.0	9.3	0	1	2	1	0	0	1	1	0	3	Osaka	Kinki	West
156	12	Female	-18.6	10.1	2	1	1	6	1	1	5	0	7	1	Kyoto	Kinki	West
157	8	Female	-18.9	9.7	1	1	1	7	1	1	5	3	7	3	Kyoto	Kinki	West
158	46	Male	-18.6	9.1	2	3	3	5	2	1	2	0	3	1	Kyoto	Kinki	West
159	64	Male	-20.1	9.9	1	3	4	7	3	2	1	4	2	2	Osaka	Kinki	West
160	44	Female	-19.8	9.7	1	2	1	3	3	1	1	0	7	0	Hyogo	Kinki	West
161	55	Male	-18.9	10.5	1	2	1	2	7	0	2	3	0	2	Tokyo	Kanto	East
162	55	Male	-18.9	10.3	1	1	1	3	2	0	1	2	3	0	Kanagawa	Kanto	East
163	37	Female	-19.7	9.9	1	2	2	7	3	0	0	0	0	5	Kyoto	Kinki	West
164	33	Male	-20.1	9.2	1	5	3	3	2	0	5	1	5	0	Kyoto	Kinki	West
165	56	Female	-19.3	10.2	0	2	1	3	4	0	2	0	7	0	Kyoto	Kinki	West
166	45	Female	-20.6	9.6	1	1	2	4	2	1	4	1	4	1	Gifu	Chubu	East
167	23	Female	-19.3	9.7	0	3	3	7	3	0	3	3	3	3	Aichi	Chubu	East
168	24	Male	-19.1	10.0	0	1	3	3	4	0	3	0	2	0	Aichi	Chubu	East
169	22	Female	-19.7	9.4	0	2	2	2	1	0	3	3	5	0	Kyoto	Kinki	West
170	38	Male	-19.8	9.0	2	2	1	1	1	0	1	1	0	0	Tokyo	Kanto	East
171	48	Female	-21.4	8.1	0	0	1	0	2	0	6	1	1	1	Kyoto	Kinki	West
172	23	Female	-19.3	8.4	0	2	2	1	2	0	2	1	4	1	Nara	Kinki	West
173	50	Female	-20.3	9.5	1	3	1	2	4	0	5	1	7	1	Saitama	Kanto	East
174	18	Female	-19.1	10.4	1	1	1	7	2	2	4	5	7	2	Kyoto	Kinki	West
175	12	Male	-19.5	9.2	1	0	0	0	0	0	0	7	7	7	Kyoto	Kinki	West
176	21	Female	-18.4	9.2	0	5	3	7	3	0	4	3	5	1	Osaka	Kinki	West
177	28	Male	-19.5	8.8	2	4	3	5	1	1	2	0	1	1	Kyoto	Kinki	West
178	19	Female	-20.1	9.0	1	1	2	0	2	0	0	0	0	0	Kyoto	Kinki	West
179	69	Female	-19.3	10.2	0	0	1	2	5	0	2	2	7	7	Hyogo	Kinki	West
180	45	Male	-19.4	9.2	0	1	1	3	2	0	1	2	1	1	Chiba	Kanto	East
181	36	Male	-19.8	10.1	0	3	1	7	4	0	1	0	0	0	Chiba	Kanto	East
182	32	Male	-19.6	9.2	1	2	1	1	4	0	4	4	5	1	Chiba	Kanto	East
183	24	Male	-19.8	9.9	2	2	2	1	2	0	0	0	3	0	Kyoto	Kinki	West
184	23	Female	-19.8	8.8	1	6	2	6	1	1	2	2	7	0	Kyoto	Kinki	West
185	51	Female	-19.6	9.2	0	3	0	7	4	0	3	5	7	0	Kyoto	Kinki	West
186	60	Female	-19.8	9.8	0	2	2	2	2	2	4	2	3	1	Osaka	Kinki	West
187	60	Male	-19.1	9.2	2	3	3	3	2	0	3	3	5	3	Kyoto	Kinki	West
188	36	Female	-20.0	9.1	0	1	4	0	3	0	0	0	3	0	Osaka	Kinki	West
189	33	Female	-20.0	9.4	2	3	3	3	2	0	2	2	7	1	Kyoto	Kinki	West
190	27	Male	-19.5	9.4	1	1	1	1	7	0	7	7	7	7	Tokyo	Kanto	East
191	50	Female	-19.5	9.7	2	2	2	1	3	0	1	0	7	0	Osaka	Kinki	West
192	37	Male	-19.9	9.0	3	2	3	5	4	0	7	0	7	0	Yamaguchi	Chugoku	West
193	19	Male	-19.5	9.1	1	2	2	7	3	0	7	1	7	0	Shiga	Kinki	West
194	18	Male	-19.0	9.9	1	4	4	4	3	1	1	0	0	1	Kyoto	Kinki	West
195	73	Male	-21.0	9.4	1	1	0	7	7	0	2	0	1	1	Osaka	Kinki	West
196	74	Male	-19.0	9.6	1	2	3	4	3	0	5	2	5	1	Kyoto	Kinki	West
197	36	Male	-19.2	9.8	1	1	1	2	2	0	2	3	0	0	Kyoto	Kinki	West
198	61	Female	-19.6	11.0	0	3	3	7	3	0	3	0	7	3	Hyogo	Kinki	West
199	60	Female	-20.8	8.7	0	0	3	3	0	0	7	3	0	7	Nara	Kinki	West
200	49	Male	-19.1	9.9	3	0	4	1	2	0	2	0	7	0	Kyoto	Kinki	West
201	43	Male	-19.2	10.0	0	3	2	3	7	0	0	0	7	0	Hokkaido	Hokkaido	East
202	63	Male	-19.5	9.6	1	2	3	3	3	0	1	0	0	0	Kyoto	Kinki	West
203	62	Female	-19.8	9.4	1	2	3	3	3	0	2	0	4	1	Kyoto	Kinki	West
204	63	Female	-18.2	10.3	2	0	2	5	5	0	4	2	3	0	Kyoto	Kinki	West
205	63	Female	-19.3	9.6	1	2	2	3	3	0	2	3	6	6	Kyoto	Kinki	West
206	78	Female	-19.3	9.4	2	3	2	3	7	1	7	0	7	1	Kyoto	Kinki	West
207	63	Male	-18.4	10.5	1	2	1	3	3	1	2	1	0	1	Kyoto	Kinki	West
208	34	Male	-19.2	9.0	1	3	3	3	3	0	5	0	5	0	Kyoto	Kinki	West
209	80	Female	-19.9	9.2	0	1	0	6	3	0	4	3	0	0	Kyoto	Kinki	West
210	19	Male	-19.9	8.9	0	3	3	0	1	0	0	6	0	0	Kyoto	Kinki	West
211	10	Male	-19.6	9.6	3	0	2	7	3	0	3	3	3	1	Fukui	Chubu	East
212	37	Female	-19.1	9.5	0	5	3	4	2	0	1	1	7	4	Kyoto	Kinki	West
213	71	Female	-20.3	9.3	0	2	1	1	4	0	7	0	0	1	Shiga	Kinki	West
214	17	Female	-18.9	9.3	1	2	1	1	2	0	1	0	5	0	Kyoto	Kinki	West
215	43	Female	-19.0	9.5	1	2	1	1	2	0	3	1	7	0	Kyoto	Kinki	West
216	45	Female	-18.8	9.5	2	3	2	5	3	1	2	1	3	1	Kyoto	Kinki	West
217	41	Male	-19.5	8.2	1	1	1	2	1	0	1	2	0	1	Fukuoka	Kyushu	West
218	24	Female	-21.9	8.5	0	3	3	3	7	0	7	0	0	0	Fukuoka	Kyushu	West
219	22	Female	-19.1	8.8	1	2	1	0	2	0	1	0	0	0	Fukuoka	Kyushu	West
220	54	Male	-19.1	9.1	0	1	1	7	5	0	7	2	1	0	Fukuoka	Kyushu	West
221	36	Female	-19.0	8.0	0	2	3	5	1	1	1	0	5	1	Fukuoka	Kyushu	West

222	24	Male	-19.7	8.7	1	1	1	1	1	0	1	0	0	0	Fukuoka	Kyushu	West
223	23	Male	-18.9	9.8	2	2	2	3	4	2	1	0	0	1	Miyazaki	Kyushu	West
224	27	Female	-19.5	9.1	1	3	1	4	1	0	1	1	1	0	Fukuoka	Kyushu	West
225	35	Male	-18.6	8.7	4	1	1	5	2	0	3	6	3	0	Fukuoka	Kyushu	West
226	43	Female	-19.5	9.6	1	4	5	3	2	1	2	3	0	1	Fukuoka	Kyushu	West
227	42	Female	-18.7	9.3	1	3	2	1	3	0	6	0	0	1	Fukuoka	Kyushu	West
228	48	Male	-19.4	9.7	1	3	2	3	3	0	5	0	0	0	Fukuoka	Kyushu	West
229	25	Male	-19.7	8.8	5	3	3	2	3	3	2	1	0	0	Fukuoka	Kyushu	West
230	32	Male	-19.0	9.0	0	2	1	5	0	0	2	0	3	0	Fukuoka	Kyushu	West
231	36	Male	-19.1	8.9	0	2	3	6	1	0	2	0	0	0	Fukuoka	Kyushu	West
232	32	Female	-19.0	8.7	1	3	3	5	4	0	2	0	7	2	Fukuoka	Kyushu	West
233	36	Female	-19.6	9.4	1	2	2	1	3	0	4	3	0	0	Fukuoka	Kyushu	West
234	21	Male	-17.8	9.8	7	7	3	1	1	0	1	0	3	1	Hiroshima	Chugoku	West
235	20	Male	-18.2	9.3	3	3	3	2	2	0	0	0	3	0	Hiroshima	Chugoku	West
236	28	Male	-19.1	9.4	1	4	2	4	3	0	3	1	0	0	Hiroshima	Chugoku	West
237	22	Male	-19.5	9.0	2	3	1	1	7	0	2	2	4	2	Hiroshima	Chugoku	West
238	20	Female	-20.3	8.8	1	1	2	3	0	0	1	1	3	3	Hiroshima	Chugoku	West
239	19	Female	-19.4	9.7	3	3	3	2	0	0	0	1	0	0	Hiroshima	Chugoku	West
240	21	Male	-19.2	9.6	2	3	4	6	5	0	1	1	1	3	Hiroshima	Chugoku	West
241	23	Female	-19.6	9.0	2	2	2	2	2	0	0	3	7	1	Hiroshima	Chugoku	West
242	52	Male	-18.9	9.7	1	0	0	1	1	0	0	7	1	0	Kumamoto	Kyushu	West
243	26	Male	-19.1	9.8	2	2	3	3	1	2	1	1	5	1	Oita	Kyushu	West
244	43	Male	-20.8	8.8	0	0	0	1	1	1	3	3	0	0	Gifu	Chubu	East
245	47	Female	-20.3	8.8	2	2	1	0	3	0	4	1	0	1	Chiba	Kanto	East
246	57	Male	-18.8	10.4	1	1	3	1	7	0	3	1	0	0	Kumamoto	Kyushu	West
247	54	Male	-19.2	10.5	0	2	0	7	1	0	3	0	0	0	Nagasaki	Kyushu	West
248	59	Female	-19.7	9.0	1	3	5	7	7	5	7	5	2	2	Oita	Kyushu	West
249	50	Male	-19.7	9.7	3	2	1	1	4	0	4	1	3	1	Kumamoto	Kyushu	West
250	54	Male	-19.3	9.6	1	1	1	0	2	0	1	4	4	0	Shimane	Chugoku	West
251	27	Male	-19.4	9.4	0	5	2	4	0	0	1	0	1	5	Kumamoto	Kyushu	West
252	28	Female	-19.6	8.9	2	3	1	3	4	0	0	3	3	0	Hyogo	Kinki	West
253	24	Female	-19.0	9.2	1	1	1	1	1	0	0	0	7	1	Kumamoto	Kyushu	West
254	38	Female	-19.0	9.4	1	2	0	0	2	0	3	3	7	0	Oita	Kyushu	West
255	40	Male	-19.4	9.1	3	3	3	3	3	0	2	1	7	3	Kyoto	Kinki	West
256	70	Male	-19.3	10.0	1	1	1	2	3	1	3	1	6	1	Kagoshima	Kyushu	West
257	28	Male	-20.0	8.7	3	3	3	3	0	0	2	0	1	1	Oita	Kyushu	West
258	25	Male	-19.6	8.9	2	1	2	0	3	0	3	1	6	1	Oita	Kyushu	West
259	23	Male	-19.0	9.0	1	3	6	7	3	0	3	3	7	3	Oita	Kyushu	West
260	19	Male	-18.8	8.9	3	0	5	0	0	0	0	3	7	0	Oita	Kyushu	West
261	66	Male	-19.3	9.4	1	1	2	5	1	0	5	5	5	2	Kumamoto	Kyushu	West
262	49	Male	-19.1	10.1	1	1	1	1	7	1	5	5	3	1	Miyazaki	Kyushu	West
263	57	Male	-20.3	9.1	0	1	2	2	4	0	6	3	7	1	Yamaguchi	Chugoku	West
264	54	Male	-19.6	10.1	1	2	1	2	2	0	2	0	0	0	Oita	Kyushu	West
265	61	Male	-20.0	8.0	0	0	0	0	3	3	7	7	7	0	Kanagawa	Kanto	East
266	53	Male	-19.2	10.2	1	1	2	7	6	1	5	2	0	5	Kyoto	Kinki	West
267	70	Female	-20.1	8.3	0	1	2	5	7	1	3	3	7	0	Hokkaido	Hokkaido	East
268	78	Male	-20.2	9.4	0	1	1	3	4	0	1	3	3	7	Hokkaido	Hokkaido	East
269	64	Female	-20.7	9.4	1	3	3	2	7	0	1	7	1	4	Hokkaido	Hokkaido	East
270	69	Male	-19.3	9.7	0	0	1	1	5	0	1	1	7	2	Hokkaido	Hokkaido	East
271	66	Male	-20.1	9.1	1	1	1	4	3	0	4	3	1	2	Hokkaido	Hokkaido	East
272	69	Male	-19.9	10.1	0	2	1	1	7	1	7	7	7	0	Hokkaido	Hokkaido	East
273	64	Female	-20.4	9.6	0	2	1	1	7	1	7	7	2	0	Hokkaido	Hokkaido	East
274	71	Male	-20.0	10.7	0	0	1	3	7	0	3	3	7	0	Hokkaido	Hokkaido	East
275	63	Male	-21.6	8.9	1	1	1	2	1	1	2	1	2	1	Hokkaido	Hokkaido	East
276	64	Male	-20.2	10.5	0	1	1	2	6	0	0	4	7	0	Hokkaido	Hokkaido	East
277	56	Female	-19.3	9.9	0	2	0	1	5	0	5	1	7	1	Hokkaido	Hokkaido	East
278	34	Male	-19.3	8.4	0	7	3	3	3	0	3	0	3	0	Hokkaido	Hokkaido	East
279	19	Male	-19.7	9.4	0	3	1	5	1	1	3	0	7	0	Hokkaido	Hokkaido	East
280	48	Female	-19.5	9.1	0	3	1	5	1	1	3	1	7	1	Hokkaido	Hokkaido	East
281	67	Male	-18.9	10.8	0	2	1	2	7	0	3	1	7	1	Hokkaido	Hokkaido	East
282	26	Female	-20.0	9.0	3	2	3	3	1	0	0	0	3	0	Kanagawa	Kanto	East
283	24	Female	-19.3	9.2	0	3	0	6	1	0	3	7	7	0	Kanagawa	Kanto	East
284	55	Female	-19.7	9.5	2	2	1	4	4	0	2	2	5	1	Kanagawa	Kanto	East
285	19	Male	-19.6	9.2	2	3	3	3	3	0	5	5	7	0	Kanagawa	Kanto	East
286	20	Male	-19.5	9.7	3	1	0	3	3	0	1	0	2	0	Kanagawa	Kanto	East
287	44	Female	-19.7	9.7	0	4	1	3	3	1	3	2	7	2	Kanagawa	Kanto	East
288	23	Female	-18.7	9.4	3	3	2	7	2	1	3	1	4	2	Kanagawa	Kanto	East
289	29	Male	-18.7	9.7	4	3	2	4	2	0	1	1	3	1	Kanagawa	Kanto	East
290	53	Male	-19.9	9.5	2	2	1	4	5	0	5	5	7	2	Kanagawa	Kanto	East
291	44	Male	-20.8	8.6	1	4	1	7	1	0	5	3	7	0	Saga	Kyushu	West
292	33	Female	-19.4	9.0	1	3	3	5	3	0	6	6	3	3	Saga	Kyushu	West
293	44	Female	-19.6	8.9	2	2	2	2	3	0	0	0	0	0	Saga	Kyushu	West
294	50	Female	-19.6	9.1	1	2	2	7	3	0	4	0	7	0	Saga	Kyushu	West
295	52	Female	-19.9	9.5	1	1	1	1	7	0	7	0	1	2	Saga	Kyushu	West
296	45	Female	-19.4	9.7	2	3	2	7	3	0	2	2	7	1	Saga	Kyushu	West
297	48	Female	-19.1	8.2	0	0	7	0	0	0	0	0	7	0	Saga	Kyushu	West

298	41	Female	-19.9	8.7	1	1	2	2	1	0	0	0	7	1	Saga	Kyushu	West
299	1	Female	-19.3	11.3	0	3	3	6	6	0	6	0	4	0	Saga	Kyushu	West
300	53	Male	-19.6	9.7	0	1	1	2	4	0	5	3	1	0	Saga	Kyushu	West
301	52	Female	-19.6	9.2	0	3	3	2	0	0	4	3	6	0	Saga	Kyushu	West
302	16	Male	-19.6	9.4	0	4	3	4	0	0	1	3	7	0	Saga	Kyushu	West
303	13	Male	-19.1	9.4	0	2	2	2	3	0	2	3	5	0	Saga	Kyushu	West
304	11	Male	-19.4	9.1	0	3	2	0	2	0	2	4	5	1	Saga	Kyushu	West
305	52	Male	-18.6	9.9	0	4	1	3	0	0	5	0	4	0	Saga	Kyushu	West
306	53	Female	-19.8	9.2	1	4	1	3	3	0	4	2	1	0	Saga	Kyushu	West
307	57	Female	-19.2	9.4	2	3	2	5	6	0	2	0	7	1	Saga	Kyushu	West
308	15	Male	-20.4	9.4	1	1	1	1	2	0	1	1	3	0	Saga	Kyushu	West
309	44	Female	-19.8	10.8	6	2	0	7	4	0	3	2	4	0	Saga	Kyushu	West
310	21	Female	-19.6	9.5	1	1	1	6	2	0	1	0	2	1	Saga	Kyushu	West
311	48	Female	-20.0	8.8	1	1	1	7	3	0	2	1	1	1	Saga	Kyushu	West
312	58	Male	-19.4	9.9	1	1	1	7	4	0	3	3	7	0	Saga	Kyushu	West
313	53	Female	-19.3	9.5	1	1	1	7	4	0	3	3	7	0	Saga	Kyushu	West
314	55	Female	-19.5	10.0	1	2	1	4	1	1	4	2	3	1	Saga	Kyushu	West
315	47	Female	-18.9	9.7	1	2	2	7	3	0	3	1	5	1	Saga	Kyushu	West
316	34	Female	-20.0	9.2	1	2	2	6	2	1	3	0	1	2	Aichi	Chubu	East
317	40	Male	-19.2	9.4	1	1	1	3	1	1	3	0	0	0	Aichi	Chubu	East
318	46	Male	-18.8	9.7	1	2	1	6	3	0	1	1	4	0	Aichi	Chubu	East
319	24	Male	-19.9	9.1	4	0	3	3	2	2	0	3	2	0	Aichi	Chubu	East
320	42	Female	-20.4	8.8	0	2	2	5	3	0	6	6	0	3	Aichi	Chubu	East
321	48	Female	-19.0	9.7	0	2	1	5	4	0	7	0	1	0	Aichi	Chubu	East
322	26	Female	-19.9	8.2	2	2	1	7	2	0	2	1	0	1	Aichi	Chubu	East
323	46	Male	-19.9	8.9	1	3	1	7	1	0	4	0	7	1	Saga	Kyushu	West
324	44	Female	-20.4	8.8	1	3	1	7	1	0	4	3	7	1	Saga	Kyushu	West
325	59	Female	-19.7	9.2	1	3	3	2	5	0	5	2	2	1	Saga	Kyushu	West
326	60	Male	-19.7	8.4	3	1	1	7	2	0	3	0	7	1	Saga	Kyushu	West
327	30	Male	-19.3	9.3	1	2	3	1	2	0	2	0	2	0	Saga	Kyushu	West
328	33	Male	-19.1	8.9	2	3	3	3	1	0	3	0	0	1	Saga	Kyushu	West
329	39	Male	-19.8	9.4	2	2	2	4	2	0	2	0	0	0	Saga	Kyushu	West
330	41	Male	-19.0	9.3	1	2	2	2	2	0	2	0	7	1	Saga	Kyushu	West
331	14	Female	-20.3	9.6	1	3	3	6	2	0	2	1	5	1	Saga	Kyushu	West
332	13	Female	-19.5	9.3	0	3	0	7	3	0	0	0	7	3	Saga	Kyushu	West
333	29	Female	-19.0	8.9	1	3	3	4	1	1	2	2	2	2	Saga	Kyushu	West
334	32	Male	-19.2	9.5	1	3	3	3	3	0	3	3	1	1	Saga	Kyushu	West
335	25	Male	-19.0	10.0	0	1	3	4	2	0	1	1	0	1	Kumamoto	Kyushu	West
336	21	Female	-20.0	9.0	0	2	2	2	1	0	0	1	1	2	Kumamoto	Kyushu	West
337	29	Female	-19.3	8.9	2	2	2	4	1	1	5	0	7	2	Saga	Kyushu	West
338	30	Female	-19.4	9.4	0	0	1	4	1	0	0	0	0	1	Saga	Kyushu	West
339	36	Male	-19.1	9.0	0	1	2	5	3	1	2	7	0	0	Aomori	Tohoku	East
340	32	Male	-19.4	9.4	1	2	1	4	4	0	3	3	0	7	Aomori	Tohoku	East
341	38	Female	-19.8	9.0	1	2	1	7	3	2	4	2	7	2	Aomori	Tohoku	East
342	54	Male	-18.5	10.4	0	2	2	3	7	0	2	1	1	1	Aomori	Tohoku	East
343	25	Male	-18.9	9.2	1	1	1	5	3	0	5	1	4	0	Aomori	Tohoku	East
344	25	Female	-18.7	10.9	1	3	2	3	5	0	4	3	0	2	Aomori	Tohoku	East
345	26	Female	-18.6	9.3	1	4	1	5	1	0	2	1	7	0	Aomori	Tohoku	East
346	49	Male	-18.3	9.4	0	3	4	3	7	0	3	0	0	0	Aomori	Tohoku	East
347	47	Female	-19.2	9.7	0	2	3	5	2	0	1	1	2	0	Aomori	Tohoku	East
348	33	Male	-18.7	9.5	1	2	1	4	1	0	2	2	2	1	Aomori	Tohoku	East
349	35	Male	-19.1	9.6	1	4	2	1	3	0	4	1	0	1	Aomori	Tohoku	East
350	20	Female	-19.5	9.4	0	6	1	3	0	2	0	2	4	2	Aomori	Tohoku	East
351	33	Male	-19.1	9.4	2	2	0	2	2	0	2	1	2	2	Aomori	Tohoku	East
352	25	Male	-19.9	8.3	2	3	2	3	0	0	2	2	3	0	Aomori	Tohoku	East
353	35	Female	-19.1	9.4	1	4	2	1	2	0	6	1	4	1	Okinawa	Kyushu	West
354	35	Male	-18.9	9.3	1	3	3	7	1	0	5	0	0	0	Okinawa	Kyushu	West
355	35	Female	-20.2	9.5	0	2	1	7	5	0	0	1	6	0	Okinawa	Kyushu	West
356	37	Male	-19.1	9.5	0	2	2	0	0	0	0	0	0	0	Okinawa	Kyushu	West
357	29	Male	-19.2	9.3	0	3	3	1	1	0	7	7	0	0	Okinawa	Kyushu	West
358	54	Male	-19.1	9.3	1	4	2	1	1	0	2	0	1	0	Okinawa	Kyushu	West
359	60	Male	-20.0	8.9	0	3	3	3	3	0	3	3	3	0	Okinawa	Kyushu	West
360	27	Female	-19.3	9.1	0	1	2	4	1	0	6	7	0	1	Okinawa	Kyushu	West
361	22	Female	-19.6	9.8	3	3	3	3	1	1	1	1	1	1	Okinawa	Kyushu	West
362	23	Male	-19.4	8.6	0	5	2	3	1	0	1	4	0	0	Okinawa	Kyushu	West
363	69	Male	-20.3	9.7	1	1	0	2	4	0	2	2	0	0	Kyoto	Kinki	West
364	36	Female	-20.4	9.7	1	1	1	2	3	0	1	1	0	1	Kyoto	Kinki	West
365	68	Female	-20.0	9.1	1	1	0	3	5	0	3	2	7	3	Kyoto	Kinki	West
366	52	Male	-19.1	9.2	1	3	7	7	7	0	6	1	6	0	Fukuoka	Kyushu	West
367	58	Female	-19.5	9.4	3	3	1	3	3	1	4	3	7	1	Fukuoka	Kyushu	West
368	57	Female	-19.3	10.3	1	3	3	0	7	0	5	5	7	0	Fukuoka	Kyushu	West
369	55	Female	-20.4	9.0	1	0	1	3	7	0	7	1	0	1	Fukuoka	Kyushu	West
370	15	Female	-19.7	8.7	4	4	2	2	3	0	1	0	7	7	Fukuoka	Kyushu	West
371	56	Female	-19.0	10.3	1	4	3	6	5	0	7	5	0	0	Fukuoka	Kyushu	West
372	54	Female	-20.5	9.1	1	1	2	2	2	0	3	1	2	2	Fukuoka	Kyushu	West
373	39	Male	-19.1	9.8	1	2	3	2	3	0	2	1	0	2	Fukuoka	Kyushu	West

374	53	Female	-19.3	10.0	1	2	2	4	3	0	5	1	7	0	Fukuoka	Kyushu	West
375	57	Female	-19.4	9.2	0	0	2	3	3	0	2	0	0	0	Fukuoka	Kyushu	West
376	24	Female	-19.4	9.4	3	3	1	7	3	3	7	0	0	0	Fukuoka	Kyushu	West
377	28	Female	-18.9	9.6	1	1	4	1	5	2	1	1	0	1	Fukuoka	Kyushu	West
378	36	Male	-20.0	8.9	2	3	0	7	3	0	4	2	7	1	Fukuoka	Kyushu	West
379	29	Female	-19.5	9.0	0	2	3	3	3	0	7	1	2	1	Fukuoka	Kyushu	West
380	51	Female	-20.1	9.3	1	3	2	1	1	0	2	0	4	4	Fukuoka	Kyushu	West
381	48	Female	-19.1	9.7	2	3	1	5	3	0	3	0	5	2	Fukuoka	Kyushu	West
382	58	Female	-18.9	9.4	1	4	2	3	5	0	3	3	3	1	Fukuoka	Kyushu	West
383	53	Female	-19.6	9.5	2	3	2	7	1	0	7	1	3	0	Fukuoka	Kyushu	West
384	50	Female	-18.0	9.9	1	1	1	7	2	1	4	4	0	2	Fukuoka	Kyushu	West
385	50	Female	-19.5	9.4	0	3	3	7	3	3	3	3	3	0	Fukuoka	Kyushu	West
386	42	Female	-19.1	9.0	3	3	3	2	2	0	2	6	3	1	Fukuoka	Kyushu	West
387	54	Female	-19.4	9.6	2	3	2	4	3	0	3	1	5	1	Fukuoka	Kyushu	West
388	32	Male	-19.8	8.7	1	3	3	3	2	0	3	2	3	3	Miyagi	Tohoku	East
389	38	Male	-19.3	9.7	2	2	2	3	3	0	3	1	7	3	Miyagi	Tohoku	East
390	38	Female	-19.2	9.8	1	2	1	2	2	0	0	0	7	3	Miyagi	Tohoku	East
391	8	Female	-19.0	9.8	1	2	2	2	2	0	2	0	7	0	Miyagi	Tohoku	East
392	4	Female	-19.3	9.7	1	2	1	2	3	0	2	0	7	3	Miyagi	Tohoku	East
393	37	Male	-20.2	8.4	0	1	2	7	1	0	3	3	7	1	Miyagi	Tohoku	East
394	38	Female	-19.6	8.9	0	1	3	7	1	0	3	3	7	0	Miyagi	Tohoku	East
395	35	Male	-19.9	8.8	2	2	1	2	5	1	2	1	0	1	Miyagi	Tohoku	East
396	38	Male	-19.1	9.3	1	4	6	4	4	0	6	0	7	0	Miyagi	Tohoku	East
397	61	Male	-18.7	10.2	0	1	1	7	7	1	5	2	2	2	Miyagi	Tohoku	East
398	50	Female	-21.1	8.8	0	0	0	1	1	1	1	3	5	1	Miyagi	Tohoku	East
399	19	Female	-19.3	10.0	1	0	0	1	3	0	1	0	0	1	Miyagi	Tohoku	East
400	62	Female	-19.9	9.3	0	5	1	6	2	0	3	1	7	0	Miyagi	Tohoku	East
401	28	Male	-19.4	9.4	1	2	3	4	3	0	2	2	7	3	Miyagi	Tohoku	East
402	26	Male	-19.4	8.9	2	6	4	3	3	0	5	0	4	0	Miyagi	Tohoku	East
403	20	Female	-19.8	8.5	0	1	2	3	3	2	5	6	1	1	Aomori	Tohoku	East
404	21	Female	-19.4	8.4	0	2	2	2	1	0	2	3	0	0	Aomori	Tohoku	East
405	21	Female	-19.3	9.6	2	1	1	2	1	0	0	1	3	0	Aomori	Tohoku	East
406	21	Male	-19.7	8.8	1	3	3	2	3	0	2	1	2	0	Aomori	Tohoku	East
407	19	Female	-20.7	9.1	0	3	1	3	3	0	4	0	0	0	Aomori	Tohoku	East
408	20	Female	-19.9	9.2	0	2	2	3	1	0	1	0	0	0	Aomori	Tohoku	East
409	19	Female	-19.2	9.5	0	0	3	3	0	0	3	3	7	0	Aomori	Tohoku	East
410	19	Female	-19.6	9.5	1	1	1	3	1	1	3	0	0	1	Aomori	Tohoku	East
411	18	Female	-19.0	9.8	0	3	3	7	3	3	3	0	7	0	Aomori	Tohoku	East
412	18	Male	-19.4	9.0	3	3	3	3	0	0	0	3	3	0	Aomori	Tohoku	East
413	19	Female	-19.7	8.9	1	1	3	3	1	1	2	0	1	1	Aomori	Tohoku	East
414	19	Male	-19.5	8.7	1	2	4	4	2	1	1	0	5	1	Miyagi	Tohoku	East
415	19	Female	-19.2	9.9	1	1	1	5	2	2	5	1	6	1	Aomori	Tohoku	East
416	46	Male	-19.3	9.2	1	2	1	2	4	0	2	4	3	0	Yamanashi	Chubu	East
417	37	Male	-19.6	9.4	1	5	0	5	2	0	1	1	2	1	Yamanashi	Chubu	East
418	34	Female	-19.2	9.3	1	2	1	0	1	0	1	1	0	1	Yamanashi	Chubu	East
419	36	Female	-19.1	9.0	2	3	1	5	3	0	1	1	2	2	Yamanashi	Chubu	East
420	44	Female	-19.3	9.0	0	3	2	2	5	0	2	4	0	0	Yamanashi	Chubu	East
421	28	Male	-18.7	9.1	0	3	1	1	2	0	0	0	0	0	Yamanashi	Chubu	East
422	51	Male	-19.0	9.3	0	7	3	3	3	0	3	3	3	0	Yamanashi	Chubu	East
423	30	Male	-19.2	9.5	1	3	0	3	0	0	1	0	0	1	Yamanashi	Chubu	East
424	29	Female	-19.2	8.5	1	1	0	7	1	0	1	0	0	1	Yamanashi	Chubu	East
425	41	Male	-19.0	9.5	2	3	1	4	4	1	4	3	0	0	Yamanashi	Chubu	East
426	41	Male	-19.0	9.4	0	3	1	7	0	0	1	0	3	0	Yamanashi	Chubu	East
427	52	Male	-18.8	9.4	1	1	0	1	1	0	1	2	5	0	Yamanashi	Chubu	East
428	33	Male	-19.3	9.5	0	3	0	3	3	0	0	0	0	3	Yamanashi	Chubu	East
429	29	Male	-19.4	9.1	4	5	3	4	2	0	1	3	1	4	Yamanashi	Chubu	East
430	37	Male	-19.5	8.8	1	1	1	1	1	1	1	5	7	0	Yamanashi	Chubu	East
431	41	Female	-19.1	9.0	0	3	1	1	1	1	2	1	3	0	Yamanashi	Chubu	East
432	31	Female	-19.0	9.4	0	0	0	2	3	1	1	0	0	1	Yamanashi	Chubu	East
433	24	Female	-19.3	9.3	0	0	3	5	1	0	1	0	7	0	Yamanashi	Chubu	East
434	20	Male	-18.7	9.6	7	3	1	3	4	0	1	1	1	0	Yamanashi	Chubu	East
435	26	Female	-18.9	8.9	1	3	2	2	2	2	0	0	3	1	Yamanashi	Chubu	East
436	60	Female	-19.3	10.2	1	2	0	3	5	1	3	0	7	0	Tokushima	Shikoku	West
437	32	Male	-18.6	9.3	1	3	3	5	2	0	5	1	0	2	Tokushima	Shikoku	West
438	64	Male	-19.2	11.2	1	1	0	4	7	1	4	0	0	0	Tokushima	Shikoku	West
439	33	Male	-18.7	9.6	0	0	3	5	5	0	6	0	1	0	Yamaguchi	Chugoku	West
440	38	Male	-18.6	9.7	1	3	2	4	3	0	2	1	3	1	Yamaguchi	Chugoku	West
441	38	Male	-18.7	9.7	0	0	3	5	7	0	5	2	1	0	Yamaguchi	Chugoku	West
442	34	Male	-18.6	9.6	5	4	2	7	0	0	0	0	1	0	Yamaguchi	Chugoku	West
443	39	Male	-18.5	9.5	1	1	2	3	3	0	4	2	1	1	Yamaguchi	Chugoku	West
444	35	Male	-18.6	9.2	1	4	3	7	5	0	6	5	1	0	Yamaguchi	Chugoku	West
445	30	Female	-19.1	10.1	2	3	2	7	3	0	3	3	0	0	Yamaguchi	Chugoku	West
446	52	Female	-19.0	9.2	1	2	3	3	3	3	3	0	7	0	Yamaguchi	Chugoku	West
447	55	Female	-19.4	10.2	0	1	1	0	3	0	1	0	7	0	Yamaguchi	Chugoku	West
448	58	Female	-19.7	9.4	0	3	0	3	3	0	3	3	7	0	Miyagi	Tohoku	East
449	36	Female	-19.5	9.4	1	5	2	0	6	1	7	0	3	0	Miyagi	Tohoku	East

450	38	Male	-19.0	9.8	0	1	1	5	1	1	0	0	4	1	Miyagi	Tohoku	East
451	46	Female	-20.5	8.8	0	2	2	1	4	1	2	1	7	0	Miyagi	Tohoku	East
452	35	Male	-20.0	9.3	0	2	2	3	2	0	3	7	7	1	Miyagi	Tohoku	East
453	58	Female	-20.0	8.5	0	3	1	5	3	0	1	3	2	1	Miyagi	Tohoku	East
454	45	Female	-19.4	9.2	0	2	0	5	2	0	3	5	3	2	Miyagi	Tohoku	East
455	42	Male	-19.1	9.7	2	2	2	3	2	0	1	1	0	1	Nagano	Chubu	East
456	47	Male	-19.0	9.2	0	2	1	3	2	0	1	1	1	1	Nagano	Chubu	East
457	49	Male	-20.2	8.7	0	3	0	3	0	0	3	3	3	0	Nagano	Chubu	East
458	8	Female	-19.1	9.7	2	2	2	2	2	0	1	1	7	7	Nagano	Chubu	East
459	4	Female	-19.4	9.4	2	2	2	2	2	0	1	1	7	7	Nagano	Chubu	East
460	58	Female	-20.2	10.2	0	0	0	0	7	0	7	7	0	0	Yamanashi	Chubu	East
461	34	Female	-19.8	9.6	1	5	2	4	7	0	2	0	2	4	Yamanashi	Chubu	East
462	40	Female	-19.6	8.9	1	2	1	1	1	0	1	2	7	1	Yamanashi	Chubu	East
463	57	Male	-18.8	9.6	0	2	2	1	5	0	2	2	7	7	Yamanashi	Chubu	East
464	28	Female	-19.7	9.1	1	6	3	6	3	1	2	7	0	5	Yamanashi	Chubu	East
465	50	Female	-19.2	9.9	1	3	1	1	3	1	2	1	1	1	Yamanashi	Chubu	East
466	47	Male	-18.9	10.1	0	3	3	4	3	2	3	2	4	3	Yamanashi	Chubu	East
467	49	Male	-19.1	10.0	2	2	2	3	3	0	1	1	0	0	Yamanashi	Chubu	East
468	52	Male	-19.8	9.7	0	4	2	4	3	0	2	1	1	1	Yamanashi	Chubu	East
469	27	Male	-19.3	8.6	1	3	2	1	3	1	1	4	3	0	Aichi	Chubu	East
470	59	Female	-19.4	9.2	1	1	1	1	1	0	1	0	2	0	Fukuoka	Kyushu	West
471	41	Male	-20.3	10.0	2	2	2	2	3	0	2	0	1	1	Fukuoka	Kyushu	West
472	40	Female	-19.4	9.6	0	3	0	0	7	0	7	0	0	0	Fukuoka	Kyushu	West
473	27	Male	-19.5	9.5	3	3	2	2	1	1	1	1	0	0	Fukuoka	Kyushu	West
474	59	Male	-19.3	9.4	1	1	1	1	1	0	1	2	3	0	Fukuoka	Kyushu	West
475	45	Male	-20.1	7.9	1	1	1	0	2	0	0	2	7	3	Fukuoka	Kyushu	West
476	30	Male	-19.4	9.4	2	2	2	4	2	1	3	2	1	1	Fukuoka	Kyushu	West
477	29	Male	-19.0	8.8	0	3	2	7	2	0	7	3	7	3	Fukuoka	Kyushu	West
478	49	Female	-19.4	10.1	0	3	2	1	2	0	5	0	2	1	Fukuoka	Kyushu	West
479	55	Male	-19.6	9.5	1	1	2	4	3	1	4	3	2	1	Fukuoka	Kyushu	West
480	46	Male	-19.8	9.3	1	3	2	4	2	1	1	5	2	3	Fukuoka	Kyushu	West
481	48	Male	-19.9	9.2	2	3	0	3	3	0	1	6	7	1	Tochigi	Kanto	East
482	52	Female	-19.6	9.1	1	1	1	4	2	0	6	1	5	1	Tochigi	Kanto	East
483	27	Male	-19.6	8.9	2	3	2	4	5	1	6	3	2	0	Tochigi	Kanto	East
484	55	Male	-18.6	10.2	1	2	1	6	6	0	7	2	7	0	Tochigi	Kanto	East
485	74	Female	-18.9	9.2	0	2	2	5	5	0	5	0	0	0	Tochigi	Kanto	East
486	61	Female	-20.5	9.7	0	1	1	0	7	1	4	2	0	1	Tochigi	Kanto	East
487	67	Male	-19.5	9.5	1	3	0	4	4	0	0	0	4	1	Tochigi	Kanto	East
488	71	Male	-19.6	9.5	0	1	1	7	3	0	0	2	4	0	Tochigi	Kanto	East
489	30	Male	-19.1	9.3	3	3	3	3	3	3	3	3	0	3	Tochigi	Kanto	East
490	51	Male	-19.0	10.2	0	3	1	1	3	0	1	1	0	0	Tochigi	Kanto	East
491	51	Female	-19.6	9.8	0	3	1	1	3	0	1	1	5	0	Tochigi	Kanto	East
492	15	Female	-18.9	9.8	1	3	2	2	3	0	2	1	0	1	Tochigi	Kanto	East
493	49	Male	-18.9	9.8	0	2	0	2	5	0	4	0	0	1	Tochigi	Kanto	East
494	56	Male	-19.1	10.1	1	2	1	3	3	0	1	2	0	1	Tochigi	Kanto	East
495	54	Female	-18.7	9.6	1	3	1	0	3	0	2	2	7	1	Tochigi	Kanto	East
496	16	Male	-19.7	10.1	1	4	2	5	2	0	4	0	7	0	Tochigi	Kanto	East
497	34	Female	-19.4	9.8	0	3	0	0	3	0	2	3	5	0	Tochigi	Kanto	East
498	33	Female	-19.4	9.3	0	3	1	7	5	1	1	1	4	1	Tochigi	Kanto	East
499	48	Female	-18.6	9.7	0	2	1	5	3	0	4	7	0	1	Tochigi	Kanto	East
500	62	Male	-18.5	9.1	0	3	1	5	5	1	3	6	0	0	Tochigi	Kanto	East
501	47	Female	-20.0	9.4	0	3	2	4	3	0	5	1	2	2	Tochigi	Kanto	East
502	12	Male	-19.8	9.2	1	4	3	4	4	0	4	0	7	0	Tochigi	Kanto	East
503	48	Female	-19.3	8.8	1	4	0	2	3	0	2	5	7	0	Tochigi	Kanto	East
504	36	Female	-19.7	10.0	0	3	0	0	3	0	2	4	0	0	Gunma	Kanto	East
505	47	Male	-19.5	8.6	1	1	1	2	1	0	6	0	0	1	Gunma	Kanto	East
506	48	Male	-19.6	9.8	1	1	0	2	2	0	1	1	7	0	Gunma	Kanto	East
507	6	Male	-19.3	9.9	0	2	1	2	2	0	1	1	1	0	Gunma	Kanto	East
508	7	Male	-19.1	9.4	0	3	1	1	2	0	1	1	5	1	Gunma	Kanto	East
509	38	Female	-19.7	9.2	0	1	0	1	1	0	0	1	0	0	Gunma	Kanto	East
510	36	Male	-20.6	9.5	0	1	0	7	1	0	0	1	0	0	Gunma	Kanto	East
511	80	Male	-19.9	10.5	2	1	0	0	3	1	3	2	3	0	Gunma	Kanto	East
512	64	Male	-20.4	9.9	1	0	0	1	3	0	0	4	0	0	Gunma	Kanto	East
513	62	Female	-20.6	9.0	0	0	0	3	3	0	1	3	0	0	Gunma	Kanto	East
514	84	Female	-19.5	11.5	0	0	0	0	3	0	1	1	0	0	Gunma	Kanto	East
515	59	Male	-18.9	9.9	1	2	1	3	4	0	5	7	2	2	Gunma	Kanto	East
516	57	Female	-19.8	9.4	0	3	1	1	4	0	1	2	7	0	Gunma	Kanto	East
517	91	Female	-20.6	9.6	0	3	1	3	4	0	1	3	0	0	Gunma	Kanto	East
518	7	Male	-18.4	9.8	2	4	1	3	2	0	2	4	7	3	Gunma	Kanto	East
519	10	Male	-19.1	9.6	2	4	1	3	2	0	2	4	5	3	Gunma	Kanto	East
520	37	Female	-18.5	8.7	1	4	0	3	2	0	2	2	3	2	Gunma	Kanto	East
521	48	Male	-18.3	9.8	1	4	0	3	2	0	2	2	1	2	Gunma	Kanto	East
522	60	Female	-19.2	9.3	0	1	1	2	2	0	1	1	0	0	Gunma	Kanto	East
523	59	Male	-19.7	8.7	0	1	0	1	2	0	1	1	4	0	Gunma	Kanto	East
524	15	Female	-19.0	9.5	1	5	1	7	4	2	1	1	2	1	Gunma	Kanto	East
525	79	Female	-18.9	9.9	1	2	1	2	4	3	2	1	7	1	Gunma	Kanto	East

526	40	Female	-20.3	9.6	0	1	1	3	1	0	2	2	1	2	Gunma	Kanto	East
527	44	Female	-19.9	9.4	0	2	1	2	3	0	1	1	2	1	Gunma	Kanto	East
528	8	Female	-19.1	9.3	1	2	2	5	3	0	3	2	7	3	Gunma	Kanto	East
529	65	Female	-20.0	9.3	0	2	1	3	2	0	2	3	4	2	Gunma	Kanto	East
530	60	Female	-19.5	9.5	0	3	3	3	4	1	2	3	7	1	Hiroshima	Chugoku	West
531	24	Female	-19.4	9.0	0	2	1	0	0	0	0	1	7	1	Hiroshima	Chugoku	West
532	Unknown	Male	-19.6	10.2	-	-	-	-	-	-	-	-	-	-	Hiroshima	Chugoku	West
533	66	Female	-18.8	9.3	3	1	1	0	3	0	1	0	0	0	Hiroshima	Chugoku	West
534	66	Male	-18.8	9.8	0	0	0	0	3	0	3	0	7	0	Hiroshima	Chugoku	West
535	23	Male	-18.9	9.7	5	2	3	3	4	0	2	2	7	2	Hiroshima	Chugoku	West
536	14	Female	-19.0	8.9	0	5	1	2	1	0	1	1	6	0	Aomori	Tohoku	East
537	11	Female	-19.6	8.8	0	3	3	3	3	0	2	2	4	3	Aomori	Tohoku	East
538	70	Male	-16.9	12.2	1	0	0	3	7	0	3	1	7	0	Aomori	Tohoku	East
539	45	Female	-19.8	10.2	0	2	3	1	5	0	7	4	0	5	Aomori	Tohoku	East
540	11	Female	-19.3	10.2	0	3	1	4	3	3	4	2	5	1	Aomori	Tohoku	East
541	52	Male	-18.4	9.7	1	1	2	5	5	0	2	1	1	1	Aomori	Tohoku	East
542	18	Male	-20.0	8.5	1	5	1	5	3	0	7	1	5	0	Aomori	Tohoku	East
543	47	Female	-19.6	9.3	0	1	1	4	4	0	7	7	7	0	Aomori	Tohoku	East
544	47	Male	-19.5	8.6	0	4	1	7	1	0	1	5	0	0	Aomori	Tohoku	East
545	94	Female	-20.5	8.9	0	5	0	2	1	0	1	1	0	0	Aomori	Tohoku	East
546	35	Female	-19.6	9.1	0	3	3	3	3	0	7	1	2	0	Aomori	Tohoku	East
547	33	Male	-18.8	9.4	0	3	1	1	3	0	3	5	2	0	Aomori	Tohoku	East
548	40	Male	-19.8	8.6	1	3	3	3	4	0	2	5	2	0	Aomori	Tohoku	East
549	36	Female	-19.4	9.8	1	1	1	3	1	0	1	0	7	0	Kagawa	Shikoku	West
550	41	Female	-19.9	9.5	3	3	3	7	3	0	3	0	7	0	Kagawa	Shikoku	West
551	55	Male	-19.5	9.3	1	3	1	1	2	0	3	0	1	0	Kagawa	Shikoku	West
552	43	Female	-19.1	9.8	1	2	2	3	3	0	1	0	7	5	Kagawa	Shikoku	West
553	73	Male	-19.5	9.1	1	1	1	0	3	0	5	0	7	0	Kagawa	Shikoku	West
554	41	Female	-19.6	9.5	2	2	2	7	3	0	3	0	7	0	Kagawa	Shikoku	West
555	69	Female	-19.0	9.4	2	2	2	2	2	0	7	0	0	0	Kagawa	Shikoku	West
556	64	Female	-19.1	10.8	2	1	3	3	4	0	3	0	4	0	Kagawa	Shikoku	West
557	90	Female	-19.8	10.1	1	0	2	5	2	0	4	0	6	0	Kagawa	Shikoku	West
558	24	Male	-19.4	9.6	1	1	1	0	5	0	2	0	4	0	Chiba	Kanto	East
559	24	Male	-19.4	9.0	1	2	2	2	1	0	0	5	5	0	Chiba	Kanto	East
560	25	Female	-18.8	9.4	3	3	4	5	2	0	2	0	4	2	Kanagawa	Kanto	East
561	25	Female	-18.9	9.2	0	1	3	3	0	0	0	3	0	0	Chiba	Kanto	East
562	24	Female	-19.3	9.4	0	3	3	3	3	0	3	0	7	3	Chiba	Kanto	East
563	22	Male	-19.0	9.3	2	2	2	3	7	0	4	1	1	1	Saitama	Kanto	East
564	23	Male	-19.5	9.6	1	2	2	2	1	1	1	1	6	6	Tokyo	Kanto	East
565	25	Female	-19.7	9.2	1	2	2	3	1	1	0	0	0	0	Chiba	Kanto	East
566	23	Female	-19.9	9.0	1	1	1	6	2	0	1	5	0	0	Chiba	Kanto	East
567	22	Male	-19.2	8.7	0	3	0	1	0	0	3	1	7	0	Chiba	Kanto	East
568	25	Female	-20.0	9.2	1	3	4	4	3	0	2	0	3	3	Chiba	Kanto	East
569	25	Male	-19.5	8.8	1	2	3	6	3	3	1	0	0	1	Tokyo	Kanto	East
570	24	Male	-18.7	9.6	5	5	3	2	3	1	1	1	2	1	Chiba	Kanto	East
571	25	Male	-19.6	9.4	0	0	0	0	1	0	0	1	3	0	Chiba	Kanto	East
572	23	Female	-19.3	8.6	0	3	3	3	0	0	0	0	2	1	Chiba	Kanto	East
573	25	Male	-19.2	9.2	0	2	2	2	1	0	1	0	0	1	Chiba	Kanto	East
574	39	Female	-19.8	9.5	0	3	3	4	2	0	1	3	7	1	Ibaraki	Kanto	East
575	24	Female	-19.6	9.0	0	3	3	0	0	0	2	0	0	0	Chiba	Kanto	East
576	42	Male	-19.2	9.6	2	4	1	1	5	0	7	7	7	0	Chiba	Kanto	East
577	24	Male	-19.2	9.2	3	3	3	5	3	3	3	5	7	0	Saitama	Kanto	East
578	23	Male	-18.6	9.6	2	1	1	3	1	0	1	2	6	0	Chiba	Kanto	East
579	22	Male	-18.8	9.7	0	0	3	0	1	0	0	0	7	0	Chiba	Kanto	East
580	24	Male	-19.4	8.9	1	1	3	2	1	0	2	3	1	0	Tokyo	Kanto	East
581	23	Female	-19.1	9.2	1	2	3	3	1	2	0	0	0	0	Tokyo	Kanto	East
582	23	Female	-19.5	9.2	0	2	2	1	2	0	1	1	1	0	Chiba	Kanto	East
583	23	Male	-19.3	8.8	3	3	1	2	1	0	2	0	3	0	Chiba	Kanto	East
584	22	Female	-19.0	9.1	1	1	1	3	1	1	1	1	1	0	Chiba	Kanto	East
585	24	Female	-19.5	8.4	1	3	3	5	4	1	2	0	7	2	Chiba	Kanto	East
586	24	Male	-19.3	9.1	2	5	3	3	3	3	3	1	0	1	Chiba	Kanto	East
587	26	Female	-19.5	8.6	1	3	3	2	0	0	1	1	1	1	Chiba	Kanto	East
588	24	Male	-19.3	9.8	1	1	1	4	4	0	2	6	3	1	Chiba	Kanto	East
589	25	Female	-18.5	9.5	3	3	1	2	1	0	1	1	4	1	Tokyo	Kanto	East
590	22	Male	-19.6	8.9	3	1	2	2	2	0	0	4	3	0	Chiba	Kanto	East
591	26	Male	-19.2	9.2	2	1	2	3	1	0	1	0	5	0	Chiba	Kanto	East
592	23	Female	-19.8	8.8	0	4	2	7	3	0	2	0	2	1	Saitama	Kanto	East
593	39	Male	-19.5	10.0	1	2	2	3	2	0	2	3	3	2	Ibaraki	Kanto	East
594	22	Female	-20.2	9.7	2	3	3	4	2	0	1	1	3	1	Chiba	Kanto	East
595	23	Female	-20.0	9.2	0	1	1	5	4	1	3	1	7	4	Kanagawa	Kanto	East
596	23	Male	-19.7	9.4	2	2	1	1	0	0	0	0	3	2	Saitama	Kanto	East
597	24	Male	-19.0	9.0	2	3	3	4	3	0	2	0	3	3	Chiba	Kanto	East
598	23	Male	-19.3	9.0	2	3	1	2	3	0	2	3	3	0	Tokyo	Kanto	East
599	24	Female	-19.9	8.9	2	2	1	5	0	0	2	1	3	1	Chiba	Kanto	East
600	24	Male	-19.0	9.3	3	3	3	3	1	0	1	1	3	0	Chiba	Kanto	East
601	23	Male	-19.1	9.8	0	3	1	2	1	0	0	2	3	1	Chiba	Kanto	East

602	24	Male	-19.1	8.5	1	5	3	3	0	0	0	1	3	1	Saitama	Kanto	East
603	26	Male	-18.9	9.1	2	2	3	3	1	0	3	1	3	2	Chiba	Kanto	East
604	33	Male	-19.2	8.8	3	2	2	2	1	0	0	6	7	0	Chiba	Kanto	East
605	24	Female	-19.8	9.0	2	2	2	7	2	0	1	7	7	0	Chiba	Kanto	East
606	30	Male	-19.4	9.3	0	3	3	3	3	3	3	3	3	0	Chiba	Kanto	East
607	22	Female	-19.2	9.4	0	2	2	0	0	2	0	1	5	0	Ibaraki	Kanto	East
608	22	Female	-19.3	8.8	0	0	1	1	2	0	7	0	1	3	Chiba	Kanto	East
609	23	Female	-18.9	9.0	2	1	1	4	1	0	1	1	5	4	Tokyo	Kanto	East
610	23	Female	-19.5	9.3	0	1	0	2	0	0	0	0	7	1	Chiba	Kanto	East
611	23	Female	-20.2	8.9	0	1	2	3	3	0	0	3	7	7	Chiba	Kanto	East
612	83	Female	-19.2	9.1	0	3	1	3	6	0	7	3	7	0	Niigata	Chubu	East
613	54	Female	-18.0	9.6	0	1	0	1	7	0	7	3	7	0	Niigata	Chubu	East
614	57	Female	-19.7	9.0	0	2	1	4	4	0	6	1	3	0	Niigata	Chubu	East
615	28	Male	-18.9	9.1	1	5	3	7	1	0	3	0	0	0	Niigata	Chubu	East
616	58	Male	-19.7	9.2	0	2	2	6	5	0	5	1	0	0	Niigata	Chubu	East
617	33	Female	-19.9	8.3	0	4	2	3	1	0	3	1	4	2	Niigata	Chubu	East
618	38	Female	-19.0	8.5	1	1	1	7	2	2	2	1	3	1	Niigata	Chubu	East
619	40	Female	-18.8	8.6	1	2	1	7	3	0	5	0	0	1	Niigata	Chubu	East
620	83	Male	-19.4	9.9	0	2	0	1	5	0	7	1	7	0	Niigata	Chubu	East
621	26	Female	-18.9	8.8	1	2	2	1	1	0	0	0	7	1	Okinawa	Kyushu	West
622	24	Male	-19.1	9.2	1	5	3	7	1	0	1	1	7	0	Okinawa	Kyushu	West
623	26	Female	-19.6	8.6	1	7	3	2	1	0	2	0	1	1	Okinawa	Kyushu	West
624	25	Female	-19.1	9.5	3	3	2	7	2	0	1	0	3	2	Okinawa	Kyushu	West
625	38	Male	-19.1	10.2	2	2	1	2	3	0	1	2	6	0	Ibaraki	Kanto	East
626	34	Female	-19.4	9.8	1	2	1	1	3	0	1	2	7	3	Ibaraki	Kanto	East
627	30	Female	-19.6	9.2	1	2	2	2	2	1	2	3	0	0	Ibaraki	Kanto	East
628	34	Female	-19.6	8.5	0	1	1	3	4	0	4	4	6	2	Ibaraki	Kanto	East
629	55	Female	-19.7	9.5	0	2	0	5	5	2	1	3	3	4	Ibaraki	Kanto	East
630	36	Female	-19.7	9.0	0	4	1	4	1	0	1	0	6	2	Ibaraki	Kanto	East
631	37	Male	-19.5	9.4	1	2	2	1	3	0	3	2	3	1	Okinawa	Kyushu	West
632	39	Female	-19.5	9.4	1	2	2	3	1	0	2	2	7	6	Okinawa	Kyushu	West
633	35	Female	-18.8	9.1	1	2	2	2	2	0	0	0	7	1	Okinawa	Kyushu	West
634	33	Male	-19.4	9.3	1	2	2	1	2	0	0	0	3	1	Okinawa	Kyushu	West
635	35	Male	-20.4	8.8	1	3	1	7	4	1	2	7	3	1	Niigata	Chubu	East
636	31	Male	-18.9	9.3	0	3	3	2	3	0	7	1	1	0	Niigata	Chubu	East
637	31	Male	-19.0	8.8	1	3	3	3	1	1	1	3	1	1	Niigata	Chubu	East
638	36	Female	-19.4	9.0	0	1	2	2	2	0	2	3	0	1	Niigata	Chubu	East
639	54	Female	-19.9	8.7	0	3	1	7	5	0	2	1	6	1	Niigata	Chubu	East
640	56	Female	-20.1	8.6	0	4	1	2	3	0	2	2	3	0	Niigata	Chubu	East
641	58	Male	-19.3	9.4	0	5	2	3	3	0	3	2	2	0	Niigata	Chubu	East
642	59	Female	-19.6	8.8	0	3	1	5	5	0	2	2	7	7	Niigata	Chubu	East
643	32	Female	-20.2	8.8	0	3	3	7	3	3	3	2	7	1	Niigata	Chubu	East
644	5	Female	-19.0	9.8	0	5	5	3	7	0	2	1	7	0	Niigata	Chubu	East
645	1	Female	-19.3	11.7	0	1	3	1	3	0	4	3	7	2	Niigata	Chubu	East
646	36	Male	-18.5	9.5	1	5	5	5	4	0	2	1	1	1	Niigata	Chubu	East
647	61	Male	-18.5	9.7	0	5	2	7	5	0	5	5	3	3	Niigata	Chubu	East
648	36	Male	-18.8	9.2	1	2	1	2	3	1	3	7	2	2	Niigata	Chubu	East
649	36	Female	-18.8	9.2	0	2	1	1	3	0	3	0	7	1	Niigata	Chubu	East
650	31	Female	-19.6	9.1	1	1	1	3	2	0	5	3	5	0	Niigata	Chubu	East
651	36	Male	-19.6	9.1	1	1	2	0	1	0	2	0	0	0	Niigata	Chubu	East
652	24	Female	-18.3	10.4	1	2	1	1	5	0	1	6	1	0	Okinawa	Kyushu	West
653	23	Female	-19.0	9.9	4	3	2	4	2	0	1	1	1	0	Okinawa	Kyushu	West
654	45	Male	-19.1	9.6	0	4	1	7	1	0	3	1	2	0	Ishikawa	Chubu	East
655	12	Female	-19.4	9.4	2	2	1	6	4	1	4	2	5	2	Ishikawa	Chubu	East
656	51	Female	-18.9	8.5	2	1	1	5	4	2	4	3	1	1	Ishikawa	Chubu	East
657	53	Female	-19.3	9.7	2	2	0	7	4	0	4	2	0	1	Ishikawa	Chubu	East
658	49	Male	-19.8	9.7	0	0	0	1	4	0	5	7	1	0	Ishikawa	Chubu	East
659	38	Male	-19.2	10.1	2	1	1	2	5	0	1	0	1	1	Ishikawa	Chubu	East
660	48	Female	-19.2	10.0	1	5	1	7	6	0	5	1	5	1	Ishikawa	Chubu	East
661	48	Male	-19.4	9.1	3	0	0	3	7	0	7	7	3	0	Ishikawa	Chubu	East
662	47	Female	-19.0	9.6	0	3	2	3	2	2	3	1	4	1	Saitama	Kanto	East
663	48	Female	-19.3	8.9	1	1	1	3	0	0	2	1	2	0	Saitama	Kanto	East
664	28	Female	-19.0	9.3	2	1	1	3	3	0	0	1	2	2	Saitama	Kanto	East
665	30	Female	-20.2	8.7	0	3	0	3	2	0	0	3	3	0	Saitama	Kanto	East
666	42	Female	-20.1	8.9	1	1	1	1	2	2	1	7	0	0	Saitama	Kanto	East
667	48	Female	-20.1	9.1	1	1	0	3	7	0	7	1	1	2	Saitama	Kanto	East
668	47	Female	-20.0	9.6	1	3	1	3	2	1	2	1	1	1	Saitama	Kanto	East
669	56	Female	-19.2	9.0	0	1	1	2	2	0	2	3	1	7	Saitama	Kanto	East
670	51	Male	-19.0	8.7	0	4	3	0	3	0	0	0	0	0	Saitama	Kanto	East
671	52	Female	-20.4	8.8	1	3	3	1	5	2	4	3	2	2	Saitama	Kanto	East
672	50	Female	-19.9	9.3	0	2	2	1	4	0	4	2	3	0	Saitama	Kanto	East
673	54	Female	-20.4	8.9	0	0	1	0	6	0	7	2	1	1	Saitama	Kanto	East
674	46	Female	-19.3	10.1	3	5	3	3	2	0	3	2	2	1	Saitama	Kanto	East
675	46	Female	-19.8	9.0	2	3	2	1	3	0	1	2	3	0	Saitama	Kanto	East
676	53	Female	-19.7	9.2	1	2	1	6	2	0	3	2	0	3	Saitama	Kanto	East
677	44	Female	-19.5	9.2	0	2	3	5	2	0	2	1	2	2	Saitama	Kanto	East

678	56	Female	-20.4	9.3	1	5	1	7	3	0	2	4	0	0	Saitama	Kanto	East
679	52	Female	-20.1	8.1	0	2	2	5	3	0	4	2	2	1	Saitama	Kanto	East
680	39	Female	-19.2	9.2	1	3	1	4	1	1	1	1	1	1	Saitama	Kanto	East
681	53	Female	-19.2	8.5	0	0	3	7	0	0	3	3	7	3	Saitama	Kanto	East
682	30	Female	-19.2	9.6	3	0	3	7	3	0	0	0	0	3	Saitama	Kanto	East
683	35	Female	-20.3	9.5	1	1	1	3	4	0	7	0	7	2	Saitama	Kanto	East
684	26	Male	-19.6	9.8	0	5	1	1	0	0	1	1	7	0	Saitama	Kanto	East
685	55	Female	-20.0	8.6	1	3	1	3	2	1	5	1	3	1	Saitama	Kanto	East
686	48	Female	-18.9	9.5	1	3	2	3	3	3	2	4	7	1	Saitama	Kanto	East
687	42	Female	-19.9	9.5	1	2	1	5	2	0	3	2	2	0	Saitama	Kanto	East
688	25	Male	-19.0	9.2	3	3	3	3	0	0	0	0	0	0	Saitama	Kanto	East
689	38	Male	-19.0	8.6	2	3	1	3	4	0	7	4	7	5	Shimane	Chugoku	West
690	33	Male	-18.5	10.1	3	3	3	3	3	0	3	0	4	0	Shimane	Chugoku	West
691	50	Female	-19.6	9.5	1	2	1	2	2	0	1	0	7	1	Shimane	Chugoku	West
692	49	Female	-18.5	10.2	3	2	2	5	3	0	4	1	5	1	Shimane	Chugoku	West
693	43	Female	-19.4	8.6	1	3	2	3	3	0	5	1	1	1	Shimane	Chugoku	West
694	54	Female	-19.5	9.7	2	4	2	2	5	1	5	0	7	1	Shimane	Chugoku	West
695	59	Female	-19.0	9.4	0	3	0	3	3	0	3	0	3	0	Shimane	Chugoku	West
696	41	Female	-19.3	9.3	1	4	3	5	4	1	2	0	7	1	Shimane	Chugoku	West
697	47	Female	-19.0	9.5	1	3	1	1	7	0	7	0	0	1	Shimane	Chugoku	West
698	44	Female	-19.0	9.8	1	2	4	7	3	2	3	3	0	1	Shimane	Chugoku	West
699	53	Female	-19.6	9.0	3	3	0	7	0	0	3	3	7	0	Shimane	Chugoku	West
700	29	Female	-19.4	9.5	0	1	2	4	0	0	0	0	1	0	Shimane	Chugoku	West
701	27	Male	-18.7	9.4	1	1	2	4	3	0	3	1	3	1	Shimane	Chugoku	West
702	41	Female	-19.5	8.9	0	0	0	7	4	0	2	0	7	4	Shimane	Chugoku	West
703	47	Male	-19.3	9.3	0	3	3	3	3	0	3	3	0	0	Shimane	Chugoku	West
704	46	Female	-18.8	8.7	2	3	4	7	3	2	4	0	7	1	Shimane	Chugoku	West
705	47	Male	-19.3	9.9	1	2	2	2	2	0	4	2	1	1	Shimane	Chugoku	West
706	32	Female	-19.2	9.9	1	3	4	5	3	1	2	1	0	1	Shimane	Chugoku	West
707	5	Female	-19.0	9.7	7	7	7	7	3	0	3	0	7	0	Shimane	Chugoku	West
708	48	Male	-19.2	9.2	1	1	1	3	2	0	2	0	0	0	Shimane	Chugoku	West
709	54	Female	-18.5	9.7	0	4	5	2	4	0	2	0	3	2	Shimane	Chugoku	West
710	50	Female	-18.9	9.9	1	4	3	4	4	0	2	0	7	0	Shimane	Chugoku	West
711	52	Female	-19.0	9.2	0	1	1	4	3	1	5	1	7	0	Shimane	Chugoku	West
712	30	Male	-18.9	9.5	4	4	3	6	3	0	5	1	1	1	Shimane	Chugoku	West
713	44	Male	-18.8	9.6	0	2	2	7	3	0	1	1	0	1	Shimane	Chugoku	West
714	50	Male	-17.6	10.9	1	2	1	4	4	0	2	1	7	0	Shimane	Chugoku	West
715	39	Female	-19.5	8.9	1	2	1	5	3	0	3	3	5	2	Shimane	Chugoku	West
716	25	Female	-20.0	9.3	3	3	3	3	3	0	3	0	7	2	Tottori	Chugoku	West
717	44	Female	-19.2	9.5	2	3	3	6	2	2	5	1	2	1	Tottori	Chugoku	West
718	40	Female	-20.0	9.6	2	1	1	5	2	0	2	3	7	0	Tottori	Chugoku	West
719	45	Female	-19.1	9.0	0	2	2	0	2	0	1	0	7	0	Tottori	Chugoku	West
720	65	Female	-19.8	9.6	0	0	0	4	3	0	1	0	3	0	Tottori	Chugoku	West
721	54	Female	-19.2	10.9	0	1	1	7	6	0	2	0	6	0	Tottori	Chugoku	West
722	67	Male	-18.9	9.5	0	3	2	0	5	0	3	0	7	7	Tottori	Chugoku	West
723	61	Female	-19.5	9.4	1	3	0	6	3	0	0	0	7	0	Tottori	Chugoku	West
724	36	Male	-18.1	9.7	0	4	3	3	1	0	3	3	0	0	Tottori	Chugoku	West
725	32	Female	-19.4	9.1	1	3	3	3	1	0	3	1	6	5	Tottori	Chugoku	West
726	0	Male	-18.4	12.7	0	0	0	0	0	0	0	0	0	0	Tottori	Chugoku	West
727	30	Female	-19.1	9.1	0	3	2	2	3	0	1	0	7	0	Tottori	Chugoku	West
728	54	Female	-19.2	10.0	3	3	2	5	3	3	3	0	1	0	Ehime	Shikoku	West
729	40	Female	-19.1	9.0	1	3	3	3	3	0	1	1	6	3	Ehime	Shikoku	West
730	45	Female	-19.4	8.4	1	1	1	2	2	0	3	0	5	1	Ehime	Shikoku	West
731	49	Female	-19.5	9.1	3	5	3	5	3	0	7	1	7	1	Ehime	Shikoku	West
732	54	Male	-19.4	9.8	3	5	3	5	3	0	7	0	7	1	Ehime	Shikoku	West
733	33	Male	-19.3	8.7	1	4	2	5	1	0	5	0	0	0	Ehime	Shikoku	West
734	42	Female	-19.2	9.1	1	2	2	2	1	0	2	3	7	3	Ehime	Shikoku	West
735	49	Female	-18.2	9.1	1	3	2	7	1	0	5	0	0	1	Ehime	Shikoku	West
736	50	Male	-18.4	10.4	1	3	2	7	1	0	5	0	0	1	Ehime	Shikoku	West
737	37	Female	-19.0	9.7	4	0	1	7	2	0	2	0	1	3	Ehime	Shikoku	West
738	59	Female	-19.3	11.5	0	0	0	7	7	1	6	6	5	2	Ehime	Shikoku	West
739	46	Female	-19.6	8.3	0	2	2	5	2	0	5	3	7	1	Ehime	Shikoku	West
740	35	Female	-21.5	8.4	0	7	1	0	0	0	1	0	0	0	Ehime	Shikoku	West
741	26	Female	-19.2	9.8	4	0	1	6	1	0	4	0	3	0	Ehime	Shikoku	West
742	58	Female	-18.8	9.2	1	1	2	7	3	0	7	7	7	0	Ehime	Shikoku	West
743	50	Male	-20.1	9.8	0	0	0	3	5	0	3	0	0	0	Ehime	Shikoku	West
744	51	Female	-19.3	10.3	0	0	0	7	6	0	3	0	0	0	Ehime	Shikoku	West
745	55	Female	-19.3	9.9	2	2	2	5	3	0	2	2	1	0	Ehime	Shikoku	West
746	31	Female	-18.7	9.1	1	2	3	1	3	0	3	3	7	0	Ehime	Shikoku	West
747	35	Female	-19.3	9.7	1	3	0	2	3	0	4	1	4	0	Ehime	Shikoku	West
748	44	Female	-18.8	8.9	0	0	0	7	3	0	7	7	7	3	Ehime	Shikoku	West
749	50	Female	-19.4	10.0	2	2	2	4	1	1	3	2	4	2	Ehime	Shikoku	West
750	20	Female	-18.8	9.4	3	3	2	3	1	1	2	4	7	2	Ehime	Shikoku	West
751	58	Male	-19.6	8.7	1	1	1	3	2	1	2	3	7	2	Tokyo	Kanto	East
752	35	Female	-19.9	8.6	0	3	2	1	1	0	1	1	1	1	Tokyo	Kanto	East
753	57	Male	-20.5	8.7	1	2	1	1	3	0	3	3	1	2	Tokyo	Kanto	East

754	70	Female	-19.7	9.7	0	3	3	5	5	0	3	0	7	1	Tokyo	Kanto	East
755	69	Female	-20.8	10.6	0	1	2	1	1	1	2	1	0	2	Tokyo	Kanto	East
756	63	Male	-18.8	10.5	2	1	1	2	7	0	7	1	7	1	Tokyo	Kanto	East
757	34	Male	-19.2	9.3	4	4	4	4	1	0	1	1	0	0	Tokyo	Kanto	East
758	39	Male	-18.8	9.7	1	2	1	3	1	0	2	1	3	2	Tokyo	Kanto	East
759	61	Male	-19.0	9.7	0	2	0	1	4	0	2	0	1	1	Shiga	Kinki	West
760	36	Male	-19.3	9.9	1	0	5	5	2	0	2	0	7	0	Kyoto	Kinki	West
761	35	Female	-19.8	9.7	0	2	3	5	3	0	1	2	7	1	Kyoto	Kinki	West
762	34	Female	-21.2	9.1	0	3	4	3	1	0	1	0	0	1	Kyoto	Kinki	West
763	46	Male	-19.5	9.6	0	2	2	5	4	0	1	3	7	1	Kyoto	Kinki	West
764	62	Female	-18.8	9.6	2	2	2	7	7	0	6	6	7	0	Tokyo	Kanto	East
765	50	Male	-19.0	10.6	1	3	3	2	4	0	3	1	5	0	Tokyo	Kanto	East
766	65	Male	-18.6	11.3	1	1	1	2	7	0	3	7	2	1	Tokyo	Kanto	East
767	32	Female	-19.7	9.3	1	2	2	5	1	1	1	3	7	2	Kanagawa	Kanto	East
768	61	Male	-19.6	8.9	0	1	0	3	2	0	1	2	7	1	Tokyo	Kanto	East
769	42	Female	-18.1	9.2	2	2	3	5	2	1	2	2	3	2	Okayama	Chugoku	West
770	18	Female	-18.5	10.0	2	2	2	5	2	1	0	1	2	2	Okayama	Chugoku	West
771	46	Female	-18.6	10.3	2	2	1	1	2	2	0	0	7	0	Okayama	Chugoku	West
772	14	Female	-18.5	10.3	2	2	2	3	2	1	0	1	3	1	Okayama	Chugoku	West
773	16	Female	-18.6	9.9	1	0	1	3	0	6	0	0	6	1	Okayama	Chugoku	West
774	12	Male	-18.5	9.8	2	3	3	4	2	1	3	1	7	0	Okayama	Chugoku	West
775	6	Female	-19.2	10.0	2	2	3	3	3	1	2	2	7	1	Okayama	Chugoku	West
776	56	Male	-19.9	9.6	1	1	1	3	4	0	3	3	0	0	Okayama	Chugoku	West
777	54	Female	-19.2	9.8	1	1	1	4	3	0	3	4	6	0	Okayama	Chugoku	West
778	27	Female	-19.5	8.9	0	5	2	7	0	0	2	5	7	5	Hiroshima	Chugoku	West
779	50	Male	-18.9	10.1	0	2	1	1	1	0	1	0	6	0	Okayama	Chugoku	West
780	48	Female	-19.7	9.4	0	3	1	3	2	0	7	0	7	0	Okayama	Chugoku	West
781	11	Female	-18.9	9.5	0	4	1	3	2	0	3	1	7	1	Okayama	Chugoku	West
782	13	Female	-19.2	9.7	0	6	0	2	4	0	6	3	7	0	Okayama	Chugoku	West
783	54	Female	-18.9	8.9	0	0	2	3	1	0	1	3	7	2	Okayama	Chugoku	West
784	43	Male	-18.9	9.8	1	7	1	2	3	0	1	0	5	0	Okayama	Chugoku	West
785	46	Female	-20.8	8.7	1	2	1	0	3	0	2	7	7	1	Toyama	Chubu	East
786	36	Female	-19.1	8.4	0	0	0	3	1	0	2	3	5	3	Toyama	Chubu	East
787	22	Female	-19.2	8.7	4	4	1	3	0	0	1	0	0	3	Toyama	Chubu	East
788	49	Female	-19.7	8.5	1	5	0	3	0	0	4	2	7	0	Toyama	Chubu	East
789	48	Female	-20.1	8.8	1	2	1	2	2	0	1	0	7	0	Toyama	Chubu	East
790	17	Male	-18.9	9.1	1	5	1	5	1	0	1	0	3	1	Toyama	Chubu	East
791	22	Female	-19.4	8.9	1	2	1	3	1	0	2	1	7	1	Toyama	Chubu	East
792	50	Male	-17.5	10.5	1	2	1	4	3	0	1	3	1	0	Toyama	Chubu	East
793	20	Female	-19.6	9.4	0	0	0	3	2	0	2	0	1	1	Toyama	Chubu	East
794	46	Female	-19.0	9.2	0	0	0	5	5	3	5	0	0	0	Toyama	Chubu	East
795	80	Female	-20.8	8.2	0	0	2	2	4	4	4	0	0	0	Toyama	Chubu	East
796	11	Female	-18.7	9.9	0	3	3	5	4	3	4	0	7	0	Toyama	Chubu	East
797	52	Male	-19.1	9.8	1	2	2	3	3	0	2	2	5	0	Toyama	Chubu	East
798	22	Male	-18.7	9.4	2	2	3	5	1	1	1	1	7	0	Toyama	Chubu	East
799	51	Female	-19.7	9.4	1	1	2	3	2	0	4	2	1	0	Toyama	Chubu	East
800	14	Male	-18.6	9.4	1	2	2	4	4	0	1	2	7	1	Toyama	Chubu	East
801	16	Female	-19.2	9.1	1	0	1	4	4	0	2	4	0	1	Toyama	Chubu	East
802	21	Female	-18.8	9.3	0	3	2	3	2	0	1	1	4	1	Toyama	Chubu	East
803	21	Female	-18.9	9.7	2	3	2	3	3	1	2	0	0	1	Toyama	Chubu	East
804	45	Female	-18.6	10.0	1	2	1	3	3	0	1	2	0	0	Toyama	Chubu	East
805	30	Female	-19.4	9.5	3	3	3	7	2	0	1	0	0	1	Ehime	Shikoku	West
806	73	Female	-19.3	10.4	0	2	2	3	7	0	1	0	0	2	Ehime	Shikoku	West
807	48	Female	-18.1	10.4	1	1	2	3	7	0	5	1	0	1	Ehime	Shikoku	West
808	39	Female	-18.8	9.9	0	3	3	5	3	0	1	0	7	1	Ehime	Shikoku	West
809	34	Female	-19.8	8.5	1	1	1	3	1	0	5	0	0	0	Ehime	Shikoku	West
810	43	Male	-18.9	9.2	1	3	2	4	5	0	3	2	5	0	Tokushima	Shikoku	West
811	42	Male	-19.2	10.5	0	2	2	3	3	0	2	0	7	0	Tokushima	Shikoku	West
812	40	Female	-19.5	9.1	0	5	2	6	3	1	4	0	7	2	Tokushima	Shikoku	West
813	47	Female	-19.7	9.3	1	4	3	4	4	0	5	2	4	6	Tokushima	Shikoku	West
814	43	Female	-19.7	9.1	1	1	1	5	0	0	2	1	1	1	Tokushima	Shikoku	West
815	25	Female	-18.4	10.4	1	3	1	2	3	0	2	0	2	2	Tokushima	Shikoku	West
816	36	Female	-20.2	8.9	0	3	1	3	3	0	7	1	3	1	Tokushima	Shikoku	West
817	33	Male	-19.3	9.9	1	3	2	3	2	1	3	0	2	1	Tokushima	Shikoku	West
818	38	Female	-19.6	9.8	0	0	0	5	0	0	1	0	7	0	Tokushima	Shikoku	West
819	30	Female	-18.4	8.7	1	2	4	6	1	1	4	0	7	1	Tokushima	Shikoku	West
820	38	Male	-19.4	9.3	1	2	1	5	3	0	2	0	0	5	Tokushima	Shikoku	West
821	40	Male	-19.8	9.6	0	3	3	7	2	0	6	2	0	0	Tokushima	Shikoku	West
822	51	Male	-19.3	11.1	2	2	2	3	3	0	0	0	0	0	Tokushima	Shikoku	West
823	41	Female	-19.0	9.9	2	2	1	7	2	0	0	1	3	0	Tokushima	Shikoku	West
824	39	Male	-19.3	9.3	1	2	1	7	0	0	3	0	0	1	Tokushima	Shikoku	West
825	30	Female	-19.5	9.5	1	1	1	4	5	0	0	0	0	0	Tokushima	Shikoku	West
826	36	Male	-18.7	10.3	0	5	5	5	3	0	2	3	1	1	Tokushima	Shikoku	West
827	43	Female	-19.2	9.3	1	3	1	4	2	0	4	0	7	0	Tokushima	Shikoku	West
828	44	Female	-19.3	8.3	0	5	2	3	1	0	2	2	3	1	Tokushima	Shikoku	West
829	49	Female	-19.1	9.1	1	3	3	2	2	0	2	1	7	2	Saitama	Kanto	East

830	37	Male	-19.6	8.5	1	3	3	3	3	0	3	3	3	3	Saitama	Kanto	East
831	36	Female	-20.6	9.4	1	1	1	1	1	0	0	1	4	0	Saitama	Kanto	East
832	26	Female	-19.4	8.9	0	2	2	4	2	0	1	2	3	0	Saitama	Kanto	East
833	37	Female	-19.6	8.5	0	1	1	3	1	0	1	1	5	0	Saitama	Kanto	East
834	29	Male	-19.4	9.0	1	3	1	2	1	0	2	4	0	1	Saitama	Kanto	East
835	27	Female	-19.8	8.7	0	3	3	0	4	0	1	0	0	1	Saitama	Kanto	East
836	27	Male	-19.1	9.7	2	4	1	7	2	0	2	0	0	0	Saitama	Kanto	East
837	57	Female	-20.1	10.0	0	2	0	3	3	0	2	2	5	1	Saitama	Kanto	East
838	51	Female	-19.3	9.8	1	1	1	1	1	0	1	1	0	0	Saitama	Kanto	East
839	47	Female	-20.0	8.7	1	3	2	1	2	0	3	2	7	2	Saitama	Kanto	East
840	48	Female	-20.2	8.4	0	1	1	1	1	0	3	3	7	1	Saitama	Kanto	East
841	47	Female	-18.7	9.8	0	3	3	7	3	0	3	7	0	3	Saitama	Kanto	East
842	41	Female	-20.1	8.3	0	3	3	3	0	0	3	1	7	0	Saitama	Kanto	East
843	50	Female	-20.1	9.4	1	1	1	1	3	0	1	2	0	0	Saitama	Kanto	East
844	51	Female	-19.4	8.9	0	2	1	3	3	0	2	0	5	1	Saitama	Kanto	East
845	51	Female	-19.4	8.7	1	2	1	6	3	0	3	3	5	1	Saitama	Kanto	East
846	55	Female	-19.3	9.0	1	1	0	3	0	1	0	5	1	0	Saitama	Kanto	East
847	53	Female	-19.6	9.4	0	1	1	4	5	0	5	2	1	2	Saitama	Kanto	East
848	46	Female	-18.6	9.6	2	1	0	5	2	0	1	0	0	0	Saitama	Kanto	East
849	58	Female	-19.6	10.3	0	1	1	2	6	0	2	5	7	1	Saitama	Kanto	East
850	47	Female	-19.8	9.4	1	5	3	3	3	0	4	1	5	2	Saitama	Kanto	East
851	58	Female	-19.5	9.0	0	1	3	0	2	0	2	3	4	1	Saitama	Kanto	East
852	28	Female	-19.5	9.5	0	3	2	3	2	0	1	2	0	2	Saitama	Kanto	East
853	49	Female	-21.0	9.0	0	2	2	1	2	0	0	0	6	0	Shimane	Chugoku	West
854	17	Female	-20.5	8.7	0	2	2	2	0	0	0	0	6	0	Shimane	Chugoku	West
855	60	Female	-19.5	9.7	0	3	1	5	1	0	4	0.5	1	0	Okayama	Chugoku	West
856	33	Female	-19.5	9.4	0	3	1	5	2	0	2	2	7	1	Okayama	Chugoku	West
857	37	Female	-19.5	11.0	0	3	3	7	7	0	3	0	3	3	Okayama	Chugoku	West
858	35	Female	-20.1	9.3	1	3	1	7	3	0	3	0	7	0	Okayama	Chugoku	West
859	44	Female	-19.1	8.8	1	3	1	4	4	0	1	1	6	1	Okayama	Chugoku	West
860	58	Female	-18.8	10.2	0	1	2	2	7	0	0	1	7	0	Okayama	Chugoku	West
861	36	Female	-20.3	9.1	4	4	6	3	7	0	3	0	0	0	Okayama	Chugoku	West
862	40	Female	-19.2	9.4	2	0	0	3	3	0	1	0	7	0	Okayama	Chugoku	West
863	12	Female	-19.1	9.5	0	2	2	7	3	0	2	2	5	2	Tottori	Chugoku	West
864	32	Male	-19.2	9.2	0	3	1	4	2	0	5	4	7	0	Tottori	Chugoku	West
865	44	Male	-19.1	10.2	0	2	2	7	3	0	2	0	0	1	Tottori	Chugoku	West
866	29	Female	-19.3	9.4	0	4	1	4	1	0	4	2	7	0	Tottori	Chugoku	West
867	Unknown	Female	-19.3	9.9	0	3	1	1	2	0	1	2	7	0	Tottori	Chugoku	West
868	18	Female	-19.4	9.4	0	2	2	7	3	0	2	1	1	2	Tottori	Chugoku	West
869	16	Male	-19.0	9.3	0	2	2	7	3	0	2	3	3	3	Tottori	Chugoku	West
870	39	Female	-18.9	9.4	0	2	2	7	3	0	2	1	1	2	Tottori	Chugoku	West
871	68	Male	-20.2	9.4	0	0	1	2	3	0	2	1	1	1	Hokkaido	Hokkaido	East
872	42	Female	-19.9	8.9	1	2	1	2	2	0	2	2	7	2	Hokkaido	Hokkaido	East
873	68	Female	-19.9	10.0	0	0	1	2	5	0	4	2	7	3	Hokkaido	Hokkaido	East
874	53	Female	-19.5	9.9	1	3	1	2	3	1	2	1	0	1	Hokkaido	Hokkaido	East
875	53	Male	-18.0	10.0	1	1	1	5	5	0	3	2	0	3	Hokkaido	Hokkaido	East
876	65	Female	-19.7	9.9	0	3	1	3	4	0	3	4	7	7	Hokkaido	Hokkaido	East
877	56	Male	-19.3	9.6	0	1	1	3	3	0	2	1	5	1	Hokkaido	Hokkaido	East
878	43	Male	-19.1	9.8	1	1	1	3	1	0	3	3	1	1	Hokkaido	Hokkaido	East
879	33	Male	-19.1	9.1	1	2	2	3	1	0	3	3	0	0	Iwate	Tohoku	East
880	36	Female	-20.0	8.8	0	0	0	3	0	0	2	3	0	0	Iwate	Tohoku	East
881	66	Female	-20.1	8.9	0	2	1	1	3	0	3	5	0	0	Iwate	Tohoku	East
882	37	Female	-20.2	9.3	0	3	2	1	3	0	4	0	0	0	Iwate	Tohoku	East
883	54	Female	-19.7	9.3	0	3	1	2	3	0	1	0	0	2	Iwate	Tohoku	East
884	26	Female	-19.1	8.7	1	2	2	4	3	0	2	4	0	3	Iwate	Tohoku	East
885	27	Female	-18.9	9.5	1	2	2	1	1	0	1	2	0	0	Iwate	Tohoku	East
886	26	Female	-18.6	9.6	1	4	2	3	2	0	5	5	0	3	Iwate	Tohoku	East
887	30	Female	-19.1	9.0	0	4	3	3	0	0	0	4	0	0	Iwate	Tohoku	East
888	30	Male	-19.3	10.2	1	1	4	1	1	0	3	6	0	0	Iwate	Tohoku	East
889	40	Male	-19.2	9.9	1	1	1	6	2	0	6	1	0	1	Iwate	Tohoku	East
890	34	Male	-19.1	9.3	0	3	1	5	1	0	1	0	7	3	Iwate	Tohoku	East
891	33	Male	-19.4	9.2	1	3	3	3	2	2	4	2	3	1	Iwate	Tohoku	East
892	35	Male	-19.5	8.6	1	1	2	1	2	0	3	5	1	1	Iwate	Tohoku	East
893	38	Male	-19.4	9.4	0	2	1	6	3	0	5	6	7	2	Iwate	Tohoku	East
894	33	Male	-19.3	9.2	0	2	2	3	2	0	2	5	7	2	Iwate	Tohoku	East
895	28	Female	-19.9	9.4	0	2	1	7	2	0	3	7	7	0	Iwate	Tohoku	East
896	34	Male	-18.9	8.7	1	5	0	5	2	0	0	3	4	0	Iwate	Tohoku	East
897	24	Female	-19.3	9.5	1	2	1	5	4	0	2	3	2	2	Iwate	Tohoku	East
898	55	Female	-18.7	9.4	3	3	3	7	7	0	7	3	3	3	Iwate	Tohoku	East
899	54	Female	-19.7	9.4	1	2	1	4	7	0	7	5	2	1	Iwate	Tohoku	East
900	53	Female	-20.2	9.3	1	3	3	4	3	1	5	3	2	1	Iwate	Tohoku	East
901	54	Female	-19.2	9.6	1	3	1	5	4	1	6	3	7	0	Iwate	Tohoku	East
902	53	Female	-19.1	9.6	0	1	2	4	6	0	4	3	7	1	Iwate	Tohoku	East
903	69	Female	-20.7	8.9	3	4	1	2	3	0	5	2	5	0	Iwate	Tohoku	East
904	4	Female	-19.0	9.4	0	4	3	6	3	0	5	2	7	0	Iwate	Tohoku	East
905	80	Female	-19.5	8.2	0	3	1	7	2	0	5	0	7	0	Iwate	Tohoku	East

906	38	Female	-19.8	9.4	0	5	1	3	1	0	2	1	7	0	Iwate	Tohoku	East
907	54	Female	-20.5	10.0	0	1	1	2	3	3	6	1	7	7	Iwate	Tohoku	East
908	44	Male	-19.4	9.3	0	1	0	1	2	0	3	1	4	1	Iwate	Tohoku	East
909	40	Female	-19.1	9.4	0	3	2	1	1	0	3	1	3	1	Iwate	Tohoku	East
910	34	Female	-19.2	8.5	0	1	1	4	0	0	2	1	7	2	Iwate	Tohoku	East
911	38	Male	-19.1	9.4	2	2	0	2	0	0	1	3	2	0	Iwate	Tohoku	East
912	67	Male	-18.5	9.2	1	2	1	3	2	0	3	2	2	0	Shizuoka	Chubu	East
913	10	Female	-18.5	9.7	2	5	1	0	0	0	1	2	5	2	Shizuoka	Chubu	East
914	38	Male	-18.5	9.8	3	3	1	7	6	0	1	1	0	0	Shizuoka	Chubu	East
915	69	Female	-19.3	9.3	1	2	1	2	2	0	2	0	7	2	Shizuoka	Chubu	East
916	47	Male	-20.1	9.2	1	3	4	7	4	0	2	2	7	0	Shizuoka	Chubu	East
917	77	Female	-20.9	8.3	0	0	2	2	2	0	2	0	2	0	Shizuoka	Chubu	East
918	35	Male	-18.9	9.3	2	2	1	2	1	0	0	0	0	0	Shizuoka	Chubu	East
919	63	Male	-19.0	10.4	1	3	2	5	4	0	4	2	7	0	Shizuoka	Chubu	East
920	29	Male	-19.2	10.2	2	4	2	4	3	0	3	2	1	0	Shizuoka	Chubu	East
921	58	Female	-19.2	9.0	1	3	1	3	4	0	3	1	7	0	Shizuoka	Chubu	East
922	24	Male	-19.0	9.5	2	3	3	3	3	1	5	1	2	1	Ibaraki	Kanto	East
923	52	Female	-18.9	9.4	1	1	2	3	2	0	2	1	1	1	Shizuoka	Chubu	East
924	10	Female	-19.1	9.4	1	3	3	5	3	0	2	2	7	3	Shizuoka	Chubu	East
925	31	Female	-19.1	9.9	0	3	0	0	7	3	3	3	0	0	Shizuoka	Chubu	East
926	23	Female	-19.1	9.1	3	3	2	7	2	2	0	1	0	0	Shizuoka	Chubu	East
927	6	Female	-18.7	10.0	2	5	1	1	3	0	1	2	3	2	Shizuoka	Chubu	East
928	63	Female	-18.9	9.9	2	4	2	1	4	0	3	1	0	0	Shizuoka	Chubu	East
929	36	Female	-18.9	9.8	2	5	1	5	4	0	2	1	3	1	Shizuoka	Chubu	East
930	29	Male	-19.0	9.9	0	1	3	6	3	0	7	2	1	0	Oita	Kyushu	West
931	65	Female	-19.5	9.2	1	1	2	2	7	1	7	2	7	0	Kochi	Shikoku	West
932	42	Male	-19.6	9.0	2	1	1	5	2	0	1	1	0	0	Kochi	Shikoku	West
933	42	Male	-19.1	8.7	1	0	3	2	2	0	3	0	0	0	Kochi	Shikoku	West
934	22	Female	-18.9	8.9	0	3	3	5	0	0	0	0	0	0	Kochi	Shikoku	West
935	23	Female	-18.4	9.7	2	3	3	6	3	0	5	0	7	2	Kochi	Shikoku	West
936	43	Female	-19.1	9.0	1	2	2	1	1	0	1	0	2	2	Nara	Kinki	West
937	6	Female	-18.6	9.9	4	3	2	5	3	2	1	2	6	0	Nara	Kinki	West
938	40	Female	-19.7	9.5	0	3	3	3	3	0	0	0	7	3	Tokushima	Shikoku	West
939	34	Female	-20.1	10.1	1	3	1	2	1	0	3	0	1	1	Tokushima	Shikoku	West
940	38	Female	-19.6	10.1	1	1	1	7	1	1	1	0	0	0	Tokushima	Shikoku	West
941	36	Female	-19.0	9.6	1	2	2	2	2	0	2	1	2	1	Tokushima	Shikoku	West
942	59	Female	-19.5	8.9	2	2	2	7	0	0	3	3	2	2	Tokushima	Shikoku	West
943	34	Female	-19.9	9.2	1	3	0	0	3	0	0	0	7	2	Tokushima	Shikoku	West
944	1	Male	-18.5	12.2	0	0	0	0	0	0	0	0	1	0	Tokushima	Shikoku	West
945	25	Female	-18.7	9.6	3	3	3	7	7	0	3	0	0	3	Tokushima	Shikoku	West
946	36	Female	-19.9	9.2	0	5	1	4	1	1	4	1	2	1	Tokushima	Shikoku	West
947	44	Male	-18.9	9.2	1	2	2	3	2	0	3	0	7	1	Gifu	Chubu	East
948	36	Male	-18.9	9.7	2	2	1	5	3	0	2	0	0	2	Wakayama	Kinki	West
949	97	Male	-16.3	6.7	0	0	0	0	0	0	0	0	0	0	Kochi	Shikoku	West
950	47	Female	-20.0	9.3	0	1	0	2	0	0	1	1	1	2	Nara	Kinki	West
951	54	Female	-19.6	9.6	2	5	4	7	4	1	2	5	7	0	Nara	Kinki	West
952	29	Female	-19.3	9.5	1	1	1	3	5	1	0	2	6	1	Nara	Kinki	West
953	36	Male	-19.4	9.5	1	1	1	3	2	1	1	1	3	2	Nara	Kinki	West
954	36	Female	-19.8	10.0	1	1	1	3	1	0	2	1	7	2	Nara	Kinki	West
955	6	Male	-19.0	9.8	1	1	1	3	1	0	2	5	7	2	Nara	Kinki	West
956	6	Male	-18.9	9.7	1	1	1	3	1	0	2	5	7	2	Nara	Kinki	West
957	36	Male	-19.8	9.4	0	1	2	2	3	0	3	1	0	0	Nara	Kinki	West
958	34	Male	-19.8	9.7	1	3	1	1	7	0	5	5	0	3	Nara	Kinki	West
959	13	Male	-19.0	8.7	2	2	1	3	2	0	2	1	6	1	Nara	Kinki	West
960	42	Female	-19.5	9.0	2	2	1	3	2	1	1	1	6	3	Nara	Kinki	West
961	41	Male	-18.7	10.4	3	2	2	3	5	1	1	1	0	0	Nara	Kinki	West
962	49	Female	-19.2	10.0	0	0	0	7	2	0	0	0	0	0	Nara	Kinki	West
963	32	Male	-19.1	8.7	0	2	2	3	2	0	3	5	1	1	Nara	Kinki	West
964	25	Female	-19.5	9.1	1	1	1	6	4	0	6	3	1	1	Nara	Kinki	West
965	32	Male	-18.4	7.8	0	0	3	7	1	0	7	5	0	1	Nara	Kinki	West
966	32	Male	-18.9	9.9	1	3	1	4	4	0	7	0	3	1	Mie	Kinki	West
967	25	Female	-18.8	10.0	1	1	2	6	2	1	2	1	2	2	Nara	Kinki	West
968	11	Male	-19.2	10.0	3	3	2	0	3	0	1	1	7	1	Nara	Kinki	West
969	47	Female	-19.3	9.1	1	1	1	5	7	0	3	3	7	1	Nara	Kinki	West
970	37	Male	-19.1	9.7	3	3	2	1	2	0	1	1	0	0	Nara	Kinki	West
971	38	Female	-19.8	9.7	2	3	2	1	3	0	1	1	0	0	Nara	Kinki	West
972	25	Female	-19.5	10.3	2	2	1	6	2	2	3	0	7	0	Nara	Kinki	West
973	53	Female	-19.8	9.5	1	2	2	3	1	1	3	1	1	0	Nara	Kinki	West
974	43	Male	-18.4	9.8	0	2	4	4	4	0	4	2	3	2	Tokushima	Shikoku	West
975	32	Male	-19.1	9.5	1	4	2	5	2	0	3	0	0	2	Tokushima	Shikoku	West
976	36	Female	-19.0	9.6	0	2	4	3	4	0	1	0	4	2	Tokushima	Shikoku	West
977	28	Female	-18.7	9.4	1	6	3	3	3	0	2	1	4	4	Gifu	Chubu	East
978	31	Female	-19.0	9.6	1	4	2	5	2	0	3	0	5	2	Tokushima	Shikoku	West
979	37	Male	-19.3	10.1	3	3	3	5	7	1	5	0	0	0	Tokushima	Shikoku	West
980	45	Male	-18.6	10.2	1	1	1	3	3	0	2	1	7	1	Tokushima	Shikoku	West
981	37	Female	-20.3	8.4	0	0	0	3	1	0	2	1	0	3	Okinawa	Kyushu	West

982	49	Female	-19.4	9.3	0	3	3	3	3	0	3	3	7	7	Hokkaido	Hokkaido	East
983	62	Female	-19.6	10.2	1	1	1	7	2	0	2	3	2	1	Hokkaido	Hokkaido	East
984	42	Male	-20.4	8.4	1	2	2	3	3	0	2	1	1	1	Hokkaido	Hokkaido	East
985	74	Female	-20.1	10.0	0	3	0	2	3	0	5	1	7	0	Hokkaido	Hokkaido	East
986	27	Female	-19.7	9.5	0	3	1	7	3	0	1	2	0	0	Hokkaido	Hokkaido	East
987	56	Male	-18.7	10.0	1	3	0	7	3	0	1	2	7	1	Hokkaido	Hokkaido	East
988	60	Male	-19.2	10.5	5	5	5	0	7	0	2	0	0	0	Hokkaido	Hokkaido	East
989	35	Male	-19.3	9.0	0	1	3	7	2	0	7	7	1	3	Fukui	Chubu	East
990	36	Female	-18.8	8.8	3	4	6	2	0	0	1	1	0	1	Fukui	Chubu	East
991	35	Female	-19.1	9.7	3	3	3	3	3	0	3	0	0	1	Fukui	Chubu	East
992	32	Male	-18.7	10.3	1	2	2	2	1	1	1	0	1	1	Fukui	Chubu	East
993	29	Male	-18.5	9.5	2	2	1	0	2	0	2	3	0	0	Fukui	Chubu	East
994	25	Male	-18.9	9.1	1	1	2	2	2	1	1	1	1	1	Fukui	Chubu	East
995	37	Male	-19.4	9.1	0	2	3	7	3	0	2	3	2	1	Fukui	Chubu	East
996	7	Male	-18.8	9.1	3	2	3	7	3	0	6	5	6	2	Fukui	Chubu	East
997	9	Female	-19.6	8.4	3	2	3	7	3	0	2	4	5	1	Fukui	Chubu	East
998	35	Female	-20.8	7.9	0	2	3	7	3	0	2	3	2	1	Fukui	Chubu	East
999	33	Female	-18.3	8.6	0	0	1	7	0	0	1	0	7	2	Fukui	Chubu	East
1000	34	Male	-19.1	9.2	7	2	2	2	0	0	0	0	0	0	Fukui	Chubu	East
1001	36	Male	-19.2	9.2	2	1	1	7	0	0	1	0	1	1	Fukui	Chubu	East
1002	36	Male	-19.1	8.6	1	1	2	6	1	0	1	0	7	5	Fukui	Chubu	East
1003	72	Male	-19.4	9.3	1	1	0	2	2	0	2	0	1	0	Gifu	Chubu	East
1004	69	Female	-20.0	7.9	0	2	1	4	2	1	6	1	7	2	Gifu	Chubu	East
1005	41	Female	-19.8	9.3	1	4	4	2	1	0	2	0	0	0	Gifu	Chubu	East
1006	40	Male	-19.7	9.4	1	4	4	2	1	0	2	0	0	0	Gifu	Chubu	East
1007	6	Female	-20.2	9.5	1	5	3	0	1	2	3	1	0	1	Gifu	Chubu	East
1008	44	Female	-18.4	9.3	0	3	3	3	3	3	3	0	7	0	Gifu	Chubu	East
1009	46	Male	-19.6	9.5	1	5	3	3	1	1	1	1	1	2	Gifu	Chubu	East
1010	15	Male	-19.3	9.0	1	5	3	3	1	1	2	1	7	2	Gifu	Chubu	East
1011	12	Male	-18.9	9.2	1	5	3	4	5	1	3	2	7	2	Gifu	Chubu	East
1012	44	Female	-19.2	9.0	1	2	2	3	1	0	2	1	0	0	Gifu	Chubu	East
1013	44	Male	-18.8	9.7	1	2	4	7	1	0	1	1	0	0	Gifu	Chubu	East
1014	39	Male	-18.7	8.9	2	2	3	2	3	1	4	0	2	0	Gifu	Chubu	East
1015	9	Male	-18.9	9.6	1	3	5	2	2	0	3	1	5	1	Gifu	Chubu	East
1016	37	Female	-18.8	9.5	1	3	6	6	1	0	0	0	7	3	Kochi	Shikoku	West
1017	35	Female	-19.1	10.3	1	2	1	7	3	0	1	0	1	0	Kochi	Shikoku	West
1018	39	Male	-18.8	9.5	0	7	3	3	3	0	0	0	0	0	Kochi	Shikoku	West
1019	38	Female	-19.0	9.5	0	0	0	3	7	0	3	0	0	0	Kochi	Shikoku	West
1020	3	Female	-18.6	9.8	3	7	3	3	7	0	3	3	7	0	Kochi	Shikoku	West
1021	15	Female	-18.8	10.0	3	7	0	3	7	0	0	0	7	0	Kochi	Shikoku	West
1022	10	Female	-18.8	10.1	3	7	3	3	7	0	3	0	7	0	Kochi	Shikoku	West
1023	31	Male	-19.0	9.4	0	4	3	5	1	0	3	1	4	0	Ehime	Shikoku	West
1024	38	Male	-18.9	8.6	1	3	3	7	1	0	2	0	0	0	Ehime	Shikoku	West
1025	34	Male	-18.3	10.4	1	1	2	6	6	0	7	0	0	1	Ehime	Shikoku	West
1026	59	Female	-18.7	9.3	0	1	1	3	3	0	2	0	1	0	Okinawa	Kyushu	West
1027	8	Male	-19.2	9.4	2	3	1	0	1	0	3	0	5	1	Okinawa	Kyushu	West
1028	12	Female	-19.6	8.8	2	4	1	7	1	0	4	0	5	1	Okinawa	Kyushu	West
1029	40	Male	-18.5	9.5	3	4	1	2	1	0	3	0	0	1	Okinawa	Kyushu	West
1030	1.9	Female	-20.8	9.8	0	2	0	3	1	0	5	1	7	1	Okinawa	Kyushu	West
1031	8	Female	-19.2	8.8	2	4	1	4	1	0	3	0	5	1	Okinawa	Kyushu	West
1032	31	Female	-19.9	8.9	2	3	1	3	4	0	5	5	0	0	Okinawa	Kyushu	West
1033	39	Female	-20.1	8.5	1	5	1	2	1	0	4	0	0	0	Okinawa	Kyushu	West
1034	19	Male	-19.3	9.3	2	2	6	3	1	0	0	0	4	0	Oita	Kyushu	West
1035	19	Female	-19.3	10.0	0	2	2	3	0	0	0	7	7	0	Oita	Kyushu	West
1036	21	Female	-19.8	9.1	0	0	7	0	0	0	0	3	0	0	Oita	Kyushu	West
1037	18	Female	-19.7	9.9	0	1	3	3	2	0	3	3	0	0	Oita	Kyushu	West
1038	18	Female	-18.6	9.2	3	3	3	3	3	0	0	0	0	0	Oita	Kyushu	West
1039	23	Female	-20.4	8.5	0	0	3	4	0	0	1	2	3	0	Oita	Kyushu	West
1040	20	Female	-20.1	9.9	0	2	7	7	0	0	1	0	2	3	Oita	Kyushu	West
1041	40	Male	-19.1	9.7	1	1	1	3	0	0	1	0	7	3	Nagasaki	Kyushu	West
1042	33	Male	-19.3	10.1	0	3	3	0	3	0	1	0	7	0	Nagasaki	Kyushu	West
1043	33	Female	-19.7	9.5	2	2	1	4	3	0	1	3	7	7	Nagasaki	Kyushu	West
1044	1	Female	-18.6	12.6	2	2	1	0	3	0	1	3	7	7	Nagasaki	Kyushu	West
1045	43	Male	-18.7	9.8	1	2	2	3	2	0	2	1	2	2	Nagasaki	Kyushu	West
1046	44	Female	-19.9	9.6	1	2	2	3	2	0	2	1	2	2	Nagasaki	Kyushu	West
1047	47	Male	-19.3	9.0	1	1	1	0	1	0	2	1	0	0	Nagasaki	Kyushu	West
1048	36	Male	-18.7	9.8	1	1	0	1	6	0	0	0	1	1	Nagasaki	Kyushu	West
1049	28	Female	-18.7	9.2	1	2	2	5	2	0	1	3	7	3	Nagasaki	Kyushu	West
1050	35	Male	-19.5	9.4	2	2	1	4	4	0	3	2	2	2	Nagasaki	Kyushu	West
1051	34	Male	-19.0	9.4	2	4	3	3	3	0	2	1	7	2	Miyazaki	Kyushu	West
1052	17	Female	-19.0	10.4	1	1	1	7	3	0	0	1	7	0	Miyazaki	Kyushu	West
1053	53	Female	-18.7	9.2	3	3	0	3	0	0	3	0	7	3	Miyazaki	Kyushu	West
1054	57	Male	-19.3	10.3	4	4	3	2	2	0	2	2	0	0	Miyazaki	Kyushu	West
1055	53	Female	-19.8	10.3	3	3	3	1	3	0	2	1	0	1	Miyazaki	Kyushu	West
1056	34	Female	-19.1	9.7	1	4	2	2	2	0	4	5	4	4	Miyazaki	Kyushu	West
1057	13	Male	-18.8	9.9	0	4	3	3	2	0	4	7	7	6	Miyazaki	Kyushu	West

1058	50	Female	-20.3	9.6	0	7	3	7	3	0	7	0	0	0	Miyazaki	Kyushu	West
1059	62	Female	-20.3	10.2	1	3	3	7	3	1	4	1	3	1	Miyazaki	Kyushu	West
1060	36	Female	-19.7	9.9	1	3	2	4	4	0	1	1	7	0	Miyazaki	Kyushu	West
1061	49	Female	-18.9	9.4	1	2	2	7	1	0	2	1	5	0	Miyazaki	Kyushu	West
1062	35	Female	-18.6	10.0	1	5	1	2	1	0	1	0	1	1	Miyazaki	Kyushu	West
1063	48	Female	-20.0	10.1	1	1	1	3	1	0	1	1	0	0	Miyazaki	Kyushu	West
1064	35	Female	-19.5	9.4	0	5	5	7	3	3	7	2	7	0	Miyazaki	Kyushu	West
1065	19	Male	-18.6	10.2	0	7	3	7	3	0	7	0	3	0	Miyazaki	Kyushu	West
1066	51	Female	-19.4	9.8	0	0	3	3	3	0	3	0	0	0	Miyazaki	Kyushu	West
1067	61	Female	-20.1	9.8	0	4	3	3	2	0	4	7	4	3	Miyazaki	Kyushu	West
1068	40	Female	-19.4	9.3	1	3	3	7	3	0	3	0	3	3	Miyazaki	Kyushu	West
1069	36	Female	-19.2	10.1	5	1	0	5	1	0	2	1	5	0	Kumamoto	Kyushu	West
1070	39	Female	-19.6	9.0	0	5	2	7	2	0	3	2	7	0	Kumamoto	Kyushu	West
1071	48	Female	-19.7	9.3	0	3	0	5	1	0	7	0	6	0	Kumamoto	Kyushu	West
1072	39	Female	-18.7	10.5	1	7	4	4	3	0	4	0	0	0	Kumamoto	Kyushu	West
1073	41	Female	-17.9	9.6	1	4	3	5	1	0	2	0	0	0	Kumamoto	Kyushu	West
1074	48	Female	-20.4	9.3	0	3	0	7	3	0	3	3	0	0	Kumamoto	Kyushu	West
1075	51	Female	-19.3	9.5	1	2	2	5	3	0	2	1	6	1	Kumamoto	Kyushu	West
1076	39	Female	-19.3	10.1	1	0	3	3	3	0	2	0	1	5	Kumamoto	Kyushu	West
1077	40	Female	-20.4	9.3	1	5	1	3	3	0	3	0	1	1	Kumamoto	Kyushu	West
1078	30	Female	-19.1	9.5	2	3	3	4	3	0	3	1	0	0	Kumamoto	Kyushu	West
1079	55	Male	-18.3	9.8	2	2	1	2	2	0	2	1	5	7	Hyogo	Kinki	West
1080	41	Male	-19.4	9.5	2	3	3	1	0	0	1	0	1	0	Hyogo	Kinki	West
1081	43	Male	-18.9	10.0	2	2	2	0	3	1	2	0	1	1	Hyogo	Kinki	West
1082	48	Male	-19.0	10.4	2	2	1	2	1	0	1	1	0	1	Hyogo	Kinki	West
1083	41	Female	-19.2	9.7	1	4	2	3	3	0	3	1	2	0	Hyogo	Kinki	West
1084	38	Male	-20.0	10.4	1	2	2	5	2	0	5	0	2	2	Hyogo	Kinki	West
1085	51	Male	-19.2	9.5	0	4	3	3	1	0	2	0	7	0	Hyogo	Kinki	West
1086	41	Male	-17.4	9.9	0	2	5	6	2	0	3	2	1	1	Hyogo	Kinki	West
1087	47	Male	-19.6	9.0	0	2	2	7	2	0	3	1	0	3	Hyogo	Kinki	West
1088	58	Male	-19.4	9.6	1	5	1	5	6	0	3	4	7	0	Hyogo	Kinki	West
1089	54	Male	-17.9	11.1	2	3	4	4	3	0	4	0	0	1	Hyogo	Kinki	West
1090	41	Male	-19.4	9.0	2	5	3	5	2	0	1	0	0	1	Hyogo	Kinki	West
1091	46	Male	-19.4	9.5	1	1	2	2	3	0	4	1	2	4	Hyogo	Kinki	West
1092	62	Male	-19.3	10.6	1	2	2	1	4	0	2	1	3	2	Yamagata	Tohoku	East
1093	52	Female	-18.8	11.7	0	1	2	0	6	0	0	2	2	1	Yamagata	Tohoku	East
1094	58	Female	-19.6	9.4	0	3	0	3	3	0	4	5	0	5	Akita	Tohoku	East
1095	54	Female	-20.1	9.7	0	2	1	2	5	0	4	7	5	2	Akita	Tohoku	East
1096	39	Female	-20.5	9.6	0	3	0	3	3	0	3	3	3	3	Akita	Tohoku	East
1097	40	Female	-19.2	9.2	0	2	2	2	3	0	3	7	3	2	Akita	Tohoku	East
1098	52	Female	-19.2	9.3	1	4	1	6	1	0	3	1	6	1	Akita	Tohoku	East
1099	50	Female	-19.2	9.3	0	3	2	5	2	0	7	0	5	0	Akita	Tohoku	East
1100	75	Female	-19.5	8.9	0	1	1	7	6	0	7	5	7	2	Akita	Tohoku	East
1101	54	Female	-19.4	9.1	0	1	1	2	3	0	1	4	1	0	Akita	Tohoku	East
1102	50	Female	-18.6	9.7	1	2	2	5	3	0	4	6	7	5	Akita	Tohoku	East
1103	18	Male	-19.8	9.0	0	3	1	0	1	1	0	7	7	1	Fukushima	Tohoku	East
1104	18	Female	-20.2	8.8	1	2	2	6	2	2	1	6	5	2	Fukushima	Tohoku	East
1105	51	Female	-20.7	9.4	0	1	1	2	6	0	7	6	1	2	Fukushima	Tohoku	East
1106	18	Female	-20.3	8.9	2	3	4	3	3	3	6	1	0	0	Fukushima	Tohoku	East
1107	18	Female	-19.2	9.0	0	2	2	0	0	0	0	3	0	0	Fukushima	Tohoku	East
1108	18	Female	-19.2	9.1	0	1	2	6	0	0	1	0	5	0	Fukushima	Tohoku	East
1109	27	Male	-18.6	10.0	6	3	2	6	3	2	0	0	1	3	Gifu	Chubu	East
1110	32	Male	-18.7	9.1	0	3	3	6	3	0	3	0	0	1	Gifu	Chubu	East
1111	34	Female	-18.5	10.6	5	5	6	2	4	2	4	5	7	0	Gifu	Chubu	East
1112	53	Female	-19.3	10.0	0	3	3	3	3	3	7	0	0	0	Gifu	Chubu	East
1113	41	Female	-19.0	9.3	1	2	3	6	4	1	4	3	3	2	Gifu	Chubu	East
1114	30	Female	-18.9	9.6	2	2	2	1	2	0	2	0	3	1	Gifu	Chubu	East
1115	41	Male	-19.7	9.1	0	1	1	1	2	2	4	1	0	0	Gifu	Chubu	East
1116	27	Female	-19.6	10.1	1	1	1	3	2	0	5	1	0	1	Gifu	Chubu	East
1117	30	Female	-19.1	9.7	0	1	1	3	3	1	3	0	3	0	Gifu	Chubu	East
1118	26	Female	-19.3	9.4	1	2	2	3	3	0	3	0	7	0	Gifu	Chubu	East
1119	38	Female	-19.4	9.3	3	3	1	4	3	0	2	2	4	1	Yamagata	Tohoku	East
1120	43	Female	-19.8	9.1	1	2	2	5	2	0	1	1	1	7	Yamagata	Tohoku	East
1121	36	Female	-19.8	9.2	0	7	1	1	0	0	1	1	0	1	Yamagata	Tohoku	East
1122	35	Male	-19.1	9.3	0	1	1	0	3	0	1	7	0	1	Yamagata	Tohoku	East
1123	24	Male	-19.5	9.1	1	1	1	3	1	1	1	0	5	6	Yamagata	Tohoku	East
1124	23	Female	-19.6	8.9	0	2	3	1	1	0	1	2	0	1	Yamagata	Tohoku	East
1125	35	Male	-19.2	9.8	0	2	2	3	2	0	2	0	1	1	Kagawa	Shikoku	West
1126	25	Female	-20.1	10.4	2	2	2	7	1	1	2	0	0	1	Kagawa	Shikoku	West
1127	44	Female	-20.1	9.1	2	2	3	6	2	1	3	1	0	1	Kagawa	Shikoku	West
1128	55	Female	-19.0	10.2	1	2	1	3	5	0	5	1	7	3	Kagawa	Shikoku	West
1129	38	Female	-20.1	8.6	0	2	2	2	2	1	3	1	7	2	Yamagata	Tohoku	East
1130	39	Male	-20.4	8.5	2	2	2	3	4	0	3	5	5	1	Yamagata	Tohoku	East
1131	46	Female	-19.2	8.3	2	3	1	1	0	1	2	1	2	2	Yamagata	Tohoku	East
1132	53	Female	-19.7	8.9	3	2	1	2	2	1	7	2	1	1	Yamagata	Tohoku	East
1133	31	Female	-19.4	9.3	1	1	1	3	1	0	2	2	0	0	Wakayama	Kinki	West

1134	22	Female	-19.3	9.4	0	3	3	7	2	0	0	0	7	0	Wakayama	Kinki	West
1135	3	Female	-19.8	9.2	1	2	0	1	2	1	6	0	7	3	Wakayama	Kinki	West
1136	37	Female	-19.2	9.2	1	2	0	2	2	1	5	0	7	2	Wakayama	Kinki	West
1137	45	Female	-19.5	9.8	2	3	1	7	2	0	1	0	0	0	Wakayama	Kinki	West
1138	19	Male	-18.8	9.8	3	3	0	7	0	0	0	0	0	0	Wakayama	Kinki	West
1139	16	Male	-18.7	9.4	7	2	1	7	1	2	1	3	3	1	Wakayama	Kinki	West
1140	36	Female	-19.6	9.0	1	1	2	3	2	0	3	0	7	1	Mie	Kinki	West
1141	56	Male	-19.5	9.5	0	1	0	1	2	0	2	1	7	2	Oita	Kyushu	West
1142	23	Male	-18.6	9.7	1	2	4	2	5	0	1	0	0	0	Oita	Kyushu	West
1143	27	Male	-19.4	9.4	0	3	0	3	3	0	3	3	3	0	Oita	Kyushu	West
1144	27	Female	-18.9	9.9	0	1	1	5	3	0	3	3	7	0	Ishikawa	Chubu	East
1145	6	Male	-19.1	8.4	1	4	4	4	3	0	6	2	7	1	Ishikawa	Chubu	East
1146	1	Female	-19.2	11.4	1	4	3	4	4	0	6	1	7	1	Ishikawa	Chubu	East
1147	17	Male	-20.5	8.9	0	4	3	4	1	0	3	1	4	0	Ishikawa	Chubu	East
1148	16	Male	-18.8	8.8	0	5	3	4	1	0	4	0	7	0	Ishikawa	Chubu	East
1149	12	Male	-19.2	9.1	1	6	2	3	2	0	2	1	7	0	Ishikawa	Chubu	East
1150	42	Female	-19.6	8.7	0	4	2	2	1	0	4	1	6	6	Ishikawa	Chubu	East
1151	14	Male	-19.5	8.9	1	1	2	4	3	0	1	4	0	1	Ishikawa	Chubu	East
1152	60	Male	-19.0	9.9	1	2	0	4	3	1	2	0	4	0	Ishikawa	Chubu	East
1153	44	Female	-20.3	8.5	0	1	1	3	3	1	3	3	7	2	Ishikawa	Chubu	East
1154	62	Male	-18.7	10.8	1	1	0	4	5	0	1	1	0	0	Ishikawa	Chubu	East
1155	55	Female	-19.1	9.3	1	5	2	7	6	5	5	1	7	3	Ishikawa	Chubu	East
1156	65	Female	-20.2	8.4	1	2	0	3	4	0	0	3	0	1	Ishikawa	Chubu	East
1157	67	Female	-20.7	9.2	3	3	1	4	5	0	3	5	6	1	Ishikawa	Chubu	East
1158	52	Male	-19.5	9.1	2	1	0	1	2	0	1	1	1	0	Ishikawa	Chubu	East
1159	63	Male	-19.8	9.4	6	3	0	5	6	0	3	1	0	0	Ishikawa	Chubu	East
1160	62	Male	-17.7	11.0	0	3	0	0	7	0	0	0	0	0	Ishikawa	Chubu	East
1161	73	Male	-19.5	8.6	3	1	1	7	3	0	2	2	0	6	Ishikawa	Chubu	East
1162	75	Male	-19.5	8.9	1	4	3	3	4	0	1	3	1	1	Ishikawa	Chubu	East
1163	18	Female	-19.0	9.4	1	1	1	2	2	0	3	2	7	1	Ishikawa	Chubu	East
1164	26	Female	-18.5	9.8	0	3	2	3	2	0	2	0	7	0	Mie	Kinki	West
1165	39	Female	-19.4	8.7	0	2	1	2	2	0	2	3	6	0	Mie	Kinki	West
1166	48	Male	-18.3	8.9	1	2	1	3	2	0	1	3	1	1	Mie	Kinki	West
1167	71	Female	-19.2	10.6	1	3	6	7	5	5	1	1	0	0	Mie	Kinki	West
1168	61	Female	-19.5	9.5	1	1	1	2	5	3	1	1	0	0	Mie	Kinki	West
1169	42	Male	-17.7	9.9	1	1	0	2	2	0	2	0	7	0	Mie	Kinki	West
1170	44	Male	-19.2	9.7	1	2	1	2	2	0	1	0	3	1	Mie	Kinki	West
1171	37	Female	-19.5	9.1	1	3	3	3	3	0	6	5	2	3	Mie	Kinki	West
1172	16	Male	-19.3	10.2	2	1	1	4	2	0	0	0	7	2	Mie	Kinki	West
1173	45	Female	-19.7	10.3	2	1	1	3	2	0	1	0	3	1	Mie	Kinki	West
1174	20	Male	-18.6	9.5	3	0	0	7	3	0	0	3	7	0	Mie	Kinki	West
1175	19	Male	-18.6	10.0	2	2	1	3	2	1	0	0	6	6	Mie	Kinki	West
1176	5	Female	-18.7	9.5	1	2	1	1	3	0	0	1	5	2	Mie	Kinki	West
1177	39	Female	-19.5	9.3	1	2	1	7	3	0	2	1	3	1	Mie	Kinki	West
1178	39	Male	-18.9	9.3	1	2	1	7	3	0	1	0	1	1	Mie	Kinki	West
1179	9	Female	-19.2	9.9	1	3	1	3	3	0	0	0	5	1	Mie	Kinki	West
1180	40	Male	-19.1	9.4	1	4	2	2	1	1	4	2	2	1	Mie	Kinki	West
1181	37	Female	-18.8	9.4	0	1	1	1	4	0	2	1	2	1	Mie	Kinki	West
1182	58	Male	-19.3	9.8	1	2	2	1	1	0	5	2	0	6	Kagawa	Shikoku	West
1183	5	Male	-19.1	9.8	3	3	3	6	4	0	3	0	7	2	Yamaguchi	Chugoku	West
1184	32	Female	-19.3	8.2	0	3	1	2	0	0	2	1	7	1	Yamaguchi	Chugoku	West
1185	33	Male	-19.0	9.3	3	2	2	3	3	0	1	1	5	1	Yamaguchi	Chugoku	West
1186	51	Female	-19.3	9.3	1	2	1	7	3	0	2	0	7	1	Yamaguchi	Chugoku	West
1187	43	Male	-20.1	10.1	0	0	0	3	1	0	7	0	0	0	Yamaguchi	Chugoku	West
1188	34	Male	-19.3	10.3	1	1	1	3	3	0	3	0	6	3	Yamaguchi	Chugoku	West
1189	31	Female	-19.1	9.2	2	5	2	7	2	0	3	0	0	0	Yamaguchi	Chugoku	West
1190	3	Female	-20.2	10.3	0	2	2	0	3	0	5	7	2	5	Yamaguchi	Chugoku	West
1191	33	Female	-19.0	9.3	0	3	3	6	1	0	1	1	7	0	Yamaguchi	Chugoku	West
1192	50	Male	-18.0	11.0	2	2	1	5	3	0	1	0	1	0	Yamaguchi	Chugoku	West
1193	36	Male	-18.4	9.8	2	2	2	7	3	1	3	1	7	7	Yamaguchi	Chugoku	West
1194	44	Male	-19.0	9.6	3	3	3	7	3	0	3	3	0	2	Yamaguchi	Chugoku	West
1195	44	Female	-19.6	9.3	1	2	1	5	3	0	4	1	7	1	Fukui	Chubu	East
1196	45	Male	-18.4	9.3	2	1	2	7	3	0	5	0	0	4	Fukui	Chubu	East
1197	39	Female	-18.7	9.2	3	3	3	0	3	3	0	0	3	3	Fukui	Chubu	East
1198	26	Female	-19.7	9.0	1	2	3	7	3	2	3	1	4	1	Kochi	Shikoku	West
1199	26	Male	-19.2	9.1	0	2	3	7	3	1	2	1	0	0	Kochi	Shikoku	West
1200	55	Male	-19.2	8.5	2	4	5	4	3	0	3	3	3	3	Kochi	Shikoku	West
1201	29	Male	-19.3	8.7	0	3	3	2	1	0	0	0	0	0	Fukushima	Tohoku	East
1202	34	Male	-19.1	8.8	3	2	2	1	0	0	0	0	1	0	Fukushima	Tohoku	East
1203	23	Female	-19.4	8.7	1	2	2	6	4	1	4	2	3	2	Fukushima	Tohoku	East
1204	32	Male	-19.6	10.1	1	3	2	0	4	0	2	2	0	0	Fukushima	Tohoku	East
1205	60	Female	-19.0	10.0	0	3	2	2	4	2	1	7	3	1	Fukushima	Tohoku	East
1206	35	Female	-19.7	8.9	1	2	1	7	3	0	1	2	7	1	Fukushima	Tohoku	East
1207	26	Female	-19.3	8.3	1	2	2	3	1	0	0	1	2	0	Fukushima	Tohoku	East
1208	31	Female	-19.6	9.1	0	3	0	3	3	0	0	3	0	0	Fukushima	Tohoku	East
1209	51	Male	-19.5	9.3	0	2	2	7	3	0	7	7	0	1	Kagoshima	Kyushu	West

1210	44	Female	-19.9	8.8	0	2	2	7	3	0	5	1	7	0	Kagoshima	Kyushu	West
1211	16	Female	-20.1	8.8	0	3	2	7	2	0	5	2	2	1	Kagoshima	Kyushu	West
1212	14	Male	-19.4	8.8	0	3	2	7	2	0	5	3	7	1	Kagoshima	Kyushu	West
1213	13	Female	-19.2	9.2	0	3	2	6	2	0	5	0	6	1	Kagoshima	Kyushu	West
1214	83	Female	-19.2	10.4	0	3	4	1	4	0	1	0	7	0	Kagoshima	Kyushu	West
1215	17	Female	-19.4	9.2	1	5	5	7	3	0	2	1	3	1	Kagoshima	Kyushu	West
1216	50	Female	-19.6	9.2	0	0	0	3	3	0	2	1	0	2	Kagoshima	Kyushu	West
1217	56	Male	-19.0	9.9	3	3	3	5	5	0	5	0	0	1	Kagoshima	Kyushu	West
1218	24	Female	-19.6	9.6	1	3	6	7	4	0	1	1	1	1	Kagoshima	Kyushu	West
1219	74	Male	-18.1	11.1	1	3	1	2	4	0	6	0	2	0	Kagoshima	Kyushu	West
1220	5	Female	-19.4	9.5	1	1	1	2	1	0	3	0	6	2	Kagoshima	Kyushu	West
1221	3	Female	-19.4	9.2	1	1	1	2	3	0	4	1	6	2	Kagoshima	Kyushu	West
1222	33	Female	-19.9	9.4	1	1	1	4	1	0	5	1	2	0	Kagoshima	Kyushu	West
1223	44	Male	-18.8	10.9	0	4	3	1	4	0	5	2	0	0	Kagoshima	Kyushu	West
1224	8	Male	-18.5	10.2	2	4	3	2	3	0	6	1	5	0	Kagoshima	Kyushu	West
1225	6	Female	-18.7	10.0	1	4	3	1	3	0	6	0	2	0	Kagoshima	Kyushu	West
1226	39	Female	-19.9	9.3	1	4	3	1	2	0	6	2	3	0	Kagoshima	Kyushu	West
1227	42	Female	-18.9	9.6	0	4	1	1	1	0	5	1	7	0	Kagoshima	Kyushu	West
1228	75	Female	-19.3	10.4	1	3	3	2	4	0	6	1	1	0	Kagoshima	Kyushu	West
1229	10	Female	-18.9	9.6	1	4	1	1	1	1	5	1	7	0	Kagoshima	Kyushu	West
1230	34	Female	-19.8	9.3	1	2	2	2	1	0	1	0	2	0	Kyoto	Kinki	West
1231	33	Female	-19.2	9.5	0	3	3	2	1	0	3	0	7	0	Kyoto	Kinki	West
1232	35	Female	-20.2	8.5	2	1	0	7	1	0	3	0	7	0	Kyoto	Kinki	West
1233	32	Female	-19.4	9.5	0	3	3	7	3	0	3	2	6	0	Kyoto	Kinki	West
1234	35	Female	-19.3	9.5	0	0	1	3	1	0	2	1	0	1	Kyoto	Kinki	West
1235	33	Female	-19.1	10.1	0	1	2	5	2	0	3	0	0	0	Kyoto	Kinki	West
1236	34	Female	-19.2	9.7	1	3	3	7	3	0	3	0	3	3	Kyoto	Kinki	West
1237	53	Female	-19.4	9.4	1	2	2	1	3	1	3	1	7	1	Kyoto	Kinki	West
1238	34	Female	-19.7	9.0	1	2	1	4	3	0	4	2	5	0	Kyoto	Kinki	West
1239	44	Male	-18.6	10.0	2	2	1	4	3	0	2	1	0	1	Kyoto	Kinki	West
1240	11	Female	-19.1	9.4	1	3	2	5	2	0	4	2	2	1	Kyoto	Kinki	West
1241	40	Female	-19.9	10.0	1	5	2	3	3	0	2	1	3	3	Kyoto	Kinki	West
1242	41	Female	-18.8	9.4	1	1	2	2	2	0	1	1	0	0	Kyoto	Kinki	West
1243	37	Female	-22.0	9.0	1	1	2	4	2	0	1	1	2	1	Kyoto	Kinki	West
1244	7	Female	-18.8	9.7	1	2	2	4	3	0	3	0	7	2	Kyoto	Kinki	West
1245	44	Female	-18.5	9.2	0	2	2	4	3	0	3	4	1	1	Kyoto	Kinki	West
1246	74	Male	-17.9	10.6	1	1	1	2	3	0	5	1	7	1	Kyoto	Kinki	West
1247	73	Female	-19.6	9.8	1	1	2	2	3	0	5	1	7	0	Kyoto	Kinki	West
1248	43	Female	-19.7	9.2	1	2	2	6	1	0	0	3	4	1	Hiroshima	Chugoku	West
1249	42	Male	-19.4	9.2	0	2	2	7	2	0	0	1	7	1	Hiroshima	Chugoku	West
1250	10	Male	-18.6	9.4	1	2	2	5	1	0	0	3	7	2	Hiroshima	Chugoku	West
1251	9	Male	-19.3	8.8	1	2	1	2	1	0	0	7	6	1	Hiroshima	Chugoku	West
1252	76	Male	-19.7	11.1	1	3	2	3	2	0	5	2	4	0	Hiroshima	Chugoku	West
1253	70	Female	-19.7	10.0	1	3	3	7	3	0	7	3	3	0	Hiroshima	Chugoku	West
1254	56	Male	-19.6	9.9	0	1	1	7	5	0	1	1	1	1	Nagano	Chubu	East
1255	58	Male	-19.7	9.6	0	3	1	4	2	0	2	2	7	1	Nagano	Chubu	East
1256	51	Male	-19.8	9.2	1	3	2	1	6	0	2	5	2	3	Nagano	Chubu	East
1257	42	Male	-19.5	9.3	1	3	1	7	1	0	1	4	1	0	Nagano	Chubu	East
1258	42	Male	-19.5	9.5	1	4	2	3	2	0	2	7	2	0	Nagano	Chubu	East
1259	42	Male	-19.8	9.3	0	3	1	4	2	0	2	3	1	1	Nagano	Chubu	East
1260	57	Female	-20.0	9.1	0	2	1	2	4	0	3	7	7	0	Nagano	Chubu	East
1261	40	Female	-19.4	8.7	0	4	1	5	5	1	3	1	6	7	Nagano	Chubu	East
1262	11	Female	-19.1	9.3	1	3	1	4	4	1	5	1	7	6	Nagano	Chubu	East
1263	14	Female	-19.5	9.0	1	2	1	3	2	2	3	1	7	2	Nagano	Chubu	East
1264	44	Male	-19.4	9.2	1	1	0	5	3	0	3	0	7	0	Nagano	Chubu	East
1265	59	Male	-18.7	10.2	0	1	0	2	7	0	0	1	0	1	Nagano	Chubu	East
1266	39	Female	-19.5	9.4	1	3	2	6	3	0	4	2	5	2	Nagano	Chubu	East
1267	12	Female	-18.9	9.8	3	3	2	7	3	0	5	3	7	0	Nagano	Chubu	East
1268	43	Male	-19.7	9.9	1	2	1	2	2	0	2	1	2	1	Nagano	Chubu	East
1269	41	Male	-18.4	10.1	1	5	2	6	3	0	1	2	0	0	Mie	Kinki	West
1270	48	Female	-19.9	9.3	2	2	1	7	3	1	3	2	7	2	Mie	Kinki	West
1271	19	Female	-19.7	9.4	0	4	0	5	2	0	1	0	1	0	Kyoto	Kinki	West
1272	18	Female	-19.3	9.1	1	4	1	7	3	0	3	0	0	1	Mie	Kinki	West
1273	50	Female	-19.5	9.0	2	3	2	7	4	0	2	3	7	1	Mie	Kinki	West
1274	49	Male	-19.9	9.9	0	0	0	3	4	1	7	4	0	1	Oita	Kyushu	West
1275	52	Male	-19.0	9.9	1	1	1	5	3	0	2	2	7	1	Oita	Kyushu	West
1276	48	Female	-19.8	8.3	0	2	2	2	3	0	7	1	1	2	Oita	Kyushu	West
1277	49	Male	-17.6	10.5	3	3	3	2	3	0	1	1	3	1	Oita	Kyushu	West
1278	49	Male	-20.4	9.4	1	2	3	2	3	0	3	1	3	1	Oita	Kyushu	West
1279	57	Male	-19.1	9.4	2	2	2	6	4	0	7	6	7	6	Oita	Kyushu	West
1280	33	Female	-19.4	9.2	1	3	3	5	3	0	3	2	5	3	Oita	Kyushu	West
1281	32	Female	-19.3	9.2	1	2	2	7	2	0	3	2	7	0	Oita	Kyushu	West
1282	49	Male	-19.6	9.4	1	1	3	7	7	0	3	7	3	1	Oita	Kyushu	West
1283	29	Female	-19.0	9.3	0	1	3	7	3	0	7	1	4	0	Oita	Kyushu	West
1284	38	Male	-19.1	9.9	1	2	2	7	2	0	7	0	0	0	Kagoshima	Kyushu	West
1285	30	Female	-19.0	9.1	1	7	3	2	3	1	7	1	7	1	Kagoshima	Kyushu	West

1286	3	Male	-19.0	9.8	1	2	2	3	2	0	7	1	7	2	Kagoshima	Kyushu	West
1287	0	Female	-18.3	12.3	0	0	2	1	2	0	2	0	0	0	Kagoshima	Kyushu	West
1288	45	Female	-20.1	8.7	0	2	2	0	1	0	1	1	0	0	Kagoshima	Kyushu	West
1289	70	Female	-20.6	9.5	1	4	2	5	3	0	7	4	2	1	Kagoshima	Kyushu	West
1290	42	Female	-19.8	9.2	2	3	3	7	5	0	7	3	7	1	Kagoshima	Kyushu	West
1291	76	Male	-20.6	9.9	1	3	2	7	5	0	7	4	2	1	Kagoshima	Kyushu	West
1292	25	Female	-19.5	9.2	0	3	2	3	3	1	3	0	3	0	Kagoshima	Kyushu	West
1293	25	Male	-19.0	9.2	0	3	3	2	2	2	3	0	2	0	Kagoshima	Kyushu	West
1294	55	Female	-19.6	9.9	1	5	1	5	5	0	4	2	5	1	Miyazaki	Kyushu	West
1295	37	Male	-19.5	9.9	3	2	2	5	4	0	6	6	2	1	Miyazaki	Kyushu	West
1296	25	Female	-19.0	9.3	2	2	1	4	4	0	2	5	0	1	Miyazaki	Kyushu	West
1297	26	Male	-19.3	9.6	0	3	3	4	3	0	2	2	7	1	Miyazaki	Kyushu	West
1298	26	Female	-19.7	9.6	0	7	5	5	5	0	4	1	4	3	Miyazaki	Kyushu	West
1299	23	Female	-19.6	8.9	0	3	3	7	3	0	7	3	7	3	Miyazaki	Kyushu	West
1300	0	Female	-19.1	11.5	0	1	1	2	3	1	2	0	3	1	Kagoshima	Kyushu	West
1301	21	Female	-19.0	10.5	0	2	2	0	0	0	0	0	2	1	Yamagata	Tohoku	East
1302	22	Female	-19.7	8.7	2	3	5	3	1	0	3	0	3	3	Yamagata	Tohoku	East
1303	26	Female	-19.9	9.0	0	2	4	3	0	0	2	0	7	1	Yamagata	Tohoku	East
1304	23	Female	-19.6	8.8	0	3	3	5	1	0	5	0	1	2	Yamagata	Tohoku	East
1305	33	Male	-19.0	9.7	3	3	3	3	3	1	3	2	7	1	Nara	Kinki	West

Title: Homogeneous diet of contemporary Japanese inferred from stable isotope ratios of hair
Authors: Soichiro Kusaka, Eriko Ishimaru, Fujio Hyodo, Gakuhari Takashi, Minoru Yoneda, Takakazu Yumoto, Ichiro Tayasu

Supplementary Table S5. Isotopic measurement results for contemporary hair from Korea, India, Mongolia

No.	Country	Age	Sex	$\delta^{13}C$	$\delta^{15}N$	Beef	Pork	Chicken	Egg	Marine fish	Fresh water fish	Tofu	Natto	Milk	Cheese
1	Korea	27	Female	-19.6	8.9	0	3	1	2	0	2	2	0	5	0
2	Korea	28	Female	-19.3	9.5	0	0	0	3	3	0	3	0	3	0
3	Korea	7	Male	-18.9	10.8	2	2	2	3	4	0	3	1	7	2
4	Korea	37	Male	-18.2	10.6	3	3	2	2	3	0	2	2	7	0
5	Korea	33	Male	-20.6	9.7	1	1	0	0	2	0	0	0	7	0
6	Korea	29	Male	-18.5	9.5	1	2	1	0	1	0	4	0	4	0
7	Korea	28	Male	-18.7	9.6	1	3	2	3	3	1	4	3	4	2
8	Korea	36	Male	-18.2	10.0	0	1	0	3	6	0	5	0	3	0
9	Korea	41	Male	-19.6	9.2	0	2	1	0	1	0	2	1	0	0
10	Korea	37	Male	-19.8	9.3	1	2	1	2	0	0	2	0	4	0
11	Korea	26	Female	-19.0	9.7	0	3	3	3	3	0	5	2	3	0
12	Korea	34	Female	-18.6	9.9	1	2	1	3	2	0	3	0	1	0
13	Korea	37	Male	-19.1	9.7	0	1	0	4	1	0	3	1	3	1
14	Korea	39	Male	-18.8	9.8	1	1	1	2	1	0	2	0	4	2
15	Korea	34	Female	-19.9	9.2	0	1	1	4	2	0	2	4	2	0
16	Korea	4	Male	-19.3	9.1	0	1	1	4	2	0	1	4	5	0
17	Korea	39	Male	-18.7	10.2	1	2	1	3	3	0	3	3	1	1
18	Korea	33	Female	-19.0	10.1	1	2	1	0	3	0	2	1	1	1
19	Korea	26	Female	-19.4	9.3	0	0	3	3	3	0	3	0	7	0
20	Korea	24	Male	-19.0	9.8	0	1	2	4	2	0	7	0	1	0
21	Korea	26	Male	-18.2	9.0	0	4	3	4	2	2	1	1	7	3
22	Korea	24	Female	-19.6	8.6	0	1	2	1	2	0	2	0	6	1
23	Korea	21	Female	-18.7	9.7	2	3	3	1	2	0	2	0	2	1
24	Korea	23	Male	-18.8	9.6	3	6	7	5	2	0	4	2	5	1
25	Korea	22	Male	-19.2	9.3	1	3	2	4	6	0	3	1	7	1
26	Korea	31	Female	-19.8	9.0	0	1	0	1	1	1	6	0	1	1
27	Korea	54	Female	-19.9	9.5	0	0	0	1	2	0	1	0	1	1
28	Korea	41	Male	-18.6	10.2	5	2	3	2	3	0	3	1	3	1
29	Korea	40	Male	-18.8	10.2	1	3	1	5	1	0	5	1	6	0
30	Korea	40	Male	-18.6	10.3	1	1	1	1	2	0	1	1	0	0
31	Korea	40	Male	-18.7	10.8	1	1	0	1	1	0	0	1	0	0
32	Korea	39	Female	-19.9	9.3	1	2	1	2	3	0	3	0	0	0
33	India	36	Female	-20.3	7.7	0	0	0	0	0	0	1	7	0	1
34	India	25	Male	-21.1	7.0	0	0	0	0	0	0	0	7	7	0
35	India	9	Male	-21.0	7.6	0	0	0	0	0	0	7	7	7	1
36	India	32	Female	-21.7	7.0	0	0	0	0	0	0	0	7	2	0
37	India	26	Male	-20.0	7.7	3	0	4	4	0	0	0	4	5	6
38	India	13	Male	-21.8	7.7	0	0	0	0	0	0	0	7	1	0
39	India	36	Female	-21.4	7.2	0	0	0	0	0	0	1	7	7	2
40	India	8	Female	-20.1	7.4	0	0	0	0	0	0	1	7	7	2
41	India	36	Male	-21.0	8.0	0	0	2	0	0	0	0	7	1	0
42	India	10	Female	-21.5	8.0	0	0	0	0	0	0	0	7	4	0
43	India	24	Male	-18.4	8.3	0	0	0	0	0	0	0	0	7	7
44	India	60	Male	-19.4	7.4	0	0	0	0	0	0	7	0	0	3
45	India	47	Male	-20.3	7.2	0	0	0	0	0	0	7	7	7	1
46	India	45	Female	-20.8	7.7	0	0	0	0	0	0	7	7	7	1
47	India	20	Female	-20.9	7.6	0	0	0	0	0	0	7	7	7	1
48	India	41	Male	-20.6	6.3	0	0	0	0	0	0	1	7	7	1
49	India	37	Male	-20.3	6.1	0	0	0	0	0	0	1	7	7	1
50	India	58	Male	-19.0	8.2	0	0	0	0	0	0	0	3	7	2
51	India	30	Male	-20.9	6.7	0	0	0	0	0	0	3	0	7	7
52	India	25	Male	-20.4	6.9	1	0	1	1	0	0	5	1	6	7
53	India	31	Female	-20.8	7.4	0	0	0	0	0	0	1	7	7	2
54	Mongolia	23	Female	-21.0	11.2	0	0	0	0	0	0	0	0	7	7
55	Mongolia	58	Female	-21.6	12.0	0	0	0	0	0	0	0	0	7	7
56	Mongolia	25	Female	-20.5	9.8	1	0	0	0	0	0	0	0	7	7
57	Mongolia	4	Female	-20.3	9.8	1	0	0	0	0	0	0	0	7	7
58	Mongolia	19	Female	-20.1	10.0	1	1	0	0	0	0	0	0	7	7
59	Mongolia	18	Female	-20.7	9.7	1	1	0	0	0	0	0	0	7	7
60	Mongolia	73	Female	-21.1	11.0	1	1	0	0	0	0	0	0	7	7
61	Mongolia	17	Male	-20.7	10.5	1	1	0	0	0	0	0	0	7	7
62	Mongolia	43	Male	-20.8	10.3	1	1	0	0	0	0	0	0	7	7
63	Mongolia	37	Female	-20.8	10.8	1	1	0	0	0	0	0	0	7	7
64	Mongolia	19	Female	-19.7	12.0	0	-	-	-	-	-	-	-	0	0

65	Mongolia	19	Male	-20.1	10.0	3	-	-	-	-	-	-	-	7	0
66	Mongolia	18	Female	-20.7	9.5	0	-	-	-	-	-	-	-	0	0
67	Mongolia	22	Female	-21.1	9.5	2	-	-	-	-	-	-	-	5	1
68	Mongolia	19	Female	-20.8	11.1	3	-	-	-	-	-	-	-	3	0
69	Mongolia	23	Male	-20.9	9.8	3	-	-	-	-	-	-	-	3	0
70	Mongolia	17	Female	-21.0	9.7	0	-	-	-	-	-	-	-	3	0
71	Mongolia	20	Female	-20.4	10.2	3	-	-	-	-	-	-	-	7	0
72	Mongolia	19	Female	-20.6	10.2	4	-	-	-	-	-	-	-	5	0
73	Mongolia	19	Female	-21.1	9.1	0	-	-	-	-	-	-	-	3	0
74	Mongolia	21	Female	-20.8	10.1	7	-	-	-	-	-	-	-	2	0
75	Mongolia	21	Male	-21.0	9.8	2	-	-	-	-	-	-	-	4	0
76	Mongolia	19	Female	-20.3	10.3	5	-	-	-	-	-	-	-	4	0
77	Mongolia	19	Female	-20.0	10.3	3	-	-	-	-	-	-	-	3	0
78	Mongolia	21	Female	-21.2	10.4	5	-	-	-	-	-	-	-	0	4
79	Mongolia	19	Male	-20.8	10.9	3	-	-	-	-	-	-	-	7	3
80	Mongolia	19	Male	-19.9	10.6	7	-	-	-	-	-	-	-	3	0
81	Mongolia	17	Female	-21.7	9.7	3	-	-	-	-	-	-	-	7	0
82	Mongolia	18	Female	-20.7	9.5	3	-	-	-	-	-	-	-	7	0
83	Mongolia	18	Female	-20.4	10.3	1	-	-	-	-	-	-	-	5	3
84	Mongolia	18	Male	-20.2	10.6	5	-	-	-	-	-	-	-	4	0
85	Mongolia	24	Female	-20.3	10.6	3	-	-	-	-	-	-	-	0	0
86	Mongolia	17	Male	-20.5	10.2	3	-	-	-	-	-	-	-	6	0
87	Mongolia	24	Female	-21.0	9.2	7	-	-	-	-	-	-	-	7	0
88	Mongolia	21	Male	-20.3	10.2	4	-	-	-	-	-	-	-	3	0
89	Mongolia	20	Male	-20.3	11.3	0	-	-	-	-	-	-	-	0	2
90	Mongolia	21	Female	-21.5	9.7	2	-	-	-	-	-	-	-	1	3
91	Mongolia	18	Female	-20.7	9.7	7	-	-	-	-	-	-	-	0	3
92	Mongolia	21	Male	-21.0	10.3	7	-	-	-	-	-	-	-	0	0
93	Mongolia	19	Male	-20.9	9.2	2	-	-	-	-	-	-	-	2	0
94	Mongolia	21	Female	-20.8	10.1	2	-	-	-	-	-	-	-	0	0
95	Mongolia	20	Female	-20.9	10.5	0	-	-	-	-	-	-	-	0	0
96	Mongolia	18	Female	-21.2	11.2	7	-	-	-	-	-	-	-	7	0
97	Mongolia	21	Female	-20.4	10.0	7	-	-	-	-	-	-	-	0	0
98	Mongolia	17	Female	-21.0	9.6	7	-	-	-	-	-	-	-	3	0
99	Mongolia	21	Female	-20.7	10.0	0	-	-	-	-	-	-	-	4	0
100	Mongolia	20	Female	-20.8	10.4	1	-	-	-	-	-	-	-	0	0
101	Mongolia	20	Female	-20.4	9.9	5	-	-	-	-	-	-	-	3	0
102	Mongolia	19	Male	-20.5	9.5	3	-	-	-	-	-	-	-	3	0
103	Mongolia	26	Female	-20.4	10.6	3	-	-	-	-	-	-	-	3	0
104	Mongolia	21	Male	-20.6	10.2	3	-	-	-	-	-	-	-	3	0
105	Mongolia	20	Female	-20.9	10.6	0	-	-	-	-	-	-	-	5	0
106	Mongolia	18	Female	-20.1	10.2	7	-	-	-	-	-	-	-	3	0
107	Mongolia	22	Female	-20.1	10.3	7	-	-	-	-	-	-	-	7	7
108	Mongolia	20	Female	-21.8	9.8	2	-	-	-	-	-	-	-	2	0
109	Mongolia	19	Female	-19.7	10.6	0	-	-	-	-	-	-	-	7	0
110	Mongolia	21	Male	-21.1	9.9	3	-	-	-	-	-	-	-	3	0
111	Mongolia	21	Male	-20.8	10.6	3	-	-	-	-	-	-	-	1	0
112	Mongolia	17	Female	-20.9	10.0	4	-	-	-	-	-	-	-	7	0
113	Mongolia	41	Female	-20.5	9.2	2	-	-	-	-	-	-	-	7	0
114	Mongolia	17	Male	-21.4	10.5	7	-	-	-	-	-	-	-	3	0
115	Mongolia	15	Male	-21.6	10.2	1	-	-	-	-	-	-	-	7	1
116	Mongolia	20	Male	-21.0	10.0	2	-	-	-	-	-	-	-	3	0
117	Mongolia	22	Female	-20.7	10.6	1	-	-	-	-	-	-	-	7	1
118	Mongolia	22	Male	-21.1	9.0	3	-	-	-	-	-	-	-	0	0
119	Mongolia	18	Female	-20.1	10.4	0	-	-	-	-	-	-	-	0	0
120	Mongolia	17	Female	-20.6	10.0	7	-	-	-	-	-	-	-	3	0
121	Mongolia	21	Male	-20.0	9.9	1	-	-	-	-	-	-	-	0	0
122	Mongolia	39	Female	-20.3	10.7	4	-	-	-	-	-	-	-	1	0
123	Mongolia	26	Male	-20.2	10.3	7	-	-	-	-	-	-	-	0	0
124	Mongolia	18	Female	-20.3	10.5	7	-	-	-	-	-	-	-	0	0
125	Mongolia	21	Male	-20.9	10.4	4	-	-	-	-	-	-	-	3	0
126	Mongolia	21	Female	-21.2	9.8	7	-	-	-	-	-	-	-	1	0
127	Mongolia	21	Male	-20.7	9.2	0	-	-	-	-	-	-	-	1	0
128	Mongolia	20	Female	-21.3	9.3	7	-	-	-	-	-	-	-	2	0
129	Mongolia	21	Male	-21.3	8.9	0	-	-	-	-	-	-	-	3	0
130	Mongolia	20	Male	-20.7	10.1	0	-	-	-	-	-	-	-	7	0
131	Mongolia	20	Female	-20.2	10.2	3	-	-	-	-	-	-	-	0	0

Title: Homogeneous diet of contemporary Japanese inferred from stable isotope ratios of hair
Authors: Soichiro Kusaka, Eriko Ishimaru, Fujio Hyodo, Gakuhari Takashi, Minoru Yoneda, Takakazu Yumoto, Ichiro Tayasu

Supplementary Table S6. Monthly expenditure of commodities of two-or-more-person households (Unit: Yen)

	Person per household	Fish and shellfish*	Beef	Pork	Chicken	Fresh milk	Butter and cheese	Eggs	Bean curd	Natto
Eastern Japan	3.21	7,055	1,191	2,136	873	1,417	414	665	494	347
Western Japan	3.10	6,413	2,123	1,933	1,163	1,420	329	716	520	190
Hokkaido	2.90	7,082	590	1,981	845	1,078	482	579	379	314
Tohoku	3.36	7,389	952	2,195	832	1,395	394	683	543	426
Kanto	3.04	6,851	1,430	2,207	972	1,442	494	655	490	328
Chubu	3.25	7,046	1,329	2,128	862	1,464	391	686	483	296
Kinki	3.06	6,691	2,663	2,134	1,230	1,476	389	767	482	174
Chugoku	3.09	6,669	2,019	1,820	1,113	1,470	364	715	496	184
Shikoku	3.00	6,371	2,163	1,732	1,102	1,525	278	693	575	157
Kyushu	3.13	5,732	1,720	1,909	1,187	1,206	300	683	517	220
Hokkaido	2.9	7,082	590	1,981	845	1,078	482	579	379	314
Aomori	3.25	7,086	846	2,009	822	1,194	371	574	465	381
Iwate	3.32	7,545	750	1,922	751	1,367	323	644	652	408
Miyagi	3.42	7,287	995	2,190	767	1,528	451	718	554	443
Akita	3.25	8,419	902	2,328	1,058	1,269	320	635	529	407
Yamagata	3.67	7,582	1,607	2,443	837	1,573	414	677	572	442
Fukushima	3.29	6,925	767	2,299	835	1,373	421	777	503	453
Ibaraki	3.17	6,649	975	1,993	874	1,382	402	636	455	382
Tochigi	3.19	6,746	1,057	2,015	802	1,504	426	670	477	429
Gumma	3.09	5,933	796	1,825	697	1,377	361	604	450	393
Saitama	3.05	6,446	1,317	2,213	960	1,402	464	651	500	327
Chiba	3.05	6,890	1,435	2,113	939	1,451	447	602	476	325
Tokyo	3	7,058	1,729	2,303	1,093	1,459	557	658	528	303
Kanagawa	2.97	7,206	1,658	2,332	1,044	1,469	560	698	481	312
Niigata	3.43	7,164	806	2,345	753	1,457	417	710	476	332
Toyama	3.31	7,690	1,325	1,979	736	1,497	389	609	508	366
Ishikawa	3.25	8,160	1,873	2,096	824	1,510	373	696	507	270
Fukui	3.34	8,155	2,221	1,967	1,097	1,586	343	775	538	282
Yamanashi	3.13	6,595	1,003	2,297	815	1,491	406	657	421	308
Nagano	3.18	6,788	886	2,188	746	1,408	439	691	449	336
Gifu	3.19	5,797	1,407	1,915	867	1,370	360	688	473	251
Shizuoka	3.16	7,162	949	2,237	920	1,342	397	672	519	287
Aichi	3.23	5,906	1,495	2,130	1,004	1,511	393	677	460	230
Mie	3.19	7,291	2,434	2,114	1,149	1,555	373	740	463	203
Shiga	3.26	6,976	2,754	2,138	1,226	1,736	404	779	538	230
Kyoto	3.07	6,975	2,636	2,180	1,279	1,578	401	739	503	182
Osaka	3	6,501	2,559	2,104	1,201	1,351	375	744	459	163
Hyogo	3.08	6,622	2,679	2,126	1,279	1,538	421	820	501	192
Nara	3.15	7,250	2,946	2,182	1,170	1,555	392	751	514	162
Wakayama	3.01	6,875	3,030	2,238	1,187	1,513	278	743	418	109
Tottori	3.35	6,961	1,541	1,820	1,038	1,645	331	781	554	186
Shimane	3.2	7,010	1,629	1,887	1,131	1,532	305	780	623	197
Okayama	3.15	6,305	1,944	1,931	1,104	1,453	352	644	464	174
Hiroshima	3.02	6,561	2,158	1,801	1,131	1,442	402	735	486	190
Yamaguchi	2.99	7,064	2,204	1,687	1,108	1,448	347	712	474	179
Tokushima	3.01	5,952	2,411	1,802	1,104	1,651	275	600	683	138
Kagawa	2.99	6,313	2,196	1,750	1,097	1,772	301	645	533	166
Ehime	3.02	6,164	2,140	1,741	1,102	1,462	275	718	542	156
Kochi	2.95	7,246	1,919	1,626	1,108	1,208	257	800	581	163
Fukuoka	3.09	6,312	1,993	1,886	1,273	1,291	335	723	460	244
Saga	3.48	5,990	2,071	1,884	1,213	1,331	301	672	535	196
Nagasaki	3.09	6,487	1,622	1,923	1,087	1,203	296	745	445	217
Kumamoto	3.23	5,373	1,945	1,762	1,236	1,218	282	715	447	275
Oita	3.02	5,732	2,147	1,830	1,254	1,223	285	670	519	222
Miyazaki	2.9	5,621	1,634	1,880	1,366	1,176	315	585	538	233
Kagoshima	2.96	6,340	1,494	2,306	1,375	1,261	244	675	547	233
Okinawa	3.25	4,000	856	1,801	691	946	342	676	648	143

* "Fish and shellfish" includes freshwater fish.
